# Supplementary material for: Determining a reliably visible and inexpensive surface fiducial marker for use in MRI: a research study in a busy Australian Radiology Department
Source: BMJ Open. 2019 Aug 1;9(8):e027020. doi: 10.1136/bmjopen-2018-027020 (PMC6688688; doi:10.1136/bmjopen-2018-027020)
Supplement: Author's manuscript [file bmjopen-2018-027020.draft_revisions.pdf]

BMJ Open is committed to open peer review. As part of this commitment we make the peer review history of every article we publish publicly available.

When an article is published we post the peer reviewers' comments and the authors' responses online. We also post the versions of the paper that were used during peer review. These are the versions that the peer review comments apply to.

The versions of the paper that follow are the versions that were submitted during the peer review process. They are not the versions of record or the final published versions. They should not be cited or distributed as the published version of this manuscript.

BMJ Open is an open access journal and the full, final, typeset and author-corrected version of record of the manuscript is available on our site with no access controls, subscription charges or pay-per-view fees (http://bmjopen.bmj.com).

If you have any questions on BMJ Open's open peer review process please email editorial.bmjopen@bmj.com

BMJ Open

Reliably Visible and Inexpensive surface Fiducial Marker options for Magnetic Resonance Imaging

Journal:	*BMJ Open*
Manuscript ID	bmjopen-2018-027020
Article Type:	Research
Date Submitted by the Author:	15-Oct-2018
Complete List of Authors:	Izatt, Maree; Queensland University of Technology, Biomechanics & Spine Research Group<br>Lees, Deborah; Queensland University of Technology, Biomechanics & Spine Research Group<br>Mills, Susan; Mater Misericordiae Brisbane Ltd, Mater Medical Imaging<br>Grant, Caroline; Queensland University of Technology, Biomechanics & Spine Research Group<br>Little, Judith; Queensland University of Technology, Biomechanics & Spine Research Group
Keywords:	Magnetic resonance imaging < RADIOLOGY & IMAGING, MRI, fiducial marker, surface marker, MRI sequence, marker visibility

SCHOLARONE™
Manuscripts

Title:

Reliably Visible and Inexpensive surface Fiducial Marker options for Magnetic Resonance Imaging

Authors:

Maree T. Izatt[1], B.Phty m.izatt@qut.edu.au
Project Coordinator, (ORCID ID: 0000-0002-4926-3077)

Deborah Lees[1], M.Tech (Chiro), MBBS, FRCS Traumo & Orth deborah.lees@health.qld.gov.au
Spine Fellow, Orthopaedic Surgeon

Susan Mills[2], B.App.Sci (MI) susan.mills@mater.org.au
MRI Unit Manager

Caroline A. Grant[1], PhD BEng ca.grant@qut.edu.au
Postdoctoral Research Fellow, (ORCID ID: 0000-0002-5128-9903)

J. Paige Little[1], PhD BEng j2.little@qut.edu.au
Senior Research Fellow, (ORCID ID: 0000-0001-8377-5343)

Affiliations:

[1] Biomechanics and Spine Research Group, Institute of Health and Biomedical Innovation at Centre for Children's Health Research, Queensland University of Technology, Brisbane, Australia

[2] Mater Medical Imaging, Mater Misericordiae Ltd, Brisbane, Australia

Corresponding Author:

Dr J P Little Email: j2.little@qut.edu.au; Phone: +61 7 3069 7325

Summary Box

- A reliably visible and inexpensive surface fiducial marker for MRI was required as an alternative to the single-use commercial markers currently available.
- A search of the literature did not provide any alternative so the current study sought to find a cheap, easily sourced alternative marker for use in clinical imaging departments or research projects utilising MRI.
- Four items were seen reliably on the five most commonly requested MRI sequences; fish oil capsule, vitamin D capsule, paint ball pellet and sushi soy sauce tube.

For peer review only - http://bmjopen.bmj.com/site/about/guidelines.xhtml

- The Vitamin D capsule proved to be ideal, with an excellent balance between availability, size, cost, and quality of the visualised marker for commonly used MRI sequences.
- Clinical MRI departments and researchers requiring the use of surface fiducial markers now have cheap alternative marker options to use in place of the relatively expensive single-use commercial fiducial marker.

Structured Abstract

Objectives

Single use commercial surface fiducial markers are used in clinical imaging for a variety of applications. The current study sought to find a new, reliably visible, easily sourced and inexpensive fiducial marker alternative for use with magnetic resonance imaging (MRI).

Design

Five commonly requested MRI sequences were determined (3D T1-weighted, T1 coronal, 3D T2-weighted, T2 fat suppressed, Proton density), to examine the visibility of 18 items (including a commercial fiducial marker).

Setting

Clinical 3T MRI scanner in Tertiary Public Hospital and University Biomedical Engineering research group

Interventions

18 marker alternatives were scanned using five common MRI sequences. Images were reformatted to obtain both an image through the mid-height of each marker and a maximum intensity z-projection image over the volume of the marker. Variations in marker intensity were profiled across each visible marker and a visibility rating defined.

Main outcome measures

Finding cheap easily sourced alternative marker options that are reliably visible on common clinical MRI sequences. Demonstrating a clear intensity contrast between the marker and the adjacent tissue.

Results

The fish oil capsule, vitamin D capsule, paint ball pellet, soy sauce sushi tube and commercial markers were typically visible to a high quality on all the imaging sequences and demonstrated a clear differential in intensity contrast against the adjacent tissue. Other common items, such as plasticine ‘play doh’ and a soft ‘Jelly baby’ sweet were surprise candidates, demonstrating high quality visibility and intensity contrast for the 3D T1-weighted sequence.

Conclusions

Depending on the basis for referral and MRI sequence chosen, four alternative fiducial markers were determined to be inexpensive, easily sourced and consistently visible. Of these, the Vitamin D capsule provided an excellent balance between availability, size, cost, usability and quality of the visualised marker for all the commonly used MRI sequences analysed.

For peer review only - http://bmjopen.bmj.com/site/about/guidelines.xhtml

Strengths and Limitations

- This manuscript is the first to test for easily sourced and economical items to find reliably visible surface fiducial marker options for common MRI sequences
- The anterior surface of a healthy female thigh was used to scan the seventeen different items in a 3T MRI scanner as this was considered superior to using a saline phantom object
- Both a qualitative analysis of visibility and a quantitative analysis of intensity contrast to adjacent tissues were performed against the gold standard of a commercial surface fiducial marker
- Despite using only one type of commercial fiducial marker on one representative human limb, the current study provides unique and inexpensive alternative marker options for the most commonly used MRI sequences.

Keywords
Magnetic resonance imaging, MRI, fiducial marker, surface marker, MRI sequence, marker visibility, marker intensity, low cost marker, inexpensive marker

For peer review only

Introduction:

Fiducial markers used in the context of clinical medicine may be implanted in the body or placed on the surface and are also utilised in research for a variety of applications. Placed in the field of view, they display as distinct regions of high intensity to assist in pinpointing specific anatomical landmarks or pathologies on the acquired clinical images. Surface markers placed on a patient's skin can also provide a frame of reference for registration of medical images acquired using multiple imaging modalities, such as photography, computed tomography (CT) and magnetic resonance imaging (MRI) performed for concomitant pathologies involving multiple specialities.

In radiotherapy, fiducial markers are increasingly being used to identify the tumour site, permitting image registration and assisting with guidance for treatment planning.(1-3) Oil-based surface markers have been shown to compare favourably to solid markers in terms of their contrast to noise ratio, resulting in excellent visibility.(3) Prostate marker studies also revealed that implanted titanium seeds left star/streak artefacts on CT imaging and could not be accurately localised on MR due to negative contrast (black holes).(4-6) Fiducial markers also perform a valuable role in guiding anatomical identification in musculoskeletal studies, particularly kinematic gait analyses where the location of surface markers on both mathematical gait models and the accompanying 3D MRI images provides valuable subject-specific simulation parameters.(7)

Commercially available fiducial surface markers (i.e. non-implantable) are available in a range of sizes and shapes for use in the wide variety of clinical imaging applications. They are self-adhesive and have a low, flat profile, making them comfortable for the patient, even for an extended period of time. While these markers provide excellent utility, they present a substantial expense to radiology departments, with a review of the current markets showing prices ranging from $420 to $600 USD for a box of 100 (MR Spots® and IZI multi-modality, excluding shipping and tax). Being a single use product they are a considerable expense to the running costs of imaging departments or research teams utilizing MRI and requiring numerous markers.

A review of the literature to find materials that are visible in MRI revealed a paucity of publications. The majority of papers examining substance visibility were focused primarily on locating a variety of penetrating or ingested foreign bodies and just a few papers explored implanted marker options for tumour boundary markers prior to excision or radiotherapy planning.(2, 4-6, 8) Foreign bodies that had been located, ranged in material from fish and chicken bones, batteries, plastics, coins, metal and wood splinters (9-12) and it should be noted that some materials were missed even with the high sensitivity of modern MRI and CT.(13, 14) Pattamapospong *et al* (15) published results regarding the visibility of a number of substances on X-ray, CT and MRI and demonstrated a 100% specificity but only 58% sensitivity for fresh wood, dry wood, glass, plastic and porcelain using MRI. Wood as a material has been shown to be particularly difficult to visualise and whilst well detected with ultrasound, wood can easily be missed or misdiagnosed on CT.(13) Reddy *et al* (11) made this point poignantly in their case report of a thorn (retained foreign body) in the extradural space missed on CT which resulted in a granuloma forming with subsequent neurological deficit. MR has been shown to be more reliable in successfully imaging wood,(13) but is less capable in the detection of smaller wood splinters. Wax crayons have proven to be easily visible on both CT and MRI with high attenuation noted on CT and a signal void on

For peer review only - http://bmjopen.bmj.com/site/about/guidelines.xhtml

both T1 and T2 MR images. Interestingly, it was noted that crayon colour influenced the degree of visibility. Pigments used in crayons and in paints are derived from iron oxide (red, orange, yellow and brown), organometallic complexes (pink and green), carbon (black) and titanium dioxide (white). These differences resulted in distinctive colours as well as MRI appearance.(16) Injectable facial fillers (hyaluronic acid, collagen and polyalkylimide-polyacrylamide hydrogels), silicone and calcium hydroxyapatite have also been well visualised on MRI but can be confused with malignant features, so are not considered good marker substances.(17) Metal is well visualised on many modalities (9) but due to its ferrous properties, is contra-indicated in the field of MRI. Metal, even if MR compatible, results in significant image attenuation and artefact. For these reasons, an iron tablet as a makeshift marker may be well visualised but the resulting artefact renders it unacceptable. Studies looking at the visibility of plastic, stone, glass and graphite revealed that both glass and plastic foreign bodies are not consistently visible on MRI or plain radiographs; despite showing up well on CT.(15, 18) MR was able to detect stone pieces larger than 0.5mm and graphite pieces of 1mm with comparable brightness, although stone was again seen better on CT.(18)

A review of the physics of MRI suggested materials with high water or fat content were likely items that would be readily detectable. Difficulties arise currently when the size of the marker is such that it obscures the underlying anatomy, thus the size and shape of potential markers is also a consideration. Fiducial markers should be easily identifiable and clearly visible on clinical images, allowing the target anatomy or volume to be clearly and completely visualised. Therefore a critical objective of this study was to assess the validity of various 'everyday' items, which could be easily and economically sourced, and provide a reliably visible fiducial marker as an alternative to the comparatively expensive, single use commercial fiducial markers.

Methods:

Seventeen everyday items and a commercial fiducial marker were selected for analysis (Figure 1). The important considerations relating to the selection of a suitable surrogate for commercial fiducial markers were identified and are outlined in Table 1. In addition to the commercial fiducial marker, two of these items had been used in our local hospital medical imaging department; the paint ball for MR scans in a pilot research project, and the fish oil capsule which was in regular use to avoid using a commercial marker whenever possible. While these two non-commercial markers provided excellent results in terms of intensity contrast and visualisation on MR images, to date a definitive comparison of the image contrast provided by these items has not been undertaken and these alternatives did not meet all the desired requirements of the ideal alternative fiducial marker.

For peer review only - http://bmjopen.bmj.com/site/about/guidelines.xhtml

Table 1. Important considerations relating to the choice of a suitable fiducial marker for MR imaging

Factors for consideration	
**Accessibility**	Easily acquired, readily replaced, and preferably locally sourced without high shipment costs or transit time
**Use**	Sufficiently robust to tolerate body weight if placed between the participant and scanner bed<br>Suitably compliant or flat to ensure minimal discomfort to the patient if marker is in a load bearing location (e.g. Posterior superior iliac spine for a supine MRI)
**Biochemistry**	Chemically inert, hypoallergenic
**Economy**	Low acquisition cost per marker given markers are single use items
**Geometry**	Sufficiently small not to obscure relevant anatomy;<br>Easily handled to allow accurate placement
**Medical Physics**	Sufficient hydrogen content to permit high intensity contrast to surrounding tissue
**Fixation**	Reliably affixed to the patient's external anatomy

Table 2. MRI sequence details

	Imaging Protocol	Acquisition plane	Field of View (FOV) (mm)	Repetition time (TR) / Echo time (TE) (msec)	Resolution (Pixel width x height x slice spacing, mm)
**1**	Proton density (PD)	Coronal	300 x 300 x 44	2,435/35	0.1465 x 0.1465 x 2.2
**2**	T1 weighted, Spin echo	Coronal	300 x 300 x 44	661/10	0.1465 x 0.1465 x 2.2
**3**	T1 weighted, 3D Fast field	Sagittal	300 x 300 x	25/1.4	0.469 x 0.469 x 0.5

For peer review only - http://bmjopen.bmj.com/site/about/guidelines.xhtml

	echo (FFE)		175		
**4**	T2 weighted, fat suppressed	Coronal	300 x 300 x 44	4,448/65	0.195 x 0.195 x 2.2
**5**	T2 weighted, 3D	Sagittal	300 x 300 x 175	2,473/72	0.469 x 0.469 x 0.5

Imaging Details:

The selection of items underwent MR scanning in a clinical medical imaging department using a 3-T Philips Achieva MRI scanner. Scanning parameters vary between different machine manufacturers and even between different scanner models from the same manufacturer. However, this particular scanner was selected as it services a busy hospital radiology department in a metropolitan city, and thus, provides a broad range of exploratory and treatment-based MRI scanning services. Further to this, the 3-T magnet strength is a typical specification for scanners in tertiary care facilities in Australia and internationally.

Following the advice from the senior MR radiographers, five different sequences were performed (Table 2). These sequences were selected to cover the breadth of MR imaging studies typically performed with a requirement for inclusion of surface markers to isolate internal anatomy or an area of interest. To ensure a realistic intensity contrast between marker and tissue could be obtained, all tested markers were attached to the anterior thigh of a healthy female participant, aged 27 years, who had provided informed consent. A radiofrequency coil (8-channel knee coil) was positioned beneath the thigh and the markers attached to the anterior thigh surface (Figure 2). Due to physical size constraints, the marker selection (

Figure 1) was attached to the volunteer's leg in two separate acquisitions (Figure 2A). In all the MR sequences detailed in Table 2, the scanning parameters were constant for both acquisitions.

Images were saved in Digital Imaging and Communications in Medicine (DICOM) format and analysed using ImageJ (U. S. National Institute of Health Open-source software, Maryland, USA; https://imagej.nih.gov/ij/). A similar process was utilised to assess the results from each of the five different imaging sequences (Table 2) and involved first viewing the 3D stack as a segmented volume using the ImageJ Plugin, 'Volume Viewer 2.01' (K U Barthel, Internationale Medieninformatik, HTW Berlin, Germany) (Figure 3).

In the first instance, a qualitative evaluation of the marker visibility was carried out to subjectively assess the relative ease with which each marker could be visualised and the marker edges demarcated. Viewing the data as a 3D volume permitted an initial assessment of which markers exhibited an intensity that was visible on MR images (Figure 3). If the marker was visible, the visibility was rated on a 3-point Likert scale with 3 representing 'very clear', 2 representing 'edges visible but fuzzy' and 1 representing 'not easily visible'. If a visibility rating of one was observed, this low rating suggested that while the marker was discernibly visible on the MR images, the quality of visibility was poor and the marker would not be recommended for use in clinical scanning using that particular MR sequence.

For peer review only - http://bmjopen.bmj.com/site/about/guidelines.xhtml

Assessment of Marker Visibility:

In all cases, MR images were reformatted and processed as 16-bit images, with an intensity range from 0 to 65,536. A quantitative evaluation of the marker quality was carried out to assess the visibility, artefacts and distortion of marker boundaries created by each item on each MRI sequence. This evaluation was carried out using the steps outlined below and shown visually in Figure 4.

1. Reformatted Image Stack: The DICOM stacks were reformatted to create a sequential stack of transverse reslice images, with consideration of the relevant pixel and slice spacing for each dataset (Table 2).
2. Intensity Profile Plot: Using the 3D volume reconstructions for each MRI sequence as a reference (Figure 3), the reformatted transverse reslice through the mid-height of each marker was located (Figure 4, B). A line selection (Figure 4, C) was drawn through the marker, such that the mid-point on the line was positioned at the interface between the marker and the skin and the endpoints were located in air and muscle tissue, respectively. The intensity profile along this line selection (Figure 4, D) was used to objectively compare the marker signal and visibility. The peak intensity in the marker, minimum intensity at the interface between the marker and tissue, mean intensity in the fat tissue and mean intensity in the muscle tissue were recorded from the profile.
3. Maximum Intensity Z-projection: A two-dimensional (2D) image with each pixel intensity equivalent to the maximum intensity along a Z trajectory over a given range of stack images was created (Figure 4, E) The image range was defined to encompass the upper and lower limits of the marker, as viewed on the 3D volume reconstruction (Figure 4 A, orange broken line).
4. Intensity Histogram and Maximum Marker Intensity: A segmented line tool (Figure 4 F) was used to demarcate the outer profile of the marker on the 2D Z-projection image (Figure 4 E) A histogram showing the distribution of maximum intensities within this profile for the marker was used to record the maximum and mean marker intensity (Figure 4 G).

In some instances (Figure 3: T1_3D – Chewing Gum; T1 Coronal – Eraser) the intensity range exhibited by the marker was very low in comparison to the background intensity, making the marker boundaries difficult to differentiate from the surrounding regions. However, these markers were still included in the analysis as it was possible to create a profile plot (Figure 4 D) on a transverse plane through the mid-height of the marker, with a measurable intensity gradient across the marker.

Assessment of Intensity Values:

In order to objectively assess whether the markers provided an acceptable visualisation on the images produced for each MR sequence analysed, three criteria were defined to assess the results. It was expected that the markers should; a) Provide a pixel intensity that was sufficiently above the background intensity of air to result in a clear visual contrast; b) Demonstrate a pixel intensity ratio at the interface between the marker and adjacent epidermis/fat tissue (i.e. peak marker intensity divided by interface intensity/mean fat tissue intensity) greater than 1; and c) Demonstrate a pixel intensity

For peer review only - http://bmjopen.bmj.com/site/about/guidelines.xhtml

ratio between the marker and the muscle tissue (i.e. peak marker intensity divided by mean muscle intensity) greater than 1.

The ratios were intended to provide a numeric value that aligned with the perceptual and visual decision making process a person undertakes when determining whether a marker is visualised at an acceptable quality. To indicate the quality of marker visualisation, this ratio of unity was defined after viewing all markers over the five MRI sequences and in combination with the visual assessment of visibility rating.

Depending on the MRI sequence, the fat and muscle tissue adjacent to the marker displayed over different intensity ranges. The ratio between the fat or muscle tissue and marker intensity was considered critical to be able to differentiate the marker from its surroundings. For this reason the intensity ratio was considered of more importance than the absolute value of the pixel intensity.

Results:

Of the 18 markers investigated, five were consistently visible for all imaging sequences, but this visibility was of differing quality (Figure 5). The Fish Oil (FO), Vitamin D (D), Paintball (PB), Soy Sauce Sushi Tube (ST) and Commercial Marker (CM) were typically visible to a high quality (i.e. rating 2 or 3) for all sequences (Table 3). A visibility rating of 1 on only one of the MR sequences was observed for the Eraser (E), Chewing Gum (CG), Lifesaver (LS), and Coffee Bean (CB), demonstrating that while these markers were visible, the quality of visibility was poor. Surprisingly, the Pl marker was visible to a high quality (rating 3) for the T1_3D sequence, but had little (rating = 1 for PD sequence) or no visibility for the other sequences. Similarly, the Jelly Baby was also remarkable in being well visible on the T1_3D sequence with a rating of 2, but had no visibility scored for the other MR sequences.

While a mean visibility rating >0 indicated that the marker could be seen on at least one of the MRI sequences, a mean visibility rating greater than 2 was preferable as it indicated a consistently good quality visibility on all of the MRI sequences performed. A mean visibility score greater than 2 was observed for the PB, FO, D and CM markers (Table 3).

Table 3. Visibility rating for each marker on each MRI sequence. If not visible, the marker rating = 0. If visible, marker visibility rating was either 1 ('visible, but not easily'), 2 ('edges visible but fuzzy') or 3 ('very clear').

	Proton Density	T1 Coronal	T1_3D	T2 Fat_Sup	T2_3D	MEAN RATING
**Commercial Marker**	2	2	3	3	2	2.4
**Fish Oil**	2	2	3	2	3	2.4
**Vitamin D**	2	2	3	3	3	2.6
**Paint Ball**	2	2	3	3	3	2.6

For peer review only - http://bmjopen.bmj.com/site/about/guidelines.xhtml

**Soy Sauce Sushi Tube**	2	2	3	2	1	2.0
**Plasticine**	1	0	3	0	0	0.8
**Jelly Baby**	0	0	2	0	0	0.4
**Coffee Bean**	1	0	0	0	0	0.2
**Eraser**	0	1	0	0	0	0.2
**Chewing Gum**	0	0	1	0	0	0.2
**Lifesaver**	0	0	0	1	0	0.2
**Fruit Lolly**	0	0	0	0	0	0
**BluTak**	0	0	0	0	0	0
**Magnesium**	0	0	0	0	0	0
**TicTac**	0	0	0	0	0	0
**Mentos**	0	0	0	0	0	0
**Mintie**	0	0	0	0	0	0
**Nail Polish**	0	0	0	0	0	0

Figure 5 shows a summary of the visible markers on the five MRI sequences. In each case, the intensity range for the image was selected such that the intensity contrast within the image could best display the marker. Note however, these images do not include image filtering or contrast enhancement. Viewing the reslice images in Figure 5 in combination with the visibility ratings in Table 3 demonstrated that the best quality marker visibility for the T1_3D, T1 coronal, T2 Fat_Sup and Proton Density sequences was the PB, ST, FO and D; and for the T2_3D sequence was the PB, FO and D. The CM was best visualised on the T1_3D and T2 Fat_Sup sequences.

The maximum and mean pixel intensity over the volume of the visible markers varied depending on the particular sequence, with the PD, T2_FatSup, T2_3D, T1_Coronal and T1_3D sequences demonstrating a maximum intensity of 363 (D), 483 (ST), 1,480 (FO), 900 (ST) and 1,664 (ST), for each sequence, respectively (Figure 6).

Intensity ratios between the maximum marker intensity and the mean intensity in either the fat tissue or muscle tissue are shown in Figure 7.

For the PD sequence, while the CM, FO, D, PB and ST markers were easily visible (Figure 5, Figure 7), the marker intensity was numerically similar to the fat tissue, resulting in marker-to-fat ratios near or below one. The PL and CB were less easily differentiated from the nearby fat tissue (Figure 7).

For peer review only - http://bmjopen.bmj.com/site/about/guidelines.xhtml

For the T2 fat suppressed sequence, there was a distinct differential between the maximum intensity in the marker and the mean intensity in the fat tissue, making all observable markers (CM, FO, D, PB, ST and LS) clearly visible (Figure 7).

The commercial marker (CM) was not clearly visualised for the 3D T2-weighted sequence (Table 3, Figure 5), and had a marker-to-fat ratio <1 (Figure 7). While the ST tended to result in good visibility (Table 3, Figure 5), for the 3D T2-weighted sequence the marker intensity was less than the muscle tissue and not easily differentiated from the background air intensity. Aside from this, the 3D T2-weighted sequence showed an intensity ratio >>1 and thus clear intensity contrast for the marker-to-muscle ratio (>1) and marker-to-fat ratio, for all markers.

While the eraser (E) was visible on the T1 coronal sequence, the intensity ratios suggested that the intensity contrast between the marker and adjacent muscle/fat was not of a good quality (Table 3, Figure 5). This was supported by the observed intensity ratio <1 for both the marker-to-muscle and marker-to-fat ratios (Figure 7). For the other visible markers, the intensity ratios were all ≥ 1.
With the exception of the chewing gum (CG) and jelly baby (JB), the intensity ratios for marker-to-fat were all >1, for the 3D_T1-weighted sequence. In the case of the PB and ST marker, since these were comprised of a non-oil based substance, the intensity contrast and marker-to-fat/marker-to-muscle intensity ratios were >>1 (Figure 7). These latter two markers also resulted in a high visibility rating (Table 3, Figure 5).

Discussion

Fiducial markers have a number of important roles within radiology departments. They are used for calibration of the imaging equipment, providing a reference measurement for templating software, locating specific boundaries for radiotherapy planning, orienting an image and very importantly, to identify a specific area of interest in the case of diagnostic queries. Markers are vital as a consistent reference source across a range of modalities required for multidisciplinary team input and medical management. It is thus essential to have a marker that is detectable and clearly discernible from bone and soft tissues when placed in the visual field for a number of MR sequences and protocols whilst not resulting in any image attenuation. Single use, commercially available fiducial markers make up a significant component of the imaging department's outgoings and in an attempt to reduce costs, the current study was a practical exercise designed to image a number of commonly available everyday items to assess their suitability to function as an effective surface fiducial MRI marker substitute.

Personalised musculoskeletal kinematic models have become key investigations for a range of different population groups. Sports people looking to optimise their technique, amputees testing new prostheses, as well as people with musculoskeletal disorders use dynamic studies to gain a better understanding of their specific biomechanics and to quantify the outcomes of any interventions. Correlation of the dynamic studies with personalised clinical imaging allows for highly specific kinematic evaluation but is heavily dependent on the quality and accuracy of anatomical parameter modelling.(7) The current study

For peer review only - http://bmjopen.bmj.com/site/about/guidelines.xhtml

also aimed to provide additional marker options useful for this purpose as well as for clinical pathologies and research projects utilizing MRI.

A single use, commercially available surface fiducial marker was evaluated in this study alongside seventeen potential alternatives and its performance was surprisingly less impressive than some of the more common and affordable items tested. The commercial marker is considered prohibitively expensive (AUD$ 6 to 10 per marker) and is therefore used selectively for specific neurosurgical imaging needs only. Makeshift markers of various types have been anecdotally trialled in clinical radiology departments over the years. Discussions with our hospital imaging staff revealed that almonds, Vitamin E capsules and condiment packets had previously been trialled. Packaging rupture was a risk and unfortunately these makeshift markers caused issues with patient comfort, inconsistent visibility on imaging, and distortion of the pathology.

Fluids are more readily identifiable on MRI and lipid-based markers have proven to be consistently reliable.(8, 19) The capped sushi soy sauce tubes tested in the current study were reliably visible on all the MRI sequences analysed and were considered by the MR radiographers as a useful alternative marker candidate. They suggested one or more of the fish-shaped tubes could act as a type of “pointer” to highlight a small lesion or point of interest without obscuring it. It was also considered sufficiently small to not compress or cover the relevant anatomy. In addition, these fish shaped soy sauce tubes could be purchased in bulk, thus proving to be a suitable surface marker alternative from the budget perspective for selected cases.

The paint ball pellet performed well, demonstrating good visibility on all the common MRI sequences. It represents good value at a cost of AUD$12 to 20 per jar of 500 pellets. Concerns remain however, with regards to the risk of damage to equipment and property if the paint ball ruptured as the pellets are designed to burst at low force to prevent injury during game play. Due to this inherent risk of rupture, it was considered an unacceptable risk.

Oil-based capsules, particularly fish oil, are most commonly used as a commercial marker alternative (20) and are available in bulk, providing excellent value per capsule. But due to their large size (~26mm long), they are difficult to use accurately for small pathologies or on the extremities. Previous departmental experience, which has been confirmed by this study, has shown that both the fish oil capsules as well as the vitamin E capsules show up brightly on MR sequences and are comfortably and easily secured to the skin with tape. The vitamin E or fish oil capsules have been noted to be particularly useful for imaging larger body parts such as the thigh or abdomen where imaging slices are often thicker (5mm or more) such that a smaller marker may be difficult to locate. Concerns however were raised that if used in a weight bearing situation that caused rupture, the oil was difficult to clean off the patient and scanner, as well as the fish oil leaving a persistent unpleasant odour.

Performing successful MRI on children can be challenging and may require a creative approach, especially in the younger age groups. Children who have experienced a lot of medical interventions can be fearful of any and all medical procedures. Therefore we suggest it may be a great advantage to be able to produce a ‘jelly baby’ sweet to apply as a fiducial imaging marker when a T1_3D sequence was required, or alternatively a fish shaped soy sauce tube or Vitamin D capsule if this particular sequence was not appropriate.

For peer review only - http://bmjopen.bmj.com/site/about/guidelines.xhtml

An ideal alternative marker that proved to be easily visible on all the common MR sequences tested, was the Vitamin D capsule. Vitamin D capsules are available to purchase at a cost of AUD$36 for 400 capsules, which makes them affordable, disposable and readily replaceable. Vitamin D provided a bright MRI marker virtually identical to the visibility of the fish oil capsule, but was significantly smaller in size (~13mm long), making it more appealing when markers are required in imaging of the extremities, face or small pathologies. Current departmental experience has demonstrated that fish oil, vitamin E and vitamin D capsules have sufficient resilience to withstand the demands of being used as a marker, even in prolonged weight bearing situations.

Depending on the reason for imaging and the specific pathology, different MRI sequences are chosen to provide well visualised tissue contrast. For example, to demarcate the location of a tumour, a 3D T1-weighted/T2-weighted or T1 Fat Suppressed protocol would be considered more appropriate while for exploratory imaging relating to tissue infection, a T1 coronal or T2 Fat Suppressed protocol is preferable. Conversely, when MR imaging is requested for musculoskeletal conditions, a Proton Density sequence is the preferred protocol to provide high intensity contrast in both fat and fluid in the bone. As such, this study provides evidence that there may not be one marker that is best suited to all imaging sequences and individual markers may provide good quality results for some but not all sequences.

A single commercial fiducial marker was analysed in the current study however it was the brand used in our clinical imaging facility making it the most relevant marker to examine. A single human participant was used but we considered this to be superior to a saline phantom. Marker visibility was analysed by a single observer but typically a single examiner views images to make judgements on visible anatomy.

In the current study, the FO, PB and D markers were all clearly visible on each of the five sequences and typically demonstrated well-defined intensity contrast with the adjacent fat/tissue. On the basis of intensity contrast, these three markers would be recommended in place of a commercial marker (CM) for use as an external fiducial marker. However, as noted in Table 1, in addition to MRI visibility of the marker, consideration of the cost, accessibility and useability of the surrogate marker are of equal importance. In light of this, the PB is less accessible, requiring specific online ordering or purchase at custom stores and the FO, while easily sourced, is too large for some applications and brings with it the risk of capsule rupture and the release of a pungent odour. Vitamin D capsules are economically purchased, readily available and bring no detrimental outcomes should they rupture. Anecdotally, the authors of this study have since used Vitamin D capsules routinely for supine musculoskeletal studies of the spine requiring extended sessions in the MR scanner and have not had any degradation or rupture, with excellent visibility of these markers observed on the resulting MR images.

For peer review only - http://bmjopen.bmj.com/site/about/guidelines.xhtml

Figure Titles

Figure 1. Items investigated as surrogates for the commercially available fiducial markers. Note that a commercial fiducial marker (IZI Multi-Modality Fiducial Marker, LifeHealthCare, Australia) was included as a benchmark for comparison.

Figure 2. Markers were rigidly attached to the anterior surface of the thigh (A), and the upper leg positioned in an RF coil (B). These images show the first nine items investigated, as per the pictorial list shown in Figure 1.

Figure 3. Segmented volume reconstructions of the anterior thigh surface, showing an example of markers that were visible for each MRI sequence for the first acquisition of 9 markers as shown in Figure 2.

Figure 4. Workflow to measure relevant intensity parameters from each MRI dataset for each visible marker. A. 3D volume reconstruction of the T2, 3D sequence showing the transverse reslice plane (white line) and the range of z projection reslice plane (orange rectangle); B. Transverse reslice plane through the fish oil marker; C. Line selection across the marker (yellow unbroken line); D. Profile plot of intensity values along the line selection; E. Maximum intensity, transverse plane Z-projection of the fish oil capsule; F. Segmented selection tool demarcating the marker boundary (yellow broken line); G. Histogram detailing distribution of maximum intensities within the marker.

Figure 5: Reformatted transverse reslice images through the mid-height of all visible markers. Of the 18 markers, only 11 were visible on images from at least one of the MRI sequences – results for these 11 markers are shown.

Figure 6: Maximum and mean intensity for all visible markers. Results are shown for the five MRI sequences: Proton Density (PD), T2 weighted fat-suppressed (T2_FatSup), 3D T2 weighted (T2_3D), T1 weighted coronal (T1_coronal) and 3D T1 weighted (T1_3D).

Figure 7: Intensity ratios for all visible markers. Marker_Muscle and Marker_Fat are the ratios between maximum marker intensity and mean intensity in the fat tissue and muscle tissue, respectively. Results are shown for the five MRI sequences: Proton Density (PD), T2 weighted fat-suppressed (T2_FatSup), 3D T2 weighted (T2_3D), T1 weighted coronal (T1_coronal) and 3D T1 weighted (T1_3D)

For peer review only

Competing Interests & Funding Statement:

All authors have completed the ICMJE uniform disclosure form (available on request from the corresponding author) and declare: no support from any organisation for the submitted work; no financial relationships with any organisations that might have an interest in the submitted work in the previous three years; no other relationships or activities that could appear to have influenced the submitted work. No funding was received for this work.

Contributors:

MTI, CAG, JPL were involved in the conception, design and conduct of the research project, interpretation of the data and preparation of the manuscript. JPL performed the data analysis and edited the manuscript. DL wrote the first draft of the introduction and discussion. SM assisted with the design of the project, performed the imaging and assisted with the interpretation of the data and results. All authors were involved in the drafting and revising of the manuscript and gave final approval of the final version. All authors meet the ICMJE criteria for authorship. MTI and JPL are guarantors for the work and conduct of the study.

Transparency Declaration:

MTI and JPL affirm that the manuscript is an honest, accurate, and transparent account of the study being reported; that no important aspects of the study have been omitted; and that any discrepancies from the study as originally planned (and, if relevant) have been explained.

Ethical Approval:

Ethical approval was obtained from Queensland University of Technology Human Research Ethics Committee (approval # 1700000335)

Copyright:

The Corresponding Author has the right to grant on behalf of all authors and does grant on behalf of all authors, an exclusive licence on a worldwide basis to the BMJ Publishing Group Ltd to permit this article (if accepted) to be published in BMJ editions and any other BMJPGL products and sublicences such use and exploit all subsidiary rights, as set out in our licence.

Patient and Public Involvement:

This research was done without patient or public involvement as an aspect of a larger study yet to be performed. Participants in the larger study will be asked to be involved in the design of the subjective assessment methods.

Data Sharing:

The authors agree to consider sharing any data produced by this work.

For peer review only

References

1. Chan MF, Cohen GN, Deasy JO. Qualitative evaluation of fiducial markers for radiotherapy imaging. Technol Cancer Res Treat. 2015;14(3):298-304.

2. Schindel J, Muruganandham M, Pigge FC, et al. Magnetic resonance imaging (MRI) markers for MRI-guided high-dose-rate brachytherapy: novel marker-flange for cervical cancer and marker catheters for prostate cancer. Int J Radiat Oncol Biol Phys. 2013;86(2):387-93.

3. Scherman Rydhog J, Irming Jolck R, Andresen TL, et al. Quantification and comparison of visibility and image artifacts of a new liquid fiducial marker in a lung phantom for image-guided radiation therapy. Med Phys. 2015;42(6):2818-26.

4. Han BH, Wallner K, Merrick G, et al. The effect of interobserver differences in post-implant prostate CT image interpretation on dosimetric parameters. Med Phys. 2003;30(6):1096-102.

5. Roy JN, Wallner KE, Chiu-Tsao ST, et al. CT-based optimized planning for transperineal prostate implant with customized template. Int J Radiat Oncol Biol Phys. 1991;21(2):483-9.

6. Roy JN, Wallner KE, Harrington PJ, et al. A CT-based evaluation method for permanent implants: application to prostate. Int J Radiat Oncol Biol Phys. 1993;26(1):163-9.

7. Valente G, Pitto L, Testi D, et al. Are subject-specific musculoskeletal models robust to the uncertainties in parameter identification? PLoS ONE. 2014;9(11):e112625.

8. Freilich JM, Spiess PE, Biagioli MC, et al. Lipiodol as a fiducial marker for image-guided radiation therapy for bladder cancer. Int Braz J Urol. 2014;40(2):190-7.

9. Aras MH, Miloglu O, Barutcugil C, et al. Comparison of the sensitivity for detecting foreign bodies among conventional plain radiography, computed tomography and ultrasonography. Dentomaxillofac Radiol. 2010;39(2):72-8.

10. Kim SY, Park B, Kong IG, Choi HG. Analysis of ingested foreign bodies according to age, type and location: a retrospective observational study. Clin Otolaryngol. 2016;41(6):640-5.

11. Reddy N, Fata P, Balzer A, Diaz-Marchan P, Lincoln CM. Thorn in My Spine: A case of a retained intradural extramedullary foreign body. Clin Imaging. 2017;45:118-21.

12. Shelsta HN, Bilyk JR, Rubin PA, et al. Wooden intraorbital foreign body injuries: clinical characteristics and outcomes of 23 patients. Ophthalmic Plast Reconstr Surg. 2010;26(4):238-44.

13. Li J, Zhou LP, Jin J, Yuan HF. Clinical diagnosis and treatment of intraorbital wooden foreign bodies. Chin J Traumatol. 2016;19(6):322-5.

14. Shokri A, Jamalpour M, Jafariyeh B, et al. Comparison of Ultrasonography, Magnetic Resonance Imaging and Cone Beam Computed Tomography for Detection of Foreign Bodies in Maxillofacial Region. J Clin Diagn Res. 2017;11(4):TC15-TC9.

15. Pattamapaspong N, Srisuwan T, Sivasomboon C, et al. Accuracy of radiography, computed tomography and magnetic resonance imaging in diagnosing foreign bodies in the foot. Radiol Med. 2013;118(2):303-10.

16. McAllister AS, Lall NU, Tawfik KO, Jones BV. The imaging appearance of crayons. Pediatr Radiol. 2017;47(6):674-80.

17. Mundada P, Kohler R, Boudabbous S, Toutous Trellu L, Platon A, Becker M. Injectable facial fillers: imaging features, complications, and diagnostic pitfalls at MRI and PET CT. Insights Imaging. 2017;8(6):557-72.

For peer review only - http://bmjopen.bmj.com/site/about/guidelines.xhtml

18. Javadrashid R, Golamian M, Shahrzad M, et al. Visibility of Different Intraorbital Foreign Bodies Using Plain Radiography, Computed Tomography, Magnetic Resonance Imaging, and Cone-Beam Computed Tomography: An In Vitro Study. Can Assoc Radiol J. 2017;68(2):194-201.
19. Havard AC, Collins DJ, Guy RL, Husband JE. Magnetic resonance behaviour of lipiodol. Clin Radiol. 1992;45(3):198-200.
20. Gilbert JW, Wheeler GR, Richardson GB, et al. Guidance of magnetic resonance imaging and placement of skin-marker localization devices. J Neurosurg Sci. 2011;55(2):85-8.

For peer review only

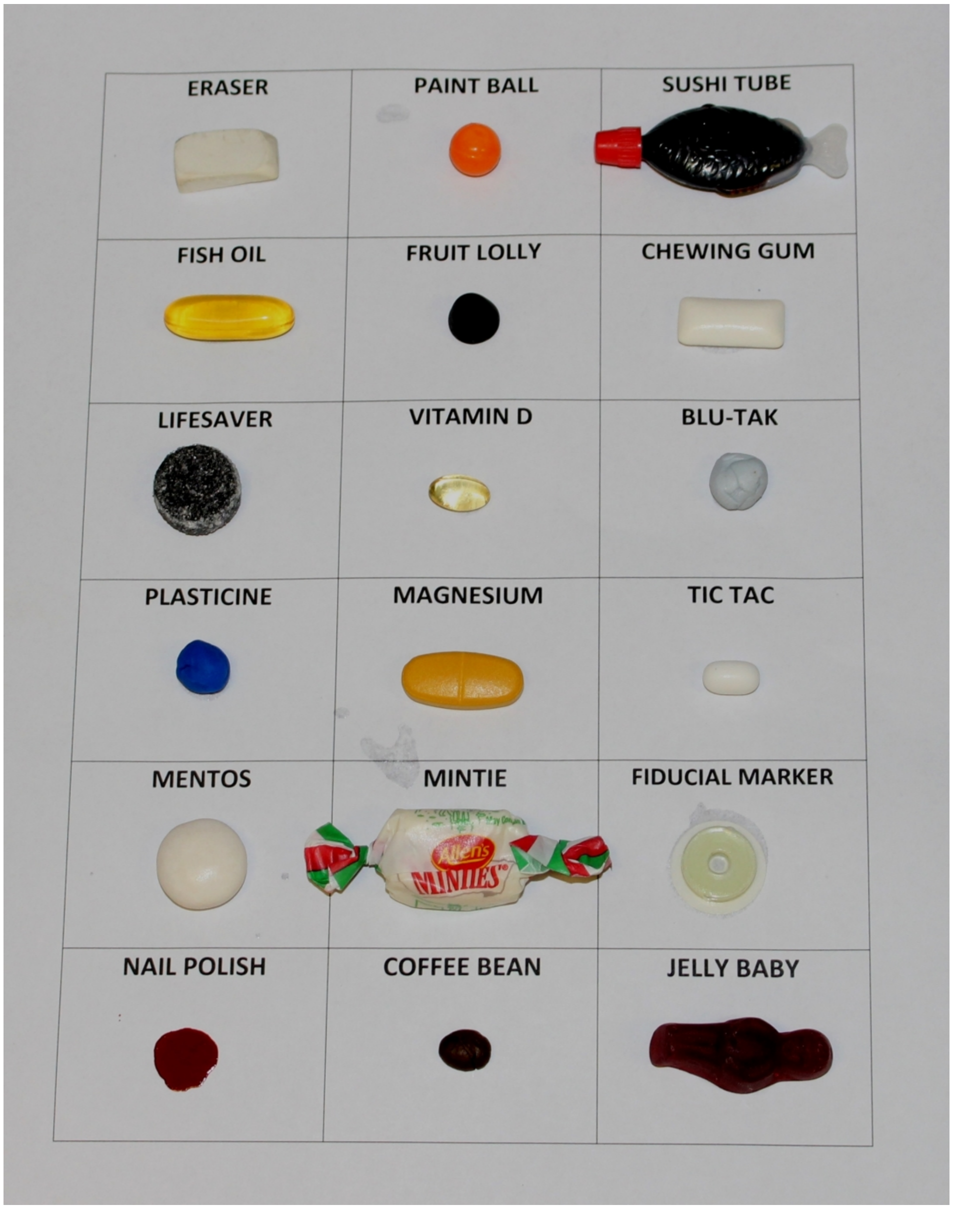

Figure 1. Items investigated as surrogates for the commercially available fiducial markers. Note that a commercial fiducial marker (IZI Multi-Modality Fiducial Marker, LifeHealthCare, Australia) was included as a benchmark for comparison.

818x1036mm (72 x 72 DPI)

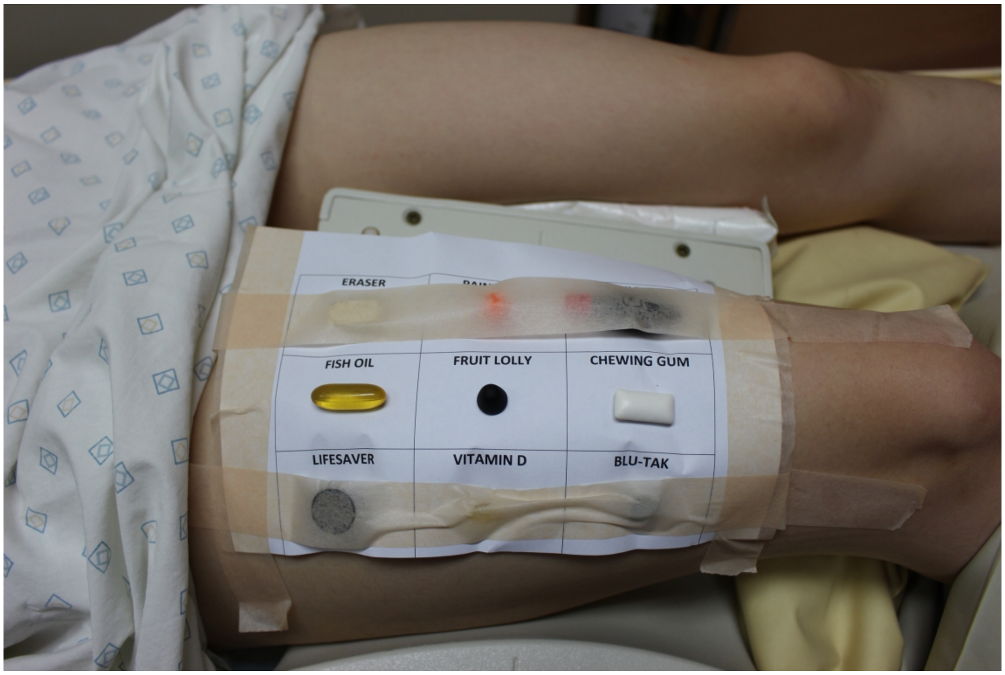

Figure 2. Marker candidates were rigidly attached to the anterior surface of the thigh. This image shows the first nine items investigated, as per the pictorial list shown in Figure 1.

1828x1219mm (72 x 72 DPI)

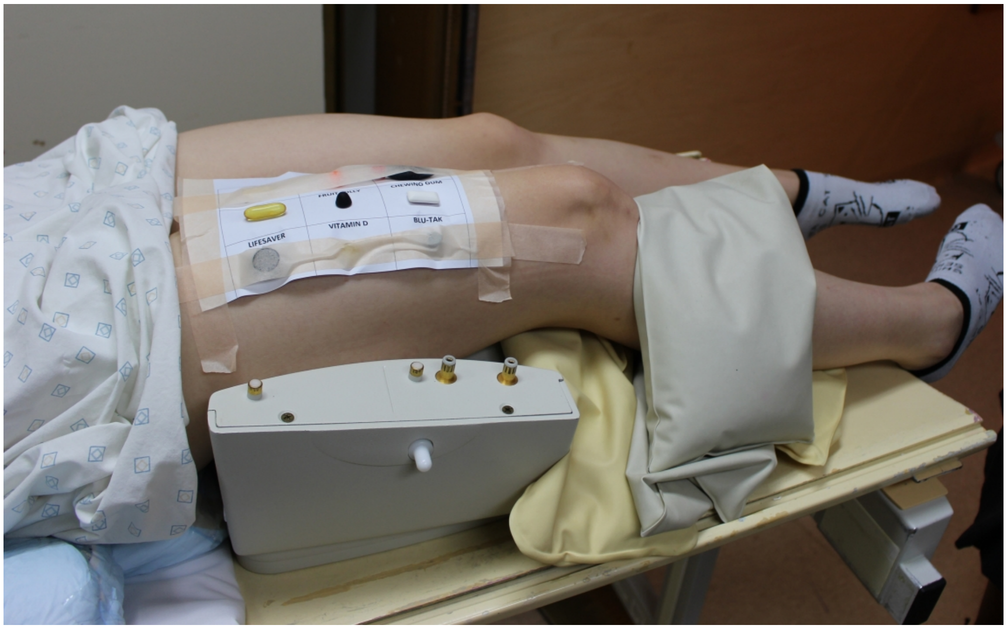

Figure 3. The thigh was positioned in an RF coil for the MRI scan. This image shows the first nine items investigated, as per the pictorial list shown in Figure 1.

1527x948mm (72 x 72 DPI)

<table>
<tr><td>Sequence</td><td>Proton Density</td><td>T1 Coronal</td><td>T1 3D</td><td>T2 Fat Sup</td><td>T2 3D</td></tr>
<tr><td>Segmented Volume View of Anterior Thigh Surface</td>
<td>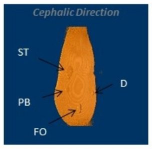
</td>
<td>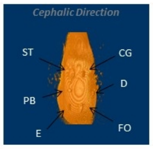
</td>
<td>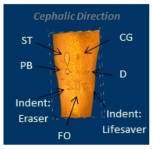
</td>
<td>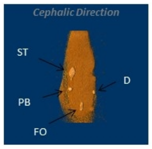
</td>
<td>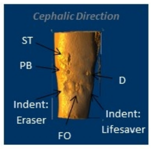
</td></tr>
<tr><td>Visible Markers</td>
<td>• Fish Oil (FO)
• Vitamin D (D)
• Paint Ball (PB)
• Sushi Tube (ST)</td>
<td>• Fish Oil (FO)
• Vitamin D (D)
• Paint Ball (PB)
• Sushi Tube (ST)
• Chewing Gum (CG)
• Eraser (E)
CG appeared to be visible on the 3D reconstruction but a 2D transverse slice at the level of CG did not demonstrate sufficient intensity contrast to differentiate the marker</td>
<td>• Fish Oil (FO)
• Vitamin D (D)
• Paint Ball (PB)
• Sushi Tube (ST)
• Chewing Gum (CG)
Indentations of the Lifesaver and Eraser were visible but the markers did not display</td>
<td>• Fish Oil (FO)
• Vitamin D (D)
• Paint Ball (PB)
• Sushi Tube (ST)</td>
<td>• Fish Oil (FO)
• Vitamin D (D)
• Paint Ball (PB)
• Sushi Tube (ST)
Indentation of the Lifesaver and Eraser are visible but marker does not display</td></tr>
</table>

Figure 4. Segmented volume reconstructions of the anterior thigh surface, showing an example of markers that were visible for each MRI sequence for the first acquisition of 9 markers as shown in Figure 2.

338x190mm (96 x 96 DPI)

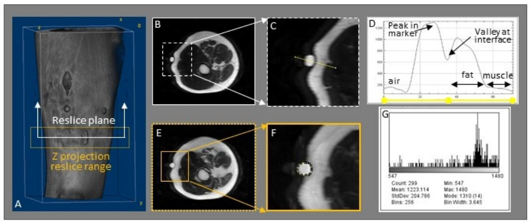

Figure 5. Workflow to measure relevant intensity parameters from each MRI dataset for each visible marker. A. 3D volume reconstruction of the T2, 3D sequence showing the transverse reslice plane (white line) and the range of z projection reslice plane (orange rectangle); B. Transverse reslice plane through the fish oil marker; C. Line selection across the marker (yellow unbroken line); D. Profile plot of intensity values along the line selection; E. Maximum intensity, transverse plane Z-projection of the fish oil capsule; F. Segmented selection tool demarcating the marker boundary (yellow broken line); G. Histogram detailing distribution of maximum intensities within the marker.

338x190mm (96 x 96 DPI)

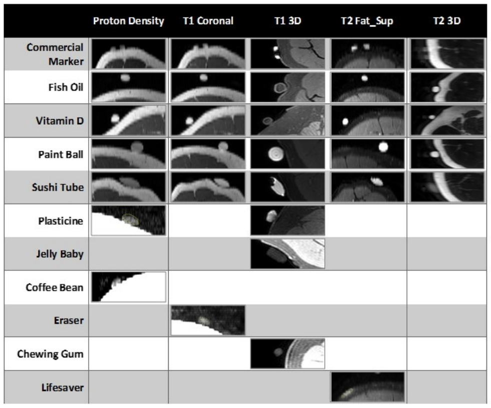

Figure 6: Reformatted transverse reslice images through the mid-height of all visible markers. Of the 18 markers, only 11 were visible on images from at least one of the MRI sequences – results for these 11 markers are shown.

338x190mm (96 x 96 DPI)

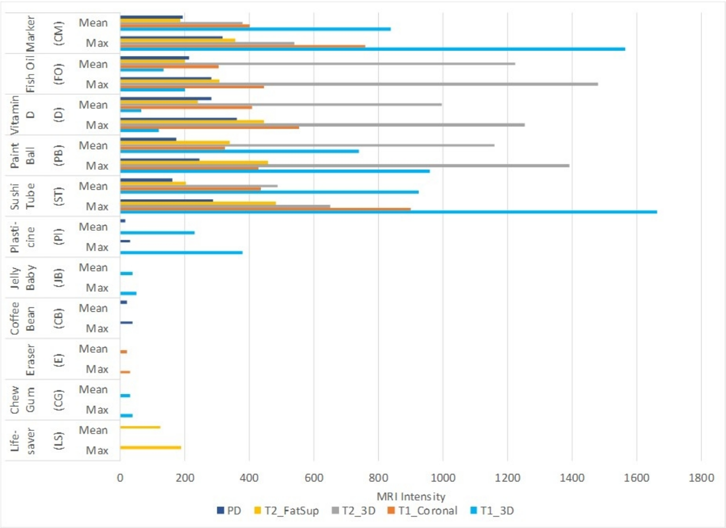

Figure 7: Maximum and mean intensity for all visible markers. Results are shown for the five MRI sequences: Proton Density (PD), T2 weighted fat-suppressed (T2_FatSup), 3D T2 weighted (T2_3D), T1 weighted coronal (T1_coronal) and 3D T1 weighted (T1_3D).

338x190mm (96 x 96 DPI)

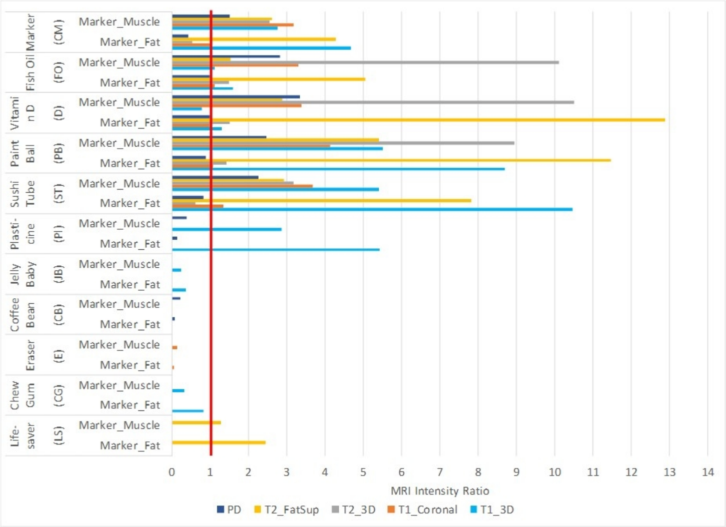

Figure 8: Intensity ratios for all visible markers. Marker_Muscle and Marker_Fat are the ratios between maximum marker intensity and mean intensity in the fat tissue and muscle tissue, respectively. Results are shown for the five MRI sequences: Proton Density (PD), T2 weighted fat-suppressed (T2_FatSup), 3D T2 weighted (T2_3D), T1 weighted coronal (T1_coronal) and 3D T1 weighted (T1_3D)

338x190mm (96 x 96 DPI)

Section & Topic	No	Item	Reported on page #
TITLE OR ABSTRACT		**Reliably Visible and Inexpensive surface Fiducial Marker options for Magnetic Resonance Imaging**	1
	1	Identification as a study of diagnostic accuracy using at least one measure of accuracy (such as sensitivity, specificity, predictive values, or AUC)	4
ABSTRACT			
	2	Structured summary of study design, methods, results, and conclusions (for specific guidance, see STARD for Abstracts)	2
INTRODUCTION			
	3	Scientific and clinical background, including the intended use and clinical role of the index test	3-4
	4	Study objectives and hypotheses	3-4
METHODS			
Study design	5	Whether data collection was planned before the index test and reference standard were performed (prospective study) or after (retrospective study)	Not applicable
Participants	6	Eligibility criteria	Not applicable
	7	On what basis potentially eligible participants were identified (such as symptoms, results from previous tests, inclusion in registry)	Not applicable
	8	Where and when potentially eligible participants were identified (setting, location and dates)	Not applicable
	9	Whether participants formed a consecutive, random or convenience series	Not applicable
Test methods	10a	Index test, in sufficient detail to allow replication	4-10
	10b	Reference standard, in sufficient detail to allow replication	4-10
	11	Rationale for choosing the reference standard (if alternatives exist)	4-10
	12a	Definition of and rationale for test positivity cut-offs or result categories of the index test, distinguishing pre-specified from exploratory	7-11
	12b	Definition of and rationale for test positivity cut-offs or result categories of the reference standard, distinguishing pre-specified from exploratory	7-11
	13a	Whether clinical information and reference standard results were available to the performers/readers of the index test	Not applicable
	13b	Whether clinical information and index test results were available to the assessors of the reference standard	Not applicable
Analysis	14	Methods for estimating or comparing measures of diagnostic accuracy	4-10
	15	How indeterminate index test or reference standard results were handled	Not applicable
	16	How missing data on the index test and reference standard were handled	Not applicable
	17	Any analyses of variability in diagnostic accuracy, distinguishing pre-specified from exploratory	Not applicable
	18	Intended sample size and how it was determined	Not applicable
RESULTS			
Participants	19	Flow of participants, using a diagram	Not applicable
	20	Baseline demographic and clinical characteristics of participants	Not applicable
	21a	Distribution of severity of disease in those with the target condition	Not applicable
	21b	Distribution of alternative diagnoses in those without the target condition	Not applicable
	22	Time interval and any clinical interventions between index test and reference standard	Not applicable
Test results	23	Cross tabulation of the index test results (or their distribution) by the results of the reference standard	11-16
	24	Estimates of diagnostic accuracy and their precision (such as 95% confidence intervals)	Not applicable
	25	Any adverse events from performing the index test or the reference standard	Not applicable
DISCUSSION			
	26	Study limitations, including sources of potential bias, statistical uncertainty, and generalisability	18
	27	Implications for practice, including the intended use and clinical role of the index test	15-17
OTHER INFORMATION			
	28	Registration number and name of registry	Not applicable
	29	Where the full study protocol can be accessed	4-10
	30	Sources of funding and other support; role of funders	17

For peer review only

CC BY SA

STARD 2015

AIM

STARD stands for "Standards for Reporting Diagnostic accuracy studies". This list of items was developed to contribute to the completeness and transparency of reporting of diagnostic accuracy studies. Authors can use the list to write informative study reports. Editors and peer-reviewers can use it to evaluate whether the information has been included in manuscripts submitted for publication.

EXPLANATION

A **diagnostic accuracy study** evaluates the ability of one or more medical tests to correctly classify study participants as having a **target condition.** This can be a disease, a disease stage, response or benefit from therapy, or an event or condition in the future. A medical test can be an imaging procedure, a laboratory test, elements from history and physical examination, a combination of these, or any other method for collecting information about the current health status of a patient.

The test whose accuracy is evaluated is called **index test.** A study can evaluate the accuracy of one or more index tests. Evaluating the ability of a medical test to correctly classify patients is typically done by comparing the distribution of the index test results with those of the **reference standard**. The reference standard is the best available method for establishing the presence or absence of the target condition. An accuracy study can rely on one or more reference standards.

If test results are categorized as either positive or negative, the cross tabulation of the index test results against those of the reference standard can be used to estimate the **sensitivity** of the index test (the proportion of participants *with* the target condition who have a positive index test), and its **specificity** (the proportion *without* the target condition who have a negative index test). From this cross tabulation (sometimes referred to as the contingency or "2x2" table), several other accuracy statistics can be estimated, such as the positive and negative **predictive values** of the test. Confidence intervals around estimates of accuracy can then be calculated to quantify the statistical **precision** of the measurements.

If the index test results can take more than two values, categorization of test results as positive or negative requires a **test positivity cut-off**. When multiple such cut-offs can be defined, authors can report a receiver operating characteristic (ROC) curve which graphically represents the combination of sensitivity and specificity for each possible test positivity cut-off. The **area under the ROC curve** informs in a single numerical value about the overall diagnostic accuracy of the index test.

The **intended use** of a medical test can be diagnosis, screening, staging, monitoring, surveillance, prediction or prognosis. The **clinical role** of a test explains its position relative to existing tests in the clinical pathway. A replacement test, for example, replaces an existing test. A triage test is used before an existing test; an add-on test is used after an existing test.

Besides diagnostic accuracy, several other outcomes and statistics may be relevant in the evaluation of medical tests. Medical tests can also be used to classify patients for purposes other than diagnosis, such as staging or prognosis. The STARD list was not explicitly developed for these other outcomes, statistics, and study types, although most STARD items would still apply.

DEVELOPMENT

This STARD list was released in 2015. The 30 items were identified by an international expert group of methodologists, researchers, and editors. The guiding principle in the development of STARD was to select items that, when reported, would help readers to judge the potential for bias in the study, to appraise the applicability of the study findings and the validity of conclusions and recommendations. The list represents an update of the first version, which was published in 2003.

More information can be found on http://www.equator-network.org/reporting-guidelines/stard.

For peer review only

cc BY SA

BMJ Open

Determining a Reliably Visible and Inexpensive Surface Fiducial Marker for Use in Magnetic Resonance Imaging: a Research study in a busy public radiology department.

Journal:	*BMJ Open*
Manuscript ID	bmjopen-2018-027020.R1
Article Type:	Research
Date Submitted by the Author:	01-May-2019
Complete List of Authors:	Izatt, Maree; Queensland University of Technology, Biomechanics & Spine Research Group<br>Lees, Deborah; Queensland University of Technology, Biomechanics & Spine Research Group<br>Mills, Susan; Mater Misericordiae Brisbane Ltd, Mater Medical Imaging<br>Grant, Caroline; Queensland University of Technology, Biomechanics & Spine Research Group<br>Little, Judith; Queensland University of Technology, Biomechanics & Spine Research Group
**Primary Subject Heading**:	Qualitative research
Secondary Subject Heading:	Diagnostics, Paediatrics, Patient-centred medicine, Public health, Radiology and imaging
Keywords:	Magnetic resonance imaging < RADIOLOGY & IMAGING, MRI, fiducial marker, surface marker, MRI sequence, marker visibility

SCHOLARONE™
Manuscripts

Title:

Determining a Reliably Visible and Inexpensive Surface Fiducial Marker for Use in Magnetic Resonance Imaging: a Research study in a busy public radiology department.

Authors:

Maree T. Izatt[1], B.Phty m.izatt@qut.edu.au
Project Coordinator, (ORCID ID: 0000-0002-4926-3077)

Deborah Lees[1], M.Tech (Chiro), MBBS, FRCS (Trauma & Orth) deborah.lees@health.qld.gov.au
Spine Fellow, Orthopaedic Surgeon

Susan Mills[2], B.App.Sci (MI) susan.mills@mater.org.au
MRI Unit Manager

Caroline A. Grant[1], PhD, BEng ca.grant@qut.edu.au
Postdoctoral Research Fellow, (ORCID ID: 0000-0002-5128-9903)

J. Paige Little[1], PhD, BEng j2.little@qut.edu.au
Senior Research Fellow, (ORCID ID: 0000-0001-8377-5343)

Affiliations:

[1] Biomechanics and Spine Research Group, Institute of Health and Biomedical Innovation at Centre for Children's Health Research, Queensland University of Technology, Brisbane, Australia

[2] Mater Medical Imaging, Mater Misericordiae Ltd, Brisbane, Australia

Corresponding Author:

Dr J P Little Email: j2.little@qut.edu.au; Phone: +61 7 3069 7325

For peer review only - http://bmjopen.bmj.com/site/about/guidelines.xhtml

Summary Box

- A reliably visible and inexpensive surface fiducial marker for MRI was required as an alternative to the single-use commercial markers currently available.
- A search of the literature did not provide any alternative so the current study sought to find a cheap, easily sourced alternative marker for use in clinical imaging departments or research projects utilising MRI.
- Four items were seen reliably on the five most commonly requested MRI sequences; fish oil capsule, vitamin D capsule, paint ball pellet and sushi soy sauce tube.
- The Vitamin D capsule proved to be ideal, with an excellent balance between availability, size, cost, and quality of the visualised marker for commonly used MRI sequences.
- Clinical MRI departments and researchers requiring the use of surface fiducial markers now have cheap alternative marker options to use in place of the relatively expensive single-use commercial fiducial marker.

For peer review only

Structured Abstract

Objectives

Single use commercial surface fiducial markers are used in clinical imaging for a variety of applications. The current study sought to find a new, reliably visible, easily sourced and inexpensive fiducial marker alternative for use with magnetic resonance imaging (MRI).

Design

Five commonly requested MRI sequences were determined (3D T1-weighted, T1 coronal, 3D T2-weighted, T2 fat suppressed, Proton density), to examine the visibility of 18 items (including a commercial fiducial marker).

Setting

Clinical 3T MRI scanner in Tertiary Public Hospital and University Biomedical Engineering research group

Interventions

18 marker alternatives were scanned using five common MRI sequences. Images were reformatted to obtain both an image through the mid-height of each marker and a maximum intensity z-projection image over the volume of the marker. Variations in marker intensity were profiled across each visible marker and a visibility rating defined.

Main outcome measures

Outcome measures were based on quantitative assessment of a clear intensity contrast ratio between the marker and the adjacent tissue and a qualitative assessment of visibility via a 3-point scale.

For peer review only - http://bmjopen.bmj.com/site/about/guidelines.xhtml

Results
The fish oil capsule, vitamin D capsule, paint ball pellet, soy sauce sushi tube and commercial markers were typically visible to a high quality on all the imaging sequences and demonstrated a clear differential in intensity contrast against the adjacent tissue. Other common items, such as plasticine 'play doh' and a soft 'Jelly baby' sweet were surprise candidates, demonstrating high quality visibility and intensity contrast for the 3D T1-weighted sequence.

Conclusions
Depending on the basis for referral and MRI sequence chosen, four alternative fiducial markers were determined to be inexpensive, easily sourced and consistently visible. Of these, the Vitamin D capsule provided an excellent balance between availability, size, cost, usability and quality of the visualised marker for all the commonly used MRI sequences analysed.

For peer review only

Strengths and Limitations

- This manuscript is the first to test for easily sourced and economical items to find reliably visible surface fiducial marker options for common MRI sequences
- The anterior surface of a healthy female thigh was used to scan the seventeen different items in a 3T MRI scanner as this was considered superior to using a saline phantom object
- Both a qualitative analysis of visibility assessed the practical ability of the radiographer/investigator to visualise the marker.
- A quantitative analysis of intensity contrast to adjacent tissues were performed in comparison to a commercial surface fiducial marker (gold standard) to critically analyse the reliability of the marker alternatives.
- After surveying local surgeons and radiographers, five common MRI sequences were used to provide the most relevant alternative marker for MR clinical imaging.

Keywords
Magnetic resonance imaging, MRI, fiducial marker, surface marker, MRI sequence, marker visibility, marker intensity, low cost marker, inexpensive marker

For peer review only - http://bmjopen.bmj.com/site/about/guidelines.xhtml

Introduction:

Fiducial markers used in the context of clinical medicine may be implanted in the body or placed on the surface and are also utilised in research for a variety of applications. Placed in the field of view, they display as distinct regions of high intensity to assist in pinpointing specific anatomical landmarks or pathologies on the acquired clinical images. Surface markers placed on a patient's skin can also provide a frame of reference for registration of medical images acquired using multiple imaging modalities, such as photography, computed tomography (CT) and magnetic resonance imaging (MRI) performed for concomitant pathologies involving multiple specialities.(1)

In radiotherapy, fiducial markers are increasingly being used to identify the tumour site, permitting image registration and assisting with guidance for treatment planning.(2-4) Oil-based surface markers have been shown to compare favourably to solid markers in terms of their contrast to noise ratio, resulting in excellent visibility.(4) Prostate marker studies also revealed that implanted titanium seeds left star/streak artefacts on CT imaging and could not be accurately localised on MR due to negative contrast (black holes).(5-7) Fiducial markers also perform a valuable role in guiding anatomical identification in musculoskeletal studies, particularly kinematic gait analyses where the location of surface markers on both mathematical gait models and the accompanying 3D MRI images provides valuable subject-specific simulation parameters.(8)

Commercially available fiducial surface markers (i.e. non-implantable) are available in a range of sizes and shapes for use in the wide variety of clinical imaging applications. They are self-adhesive and have a low, flat profile, making them comfortable for the patient. While these markers provide excellent utility, they present a substantial expense to radiology departments, with a review of the current markets showing prices ranging from $420 to $600 USD for a box of 100 (MR Spots® and IZI multi-modality, excluding shipping and tax). Being a single use product they are a considerable expense to the running costs of imaging departments or research teams utilizing MRI and requiring numerous markers.

A review of the literature to find materials that are visible in MRI revealed a paucity of publications. The majority of papers examining substance visibility were focused primarily on locating a variety of penetrating or ingested foreign bodies and just a few papers explored implanted marker options for tumour boundary markers prior to excision or radiotherapy planning.(3, 5-7, 9) Foreign bodies that had been located, ranged in material from fish and chicken bones, batteries, plastics, coins, metal and wood splinters (10-13) and it should be noted that some materials were missed even with the high sensitivity of modern MRI and CT.(14, 15) Pattamapospong *et al* (16) published results regarding the visibility of a number of substances on X-ray, CT and MRI and demonstrated a 100% specificity but only 58% sensitivity for fresh wood, dry wood, glass, plastic and porcelain using MRI. Wax crayons have proven to be easily visible on both CT and MRI with high attenuation noted on CT and a signal void on both T1 and T2 MR images. Interestingly, it was noted that crayon colour influenced the degree of visibility. Different pigments used in crayons and in paints resulted in distinctive colours as well as MRI appearance.(17) Injectable facial fillers (hyaluronic acid, collagen and polyalkylimide-polyacrylamide hydrogels), silicone and calcium hydroxyapatite have also been well visualised on MRI but can be confused with malignant features, so are not considered good marker substances.(18) Metal is well visualised on many modalities (10) but due to its ferrous properties, is contra-indicated in the field of MRI. Metal, even if MR conditional, results in significant image attenuation and artefact. For these reasons, an iron tablet as a makeshift marker may be well

For peer review only - http://bmjopen.bmj.com/site/about/guidelines.xhtml

visualised but the resulting artefact renders it unacceptable. Studies looking at the visibility of plastic, stone, glass and graphite revealed that both glass and plastic foreign bodies are not consistently visible on MRI or plain radiographs; despite showing up well on CT.(16, 19)

A review of the physics of MRI suggested materials with high water or fat content were likely items that would be readily detectable. Fiducial markers should be easily identifiable and clearly visible on clinical images, allowing the target anatomy or volume to be clearly and completely visualised. Therefore a critical objective of this study was to assess the validity of various ‘everyday’ items, which could be easily and economically sourced, and provide a reliably visible fiducial marker as an alternative to the comparatively expensive, single use commercial fiducial markers.

Methods:

Seventeen everyday items and a commercial fiducial marker were selected for analysis, either from the literature or anecdotal reports (Figure 1). The important considerations relating to the selection of a suitable surrogate for commercial fiducial markers were identified and are outlined in Table 1. In addition to the commercial fiducial marker, two of these items had been used in our local hospital medical imaging department; the paint ball for MR scans in a pilot research project, and the fish oil capsule which was in regular use to avoid using a commercial marker whenever possible. While these two non-commercial markers provided excellent results in terms of intensity contrast and visualisation on MR images, to date a definitive comparison of the image contrast provided by these items has not been undertaken and these alternatives did not meet all the desired requirements of the ideal alternative fiducial marker.

Table 1. Important considerations relating to the choice of a suitable fiducial marker for MR imaging

**Factors for consideration**	
**Accessibility**	Easily acquired, readily replaced, and preferably locally sourced without high shipment costs or transit time
**Use**	Sufficiently robust to tolerate body weight if placed between the participant and scanner bed<br>Suitably compliant or flat to ensure minimal discomfort to the patient if marker is in a load bearing location (e.g. Posterior superior iliac spine for a supine MRI)
**Biochemistry**	Chemically inert, hypoallergenic
**Economy**	Low acquisition cost per marker given markers are single use items
**Medical Physics**	Sufficient hydrogen content to permit high intensity contrast to surrounding tissue
**Fixation**	Reliably affixed to the patient’s external anatomy

For peer review only - http://bmjopen.bmj.com/site/about/guidelines.xhtml

Imaging Details:

Table 2. MRI sequence details

	Imaging Protocol	Acquisition plane	Field of View (FOV) (mm)	Repetition time (TR) / Echo time (TE) (msec)	Resolution (Pixel width x height x slice spacing, mm)
**1**	Proton density (PD)	Coronal	300 x 300 x 44	2,435/35	0.1465 x 0.1465 x 2.2
**2**	T1 weighted, Spin echo	Coronal	300 x 300 x 44	661/10	0.1465 x 0.1465 x 2.2
**3**	T1 weighted, 3D Fast field echo (FFE)	Sagittal	300 x 300 x 175	25/1.4	0.469 x 0.469 x 0.5
**4**	T2 weighted, fat suppressed	Coronal	300 x 300 x 44	4,448/65	0.195 x 0.195 x 2.2
**5**	T2 weighted, 3D	Sagittal	300 x 300 x 175	2,473/72	0.469 x 0.469 x 0.5

The selection of items underwent MR scanning in a clinical medical imaging department using a 3-T Philips Achieva MRI scanner. Scanning parameters vary between different machine manufacturers and even between different scanner models from the same manufacturer. However, this particular scanner was selected as it services a busy hospital radiology department in a metropolitan city, and thus, provides a broad range of exploratory and treatment-based MRI scanning services. Further to this, the 3-T magnet strength is a typical specification for scanners in tertiary care facilities in Australia and internationally.

Following the advice from the senior MR radiographers and a survey of local surgeons, five different sequences were performed (Table 2) to cover the breadth of MR imaging studies typically performed with a requirement for inclusion of surface markers. All tested markers were attached to the anterior thigh of a healthy female participant, aged 27 years, who had provided informed consent. A radiofrequency coil (8-channel knee coil) was positioned beneath the thigh and the markers attached to the anterior thigh surface (Figure 1). Due to physical size constraints, the marker selection was attached to the volunteer's leg in two separate acquisitions. In all the MR sequences detailed in Table 2, the scanning parameters were constant for both acquisitions.

Images were saved in Digital Imaging and Communications in Medicine (DICOM) format and analysed using ImageJ (U. S. National Institute of Health Open-source software, Maryland, USA; https://imagej.nih.gov/ij/). A similar process was utilised to assess the results from each of the five different imaging sequences (Table 2) and involved first viewing the 3D stack as a segmented volume using the ImageJ Plugin, 'Volume Viewer 2.01' (K U Barthel, Internationale Medieninformatik, HTW Berlin, Germany) (Figure 3).

For peer review only - http://bmjopen.bmj.com/site/about/guidelines.xhtml

In the first instance, a qualitative evaluation of the marker visibility was carried out to subjectively assess the relative ease with which each marker could be visualised and the marker edges demarcated. Viewing the data as a 3D volume permitted an initial assessment of which markers exhibited an intensity that was visible on MR images (Figure 3). If the marker was visible, the visibility was rated on a 3-point Likert scale with 3 representing 'very clear', 2 representing 'edges visible but fuzzy' and 1 representing 'not easily visible'. If a visibility rating of one was observed, this low rating suggested that while the marker was discernibly visible on the MR images, the quality of visibility was poor and the marker would not be recommended for use in clinical scanning using that particular MR sequence.

In all cases, MR images were reformatted and processed as 16-bit images, with an intensity range from 0 to 65,536. A quantitative evaluation of the marker quality was carried out to assess the visibility, artefacts and distortion of marker boundaries created by each item on each MRI sequence. This evaluation was carried out using the steps outlined below and shown visually in Figure 4.

1. Reformatted Image Stack: The DICOM stacks were reformatted to create a sequential stack of transverse reslice images, with consideration of the relevant pixel and slice spacing for each dataset (Table 2).
2. Intensity Profile Plot: Using the 3D volume reconstructions for each MRI sequence as a reference (Figure 3, Figure 4 A), the reformatted transverse reslice through the mid-height of each marker was located (Figure 4 B). A line selection (Figure 4 C) was drawn through the marker, such that the mid-point on the line was positioned at the interface between the marker and the skin and the endpoints were located in air and muscle tissue, respectively. The intensity profile along this line selection (Figure 4 D) was used to objectively compare the marker signal and visibility. The peak intensity in the marker, minimum intensity at the interface between the marker and tissue, mean intensity in the fat tissue and mean intensity in the muscle tissue were recorded from the profile.
3. Maximum Intensity Z-projection: A two-dimensional (2D) image with each pixel intensity equivalent to the maximum intensity along a Z trajectory over a given range of stack images was created (Figure 4 E) The image range was defined to encompass the upper and lower limits of the marker, as viewed on the 3D volume reconstruction (Figure 4 A, orange broken line).
4. Intensity Histogram and Maximum Marker Intensity: A segmented line tool (Figure 4 F) was used to demarcate the outer profile of the marker on the 2D Z-projection image (Figure 4 E). A histogram showing the distribution of maximum intensities within this profile for the marker was used to record the maximum and mean marker intensity (Figure 4 G).

In some instances (Figure 3: T1_3D – Chewing Gum; T1 Coronal – Eraser) the intensity range exhibited by the marker was very low in comparison to the background intensity, making the marker boundaries difficult to differentiate from the surrounding regions. However, these markers were still included in the analysis as it was possible to create a profile plot (Figure 4 D) on a transverse plane through the mid-height of the marker, with a measurable intensity gradient across the marker.

Assessment of Intensity Values:

In order to objectively assess whether the markers provided an acceptable visualisation on the images produced for each MR sequence analysed, three criteria were defined to assess the results. It was expected that the markers should; a) Provide a pixel intensity that was sufficiently above the background intensity of air to result in a clear visual contrast; b) Demonstrate a pixel intensity ratio at the interface between the marker and adjacent epidermis/fat tissue (i.e. peak marker intensity

For peer review only - http://bmjopen.bmj.com/site/about/guidelines.xhtml

divided by interface intensity/mean fat tissue intensity) greater than 1; and c) Demonstrate a pixel intensity ratio between the marker and the muscle tissue (i.e. peak marker intensity divided by mean muscle intensity) greater than 1.

The ratios were intended to provide a numeric value that aligned with the perceptual and visual decision making process a person undertakes when determining whether a marker is visualised at an acceptable quality. To indicate the quality of marker visualisation, this ratio of unity was defined after viewing all markers over the five MRI sequences and in combination with the visual assessment of visibility rating.

Depending on the MRI sequence, the fat and muscle tissue adjacent to the marker displayed over different intensity ranges. The ratio between the fat or muscle tissue and marker intensity was considered critical to be able to differentiate the marker from its surroundings. For this reason the intensity ratio was considered of more importance than the absolute value of the pixel intensity.

Patient and Public Involvement:

This research was done without patient or public involvement as an aspect of a larger study yet to be performed. Participants in the larger study will be asked to be involved in the design of the subjective assessment methods.

Results

Of the 18 markers investigated, five were consistently visible for all imaging sequences, but this visibility was of differing quality (**Error! Reference source not found.**). The Fish Oil (FO), Vitamin D (D), Paintball (PB), Soy Sauce Sushi Tube (ST) and Commercial Marker (CM) were typically visible to a high quality (i.e. rating 2 or 3) for all sequences (**Error! Reference source not found.**). A visibility rating of 1 on only one of the MR sequences was observed for the Eraser (E), Chewing Gum (CG), Lifesaver (LS), and Coffee Bean (CB), demonstrating that while these markers were visible, the quality of visibility was poor. Surprisingly, the Pl marker was visible to a high quality (rating 3) for the T1_3D sequence, but had little (rating = 1 for PD sequence) or no visibility for the other sequences. Similarly, the Jelly Baby was also remarkable in being well visible on the T1_3D sequence with a rating of 2, but had no visibility scored for the other MR sequences.

While a mean visibility rating >0 indicated that the marker could be seen on at least one of the MRI sequences, a mean visibility rating greater than 2 was preferable as it indicated a consistently good quality visibility on all of the MRI sequences performed. A mean visibility score greater than 2 was observed for the PB, FO, D and CM markers (**Error! Reference source not found.**).

Table 3. Visibility rating for each marker on each MRI sequence. If not visible, the marker rating = 0. If visible, marker visibility rating was either 1 ('visible, but not easily'), 2 ('edges visible but fuzzy') or 3 ('very clear').

For peer review only - http://bmjopen.bmj.com/site/about/guidelines.xhtml

	Proton Density	T1 Coronal	T1_3D	T2 Fat_Sup	T2_3D	MEAN RATING
**Commercial Marker**	2	2	3	3	2	2.4
**Fish Oil**	2	2	3	2	3	2.4
**Vitamin D**	2	2	3	3	3	2.6
**Paint Ball**	2	2	3	3	3	2.6
**Soy Sauce Sushi Tube**	2	2	3	2	1	2.0
**Plasticine**	1	0	3	0	0	0.8
**Jelly Baby**	0	0	2	0	0	0.4
**Coffee Bean**	1	0	0	0	0	0.2
**Eraser**	0	1	0	0	0	0.2
**Chewing Gum**	0	0	1	0	0	0.2
**Lifesaver**	0	0	0	1	0	0.2
**Fruit Lolly**	0	0	0	0	0	0
**BluTak**	0	0	0	0	0	0
**Magnesium**	0	0	0	0	0	0
**TicTac**	0	0	0	0	0	0
**Mentos**	0	0	0	0	0	0
**Mintie**	0	0	0	0	0	0
**Nail Polish**	0	0	0	0	0	0

Figure 5 shows a summary of the visible markers on the five MRI sequences. In each case, the intensity range for the image was selected such that the intensity contrast within the image could best display the marker. Note however, these images do not include image filtering or contrast enhancement. Viewing the reslice images in Figure 5 in combination with the visibility ratings in Table 3 demonstrated that the best quality marker visibility for the T1_3D, T1 coronal, T2 Fat_Sup and Proton Density sequences was the PB, ST, FO and D; and for the T2_3D sequence was the PB, FO and D. The CM was best visualised on the T1_3D and T2 Fat_Sup sequences.

Intensity ratios between the maximum marker intensity and the mean intensity in either the fat tissue or muscle tissue are shown in Figure 6.

For the PD sequence, while the CM, FO, D, PB and ST markers were easily visible (Figure 5, Figure 6), the marker intensity was numerically similar to the fat tissue, resulting in marker-to-fat ratios near or below one. The PL and CB were less easily differentiated from the nearby fat tissue (Figure 6).

For peer review only - http://bmjopen.bmj.com/site/about/guidelines.xhtml

For the T2 fat suppressed sequence, there was a distinct differential between the maximum intensity in the marker and the mean intensity in the fat tissue, making all observable markers (CM, FO, D, PB, ST and LS) clearly visible (Figure 6).

The commercial marker (CM) was not clearly visualised for the 3D T2-weighted sequence (Table 3, Figure 5), and had a marker-to-fat ratio <1 (Figure 6). While the ST tended to result in good visibility (Table 3, Figure 5), for the 3D T2-weighted sequence the marker intensity was less than the muscle tissue and not easily differentiated from the background air intensity. Aside from this, the 3D T2-weighted sequence showed an intensity ratio >>1 and thus clear intensity contrast for the marker-to-muscle ratio (>1) and marker-to-fat ratio, for all markers.

While the eraser (E) was visible on the T1 coronal sequence, the intensity ratios suggested that the intensity contrast between the marker and adjacent muscle/fat was not of a good quality (Table 3, Figure 5). This was supported by the observed intensity ratio <1 for both the marker-to-muscle and marker-to-fat ratios (Figure 6). For the other visible markers, the intensity ratios were all ≥ 1.
With the exception of the chewing gum (CG) and jelly baby (JB), the intensity ratios for marker-to-fat were all >1, for the 3D_T1-weighted sequence. In the case of the PB and ST marker, since these were comprised of a non-oil based substance, the intensity contrast and marker-to-fat/marker-to-muscle intensity ratios were >>1 (Figure 6). These latter two markers also resulted in a high visibility rating (Table 3, Figure 5).

Discussion

Fiducial markers are used for calibration of the imaging equipment, providing a reference measurement for templating software, locating specific boundaries for radiotherapy planning, orienting an image and very importantly, to identify a specific area of interest in the case of diagnostic queries. It is thus essential to have a marker that is detectable and clearly discernible from bone and soft tissues when placed in the visual field for a number of MR sequences. Single use, commercially available fiducial markers make up a significant component of the imaging department’s outgoings and in an attempt to reduce costs, the current study was a practical exercise designed to image a number of commonly available everyday items to assess their suitability to function as an effective surface fiducial MRI marker substitute.

The current study also aimed to provide additional marker options useful for personalised musculoskeletal kinematic models as well as for clinical pathologies and research projects utilizing MRI.

A single use, commercially available surface fiducial marker was evaluated in this study alongside seventeen potential alternatives and its performance was surprisingly less impressive than some of the more common and affordable items tested. The commercial marker is considered prohibitively expensive (AUD$ 6 to 10 per marker) and is therefore used selectively for specific neurosurgical imaging needs only. Makeshift markers of various types have been anecdotally trialled in clinical radiology departments over the years. Discussions with our hospital imaging staff revealed that almonds, Vitamin E capsules and condiment packets had previously been trialled. Packaging rupture was a risk and unfortunately these makeshift markers caused issues with patient comfort, and inconsistent visibility on imaging.

For peer review only - http://bmjopen.bmj.com/site/about/guidelines.xhtml

Fluids are more readily identifiable on MRI and lipid-based markers have proven to be consistently reliable.(9, 20) The capped sushi soy sauce tubes tested in the current study were reliably visible on all the MRI sequences analysed and were considered by the MR radiographers as a useful alternative marker candidate. They suggested one or more of the fish-shaped tubes could act as a type of "pointer" to highlight a small lesion or point of interest without obscuring it. It was also considered sufficiently small to not compress or cover the relevant anatomy. In addition, these fish shaped soy sauce tubes could be purchased in bulk, thus proving to be a suitable surface marker alternative from the budget perspective for selected cases.

The paint ball pellet performed well, demonstrating good visibility on all the common MRI sequences. It represents good value at a cost of AUD$12 to 20 per jar of 500 pellets. Concerns remain however, with regards to the risk of damage to equipment and property if the paint ball ruptured as the pellets are designed to burst at low force to prevent injury during game play. Due to this inherent risk of rupture, it was considered an unacceptable risk.

Oil-based capsules, particularly fish oil, are most commonly used as a commercial marker alternative (21) and are available in bulk, providing excellent value per capsule. But due to their large size (~26mm long), they are difficult to use accurately for small pathologies or on the extremities. Previous departmental experience, which has been confirmed by this study, has shown that both the fish oil capsules as well as the vitamin E capsules show up brightly on MR sequences and are comfortably and easily secured to the skin with tape. The vitamin E or fish oil capsules have been noted to be particularly useful for imaging larger body parts such as the thigh or abdomen where imaging slices are often thicker (5mm or more) such that a smaller marker may be difficult to locate. Concerns however were raised that if used in a weight bearing situation that caused rupture, the oil was difficult to clean off the patient and scanner, as well as the fish oil leaving a persistent unpleasant odour.

Performing successful MRI on children can be challenging and may require a creative approach, especially in the younger age groups. Children who have experienced a lot of medical interventions can be fearful of any and all medical procedures. Therefore we suggest it may be a great advantage to be able to produce a 'jelly baby' sweet to apply as a fiducial imaging marker when a T1_3D sequence was required, or alternatively a fish shaped soy sauce tube or Vitamin D capsule if this particular sequence was not appropriate.

An ideal alternative marker that proved to be easily visible on all the common MR sequences tested, was the Vitamin D capsule. Vitamin D capsules are available to purchase at a cost of AUD$36 for 400 capsules, which makes them affordable, disposable and readily replaceable. Vitamin D provided a bright MRI marker virtually identical to the visibility of the fish oil capsule, but was significantly smaller in size (~13mm long), making it more appealing when markers are required in imaging of the extremities, face or small pathologies. Current departmental experience has demonstrated that fish oil, vitamin E and vitamin D capsules have sufficient resilience to withstand the demands of being used as a marker, even in prolonged weight bearing situations.

Depending on the reason for imaging and the specific pathology, different MRI sequences are chosen to provide well visualised tissue contrast. For example, to demarcate the location of a tumour, a 3D T1-weighted/T2-weighted or T1 Fat Suppressed protocol would be considered more appropriate while for exploratory imaging relating to tissue infection, a T1 coronal or T2 Fat Suppressed protocol is preferable. Conversely, when MR imaging is requested for musculoskeletal

For peer review only - http://bmjopen.bmj.com/site/about/guidelines.xhtml

conditions, a Proton Density sequence is the preferred protocol to provide high intensity contrast in both fat and fluid in the bone. As such, this study provides evidence that there may not be one marker that is best suited to all imaging sequences and individual markers may provide good quality results for some but not all sequences.

A single commercial fiducial marker was analysed in the current study however it was the brand used in our clinical imaging facility making it the most relevant marker to examine. A single human participant was used and only the thigh region was scanned, but we considered this to be superior to a saline phantom. Marker visibility was analysed by a single observer but typically a single examiner views images to make judgements on visible anatomy. We acknowledge that some older clinical magnets may have a strength as low as 1.5T, however, we feel these findings relating to the FO, PB and D markers are still relevant for a lower strength magnet. Furthermore, there are a range of other MRI sequences that may be relevant for investigation of an alternative fiducial marker (e.g. Dixon imaging), however, the five sequences chosen in the current study were on the advice of the collaborating Radiology department and following a survey of local surgeons of the most commonly requested MRI sequences used for diagnostics and anatomical investigations.

In the current study, the FO, PB and D markers were all clearly visible on each of the five sequences and typically demonstrated well-defined intensity contrast with the adjacent fat/tissue. On the basis of intensity contrast, these three markers would be recommended in place of a commercial marker (CM) for use as an external fiducial marker. However, as noted in Table 1, in addition to MRI visibility of the marker, consideration of the cost, accessibility and useability of the surrogate marker are of equal importance. In light of this, the PB is less accessible, requiring specific online ordering or purchase at custom stores and the FO, while easily sourced, is too large for some applications and brings with it the risk of capsule rupture and the release of a pungent odour. Vitamin D capsules are economically purchased, small in size, readily available and bring no detrimental outcomes should they rupture. Anecdotally, the authors of this study have since used Vitamin D capsules routinely for supine musculoskeletal studies of the spine requiring extended sessions in the MR scanner and have not had any degradation or rupture, with excellent visibility of these markers observed on the resulting MR images.

For peer review only

Figure Titles

Figure 1. 18 items investigated as surrogates for the commercially available fiducial markers – Eraser, Paint Ball, Sushi Tube, Fish Oil capsule, Fruit lolly, Chewing gum, Lifesaver, Vitamin D, Blu-Tak, Plasticine, Magnesium tabsule, Tic Tac, Mentos, Mintie, Commercial Fiducial marker (IZI Multi-Modality, LifeHealthCare, Australia, included as a benchmark), Nail Polish, Coffee bean, Jelly baby sweet.

Figure 2. Markers were rigidly attached to the anterior surface of the thigh and the upper leg positioned in an RF coil. These images show the first nine items investigated.

Figure 3. Segmented volume reconstructions of the anterior thigh surface, showing an example of markers that were visible for each MRI sequence for the first acquisition of 9 markers as shown in Figure 2.

Figure 4. Workflow to measure relevant intensity parameters from each MRI dataset for each visible marker. A. 3D volume reconstruction of the T2, 3D sequence showing the transverse reslice plane (white line) and the range of z projection reslice plane (orange rectangle); B. Transverse reslice plane through the fish oil marker; C. Line selection across the marker (yellow unbroken line); D. Profile plot of intensity values along the line selection; E. Maximum intensity, transverse plane Z-projection of the fish oil capsule; F. Segmented selection tool demarcating the marker boundary (yellow broken line); G. Histogram detailing distribution of maximum intensities within the marker.

Figure 5: Reformatted transverse reslice images through the mid-height of all visible markers on the MR sequences analysed. Of the 18 markers, only 11 were visible on images from at least one of the MRI sequences – results for these 11 visible markers are shown. A blank box indicates that the marker tested was not clearly visible for a particular MR sequence.

Figure 6: Intensity ratios for all visible markers. Marker_Muscle and Marker_Fat are the ratios between maximum marker intensity and mean intensity in the fat tissue and muscle tissue, respectively. Results are shown for the five MRI sequences: Proton Density (PD), T2 weighted fat-suppressed (T2_FatSup), 3D T2 weighted (T2_3D), T1 weighted coronal (T1_coronal) and 3D T1 weighted (T1_3D)

For peer review only

For peer review only - http://bmjopen.bmj.com/site/about/guidelines.xhtml

Competing Interests & Funding Statement:

All authors have completed the ICMJE uniform disclosure form (available on request from the corresponding author) and declare: no support from any organisation for the submitted work; no financial relationships with any organisations that might have an interest in the submitted work in the previous three years; no other relationships or activities that could appear to have influenced the submitted work. No funding was received for this work.

Contributors:

MTI, CAG, JPL were involved in the conception, design and conduct of the research project, interpretation of the data and preparation of the manuscript. JPL performed the data analysis and edited the manuscript. DL wrote the first draft of the introduction and discussion. SM assisted with the design of the project, performed the imaging and assisted with the interpretation of the data and results. All authors were involved in the drafting and revising of the manuscript and gave final approval of the final version. All authors meet the ICMJE criteria for authorship. MTI and JPL are guarantors for the work and conduct of the study.

Transparency Declaration:

MTI and JPL affirm that the manuscript is an honest, accurate, and transparent account of the study being reported; that no important aspects of the study have been omitted; and that any discrepancies from the study as originally planned (and, if relevant) have been explained.

Ethical Approval:

Ethical approval was obtained from Queensland University of Technology Human Research Ethics Committee (approval # 1700000335)

Copyright:

The Corresponding Author has the right to grant on behalf of all authors and does grant on behalf of all authors, an exclusive licence on a worldwide basis to the BMJ Publishing Group Ltd to permit this article (if accepted) to be published in BMJ editions and any other BMJPGL products and sub-licences such use and exploit all subsidiary rights, as set out in our licence.

Data Sharing:

Data may be made available via the author's Institutional Research Data Storage Service (RDSS) after the paper has been published. Through the RDSS, non-institutional researchers may be granted access to this data after making a request to author (Dr J P Little, j2.little@qut.edu.au) and owner of the RDSS entry.

References

For peer review only

1. Tanaka O, Komeda H, Hirose S, Taniguchi T, Ono K, Matsuo M. Visibility of an iron-containing fiducial marker in magnetic resonance imaging for high-precision external beam prostate radiotherapy. Asia Pac J Clin Oncol. 2018;14(5):e405-e11.
2. Chan MF, Cohen GN, Deasy JO. Qualitative evaluation of fiducial markers for radiotherapy imaging. Technol Cancer Res Treat. 2015;14(3):298-304.
3. Schindel J, Muruganandham M, Pigge FC, Anderson J, Kim Y. Magnetic resonance imaging (MRI) markers for MRI-guided high-dose-rate brachytherapy: novel marker-flange for cervical cancer and marker catheters for prostate cancer. Int J Radiat Oncol Biol Phys. 2013;86(2):387-93.
4. Scherman Rydhog J, Irming Jolck R, Andresen TL, Munck Af Rosenschold P. Quantification and comparison of visibility and image artifacts of a new liquid fiducial marker in a lung phantom for image-guided radiation therapy. Med Phys. 2015;42(6):2818-26.
5. Han BH, Wallner K, Merrick G, Badiozamani K, Butler W. The effect of interobserver differences in post-implant prostate CT image interpretation on dosimetric parameters. Med Phys. 2003;30(6):1096-102.
6. Roy JN, Wallner KE, Chiu-Tsao ST, Anderson LL, Ling CC. CT-based optimized planning for transperineal prostate implant with customized template. Int J Radiat Oncol Biol Phys. 1991;21(2):483-9.
7. Roy JN, Wallner KE, Harrington PJ, Ling CC, Anderson LL. A CT-based evaluation method for permanent implants: application to prostate. Int J Radiat Oncol Biol Phys. 1993;26(1):163-9.
8. Valente G, Pitto L, Testi D, Seth A, Delp SL, Stagni R, et al. Are subject-specific musculoskeletal models robust to the uncertainties in parameter identification? PLoS ONE. 2014;9(11):e112625.
9. Freilich JM, Spiess PE, Biagioli MC, Fernandez DC, Shi EJ, Hunt DC, et al. Lipiodol as a fiducial marker for image-guided radiation therapy for bladder cancer. Int Braz J Urol. 2014;40(2):190-7.
10. Aras MH, Miloglu O, Barutcugil C, Kantarci M, Ozcan E, Harorli A. Comparison of the sensitivity for detecting foreign bodies among conventional plain radiography, computed tomography and ultrasonography. Dentomaxillofac Radiol. 2010;39(2):72-8.
11. Kim SY, Park B, Kong IG, Choi HG. Analysis of ingested foreign bodies according to age, type and location: a retrospective observational study. Clin Otolaryngol. 2016;41(6):640-5.
12. Reddy N, Fata P, Balzer A, Diaz-Marchan P, Lincoln CM. Thorn in My Spine: A case of a retained intradural extramedullary foreign body. Clin Imaging. 2017;45:118-21.
13. Shelsta HN, Bilyk JR, Rubin PA, Penne RB, Carrasco JR. Wooden intraorbital foreign body injuries: clinical characteristics and outcomes of 23 patients. Ophthalmic Plast Reconstr Surg. 2010;26(4):238-44.
14. Li J, Zhou LP, Jin J, Yuan HF. Clinical diagnosis and treatment of intraorbital wooden foreign bodies. Chin J Traumatol. 2016;19(6):322-5.
15. Shokri A, Jamalpour M, Jafariyeh B, Poorolajal J, Sabet NK. Comparison of Ultrasonography, Magnetic Resonance Imaging and Cone Beam Computed Tomography for Detection of Foreign Bodies in Maxillofacial Region. J Clin Diagn Res. 2017;11(4):TC15-TC9.
16. Pattamapaspong N, Srisuwan T, Sivasomboon C, Nasuto M, Suwannahoy P, Settakorn J, et al. Accuracy of radiography, computed tomography and magnetic resonance imaging in diagnosing foreign bodies in the foot. Radiol Med. 2013;118(2):303-10.
17. McAllister AS, Lall NU, Tawfik KO, Jones BV. The imaging appearance of crayons. Pediatr Radiol. 2017;47(6):674-80.
18. Mundada P, Kohler R, Boudabbous S, Toutous Trellu L, Platon A, Becker M. Injectable facial fillers: imaging features, complications, and diagnostic pitfalls at MRI and PET CT. Insights Imaging. 2017;8(6):557-72.
19. Javadrashid R, Golamian M, Shahrzad M, Hajalioghli P, Shahmorady Z, Fouladi DF, et al. Visibility of Different Intraorbital Foreign Bodies Using Plain Radiography, Computed Tomography, Magnetic Resonance Imaging, and Cone-Beam Computed Tomography: An In Vitro Study. Can Assoc Radiol J. 2017;68(2):194-201.
20. Havard AC, Collins DJ, Guy RL, Husband JE. Magnetic resonance behaviour of lipiodol. Clin Radiol. 1992;45(3):198-200.

For peer review only - http://bmjopen.bmj.com/site/about/guidelines.xhtml

21. Gilbert JW, Wheeler GR, Richardson GB, Herder SL, Mick GE, Gyarteng-Dakwa K, et al. Guidance of magnetic resonance imaging and placement of skin-marker localization devices. J Neurosurg Sci. 2011;55(2):85-8.

For peer review only

For peer review only - http://bmjopen.bmj.com/site/about/guidelines.xhtml

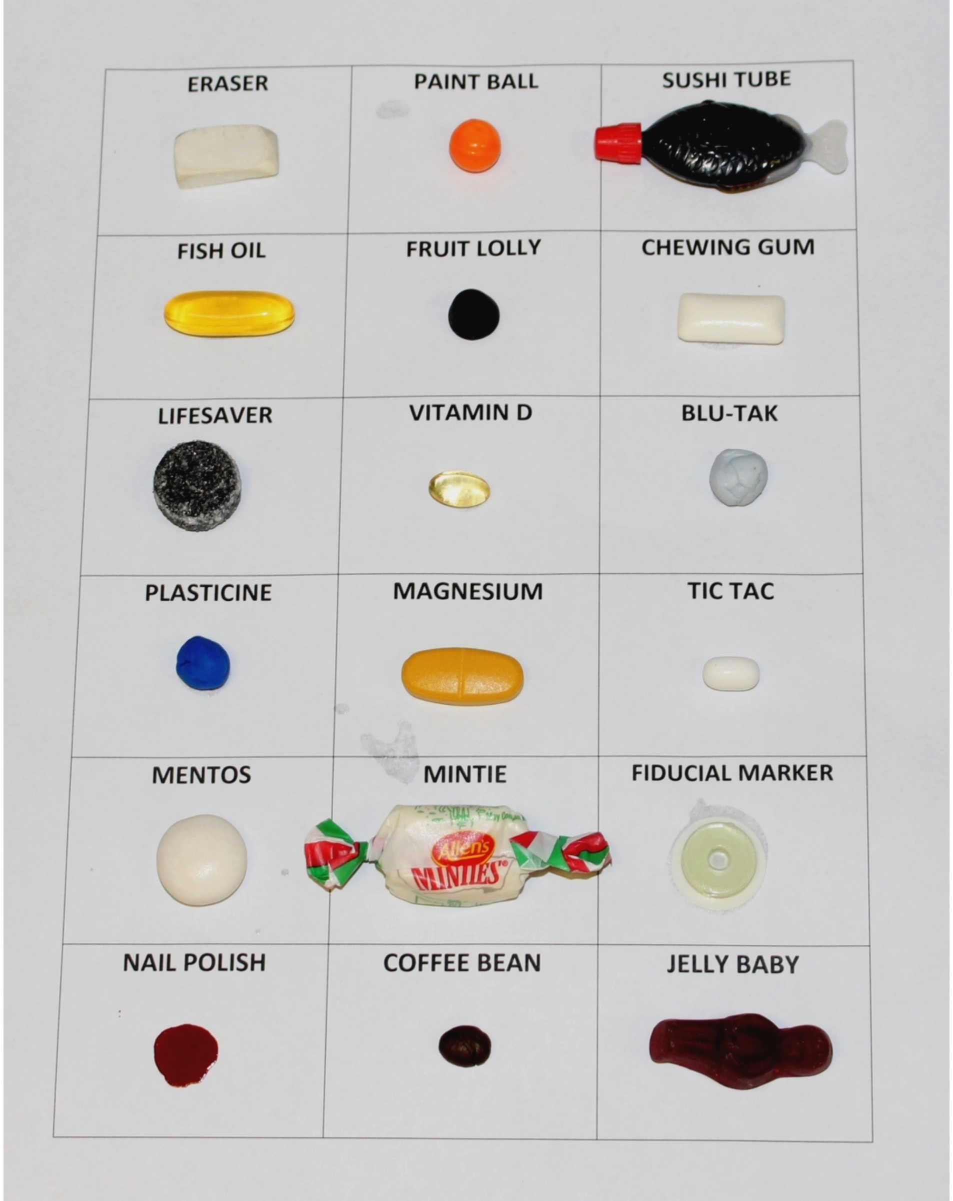

Figure 1. 18 items investigated as surrogates for the commercially available fiducial markers – Eraser, Paint Ball, Sushi Tube, Fish Oil capsule, Fruit lolly, Chewing gum, Lifesaver, Vitamin D, Blu-Tak, Plasticine, Magnesium tabsule, Tic Tac, Mentos, Mintie, Commercial Fiducial marker (IZI Multi-Modality, LifeHealthCare, Australia, included as a benchmark), Nail Polish, Coffee bean, Jelly baby sweet.

818x1036mm (72 x 72 DPI)

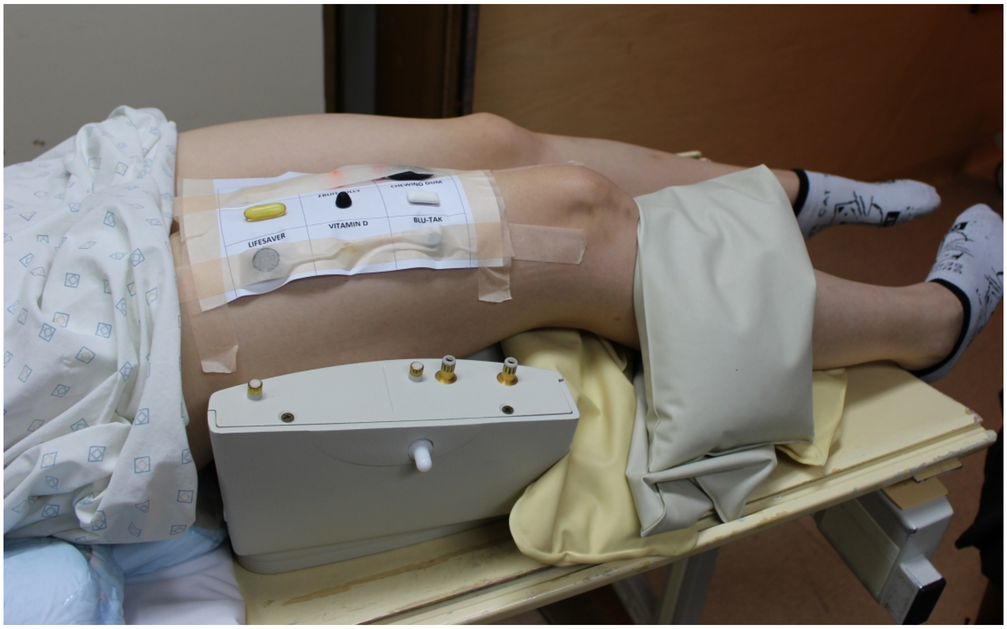

Figure 2. Markers were rigidly attached to the anterior surface of the thigh and the upper leg positioned in an RF coil. These images show the first nine items investigated.

1527x948mm (72 x 72 DPI)

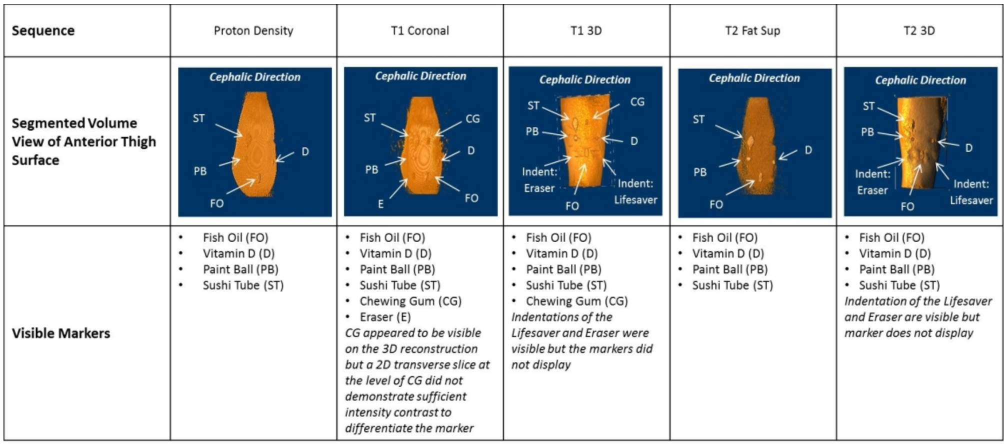

**Sequence**	Proton Density	T1 Coronal	T1 3D	T2 Fat Sup	T2 3D
**Segmented Volume View of Anterior Thigh Surface**					
**Visible Markers**	• Fish Oil (FO)<br>• Vitamin D (D)<br>• Paint Ball (PB)<br>• Sushi Tube (ST)	• Fish Oil (FO)<br>• Vitamin D (D)<br>• Paint Ball (PB)<br>• Sushi Tube (ST)<br>• Chewing Gum (CG)<br>• Eraser (E)<br>*CG appeared to be visible on the 3D reconstruction but a 2D transverse slice at the level of CG did not demonstrate sufficient intensity contrast to differentiate the marker*	• Fish Oil (FO)<br>• Vitamin D (D)<br>• Paint Ball (PB)<br>• Sushi Tube (ST)<br>• Chewing Gum (CG)<br>*Indentations of the Lifesaver and Eraser were visible but the markers did not display*	• Fish Oil (FO)<br>• Vitamin D (D)<br>• Paint Ball (PB)<br>• Sushi Tube (ST)	• Fish Oil (FO)<br>• Vitamin D (D)<br>• Paint Ball (PB)<br>• Sushi Tube (ST)<br>*Indentation of the Lifesaver and Eraser are visible but marker does not display*

Figure 3. Segmented volume reconstructions of the anterior thigh surface, showing an example of markers that were visible for each MRI sequence for the first acquisition of 9 markers as shown in Figure 2.

319x140mm (150 x 150 DPI)

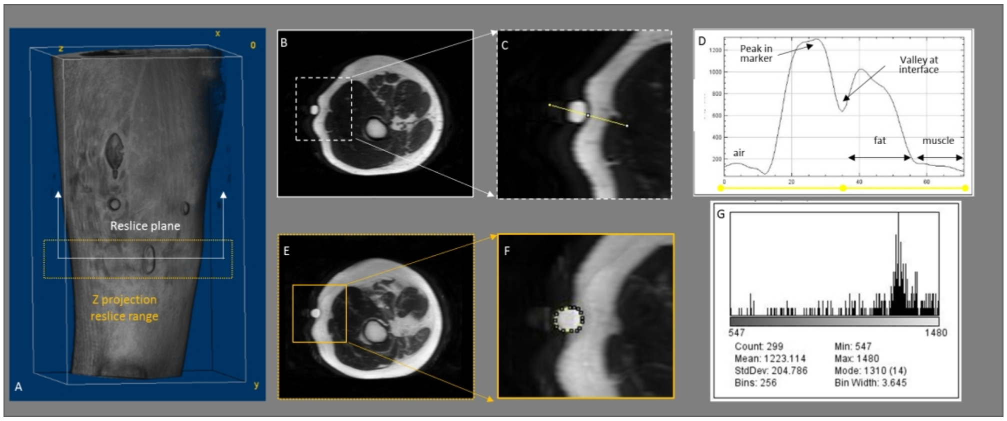

Figure 4. Workflow to measure relevant intensity parameters from each MRI dataset for each visible marker. A. 3D volume reconstruction of the T2, 3D sequence showing the transverse reslice plane (white line) and the range of z projection reslice plane (orange rectangle); B. Transverse reslice plane through the fish oil marker; C. Line selection across the marker (yellow unbroken line); D. Profile plot of intensity values along the line selection; E. Maximum intensity, transverse plane Z-projection of the fish oil capsule; F. Segmented selection tool demarcating the marker boundary (yellow broken line); G. Histogram detailing distribution of maximum intensities within the marker.

317x130mm (150 x 150 DPI)

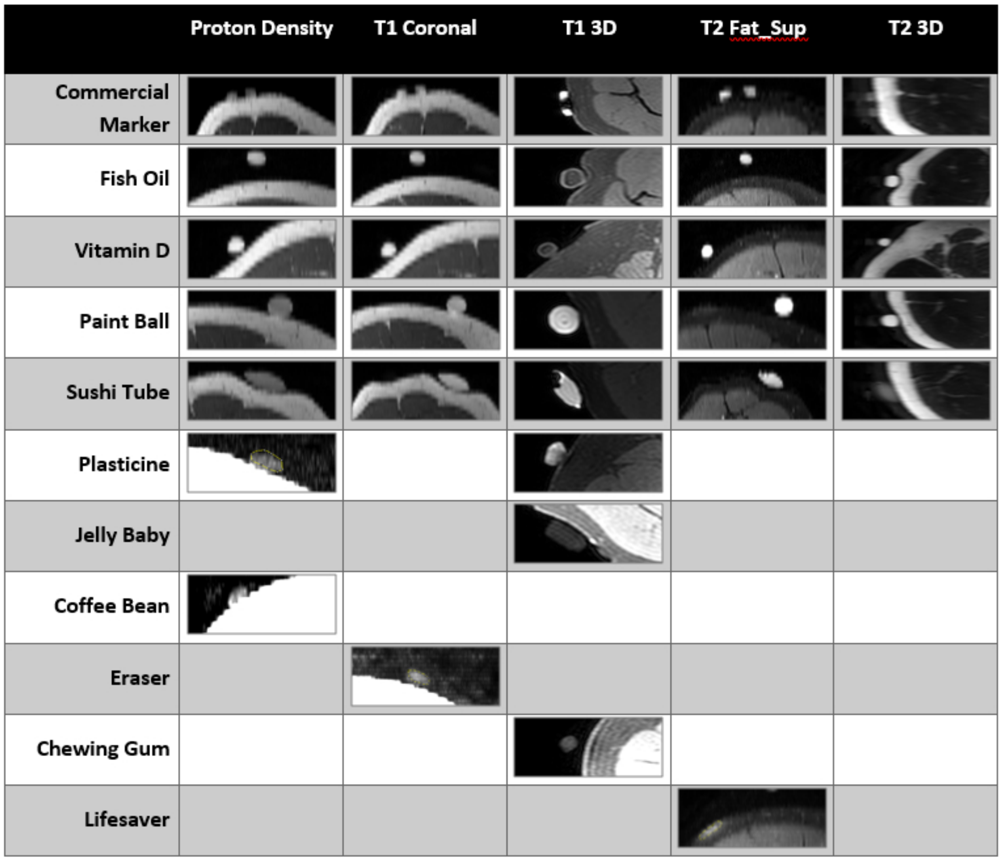

Figure 5: Reformatted transverse reslice images through the mid-height of all visible markers on the MR sequences analysed. Of the 18 markers, only 11 were visible on images from at least one of the MRI sequences – results for these 11 visible markers are shown. A blank box indicates that the marker tested was not clearly visible for a particular MR sequence.

201x173mm (96 x 96 DPI)

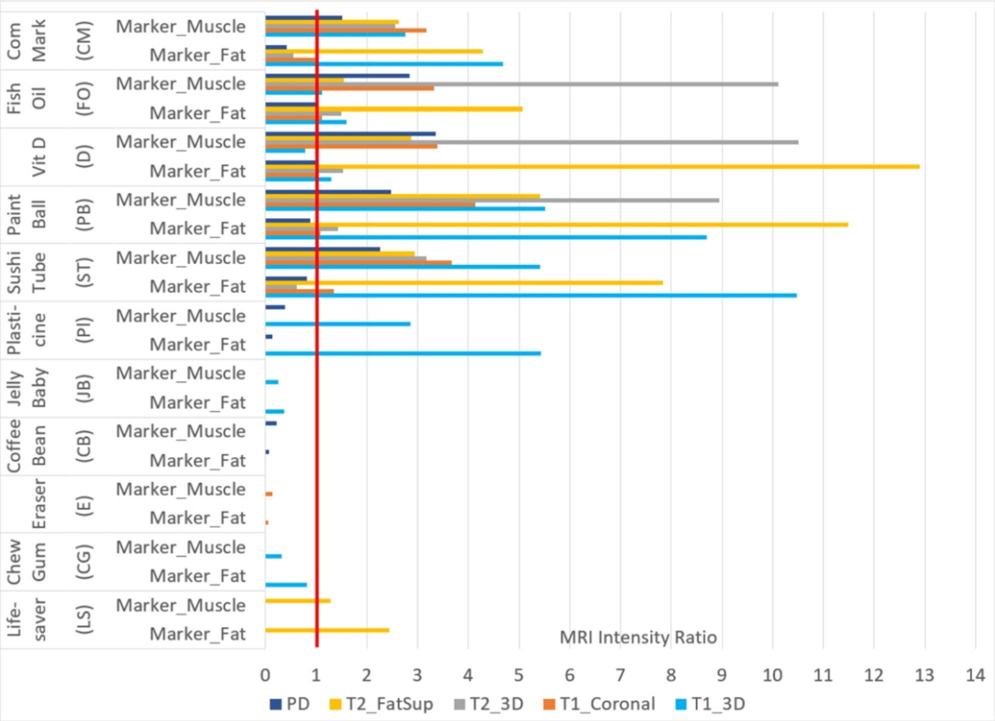

Figure 6: Intensity ratios for all visible markers. Marker_Muscle and Marker_Fat are the ratios between maximum marker intensity and mean intensity in the fat tissue and muscle tissue, respectively. Results are shown for the five MRI sequences: Proton Density (PD), T2 weighted fat-suppressed (T2_FatSup), 3D T2 weighted (T2_3D), T1 weighted coronal (T1_coronal) and 3D T1 weighted (T1_3D)

246x179mm (122 x 122 DPI)

Section & Topic	No	Item	Reported on page #
TITLE OR ABSTRACT		**Reliably Visible and Inexpensive surface Fiducial Marker options for Magnetic Resonance Imaging**	1
	1	Identification as a study of diagnostic accuracy using at least one measure of accuracy (such as sensitivity, specificity, predictive values, or AUC)	4
ABSTRACT			
	2	Structured summary of study design, methods, results, and conclusions (for specific guidance, see STARD for Abstracts)	2
INTRODUCTION			
	3	Scientific and clinical background, including the intended use and clinical role of the index test	3-4
	4	Study objectives and hypotheses	3-4
METHODS			
Study design	5	Whether data collection was planned before the index test and reference standard were performed (prospective study) or after (retrospective study)	Not applicable
Participants	6	Eligibility criteria	Not applicable
	7	On what basis potentially eligible participants were identified (such as symptoms, results from previous tests, inclusion in registry)	Not applicable
	8	Where and when potentially eligible participants were identified (setting, location and dates)	Not applicable
	9	Whether participants formed a consecutive, random or convenience series	Not applicable
Test methods	10a	Index test, in sufficient detail to allow replication	4-10
	10b	Reference standard, in sufficient detail to allow replication	4-10
	11	Rationale for choosing the reference standard (if alternatives exist)	4-10
	12a	Definition of and rationale for test positivity cut-offs or result categories of the index test, distinguishing pre-specified from exploratory	7-11
	12b	Definition of and rationale for test positivity cut-offs or result categories of the reference standard, distinguishing pre-specified from exploratory	7-11
	13a	Whether clinical information and reference standard results were available to the performers/readers of the index test	Not applicable
	13b	Whether clinical information and index test results were available to the assessors of the reference standard	Not applicable
Analysis	14	Methods for estimating or comparing measures of diagnostic accuracy	4-10
	15	How indeterminate index test or reference standard results were handled	Not applicable
	16	How missing data on the index test and reference standard were handled	Not applicable
	17	Any analyses of variability in diagnostic accuracy, distinguishing pre-specified from exploratory	Not applicable
	18	Intended sample size and how it was determined	Not applicable
RESULTS			
Participants	19	Flow of participants, using a diagram	Not applicable
	20	Baseline demographic and clinical characteristics of participants	Not applicable
	21a	Distribution of severity of disease in those with the target condition	Not applicable
	21b	Distribution of alternative diagnoses in those without the target condition	Not applicable
	22	Time interval and any clinical interventions between index test and reference standard	Not applicable
Test results	23	Cross tabulation of the index test results (or their distribution) by the results of the reference standard	11-16
	24	Estimates of diagnostic accuracy and their precision (such as 95% confidence intervals)	Not applicable
	25	Any adverse events from performing the index test or the reference standard	Not applicable
DISCUSSION			
	26	Study limitations, including sources of potential bias, statistical uncertainty, and generalisability	18
	27	Implications for practice, including the intended use and clinical role of the index test	15-17
OTHER INFORMATION			
	28	Registration number and name of registry	Not applicable
	29	Where the full study protocol can be accessed	4-10
	30	Sources of funding and other support; role of funders	17

For peer review only

For peer review only - http://bmjopen.bmj.com/site/about/guidelines.xhtml

STARD 2015

AIM

STARD stands for “Standards for Reporting Diagnostic accuracy studies”. This list of items was developed to contribute to the completeness and transparency of reporting of diagnostic accuracy studies. Authors can use the list to write informative study reports. Editors and peer-reviewers can use it to evaluate whether the information has been included in manuscripts submitted for publication.

EXPLANATION

A **diagnostic accuracy study** evaluates the ability of one or more medical tests to correctly classify study participants as having a **target condition.** This can be a disease, a disease stage, response or benefit from therapy, or an event or condition in the future. A medical test can be an imaging procedure, a laboratory test, elements from history and physical examination, a combination of these, or any other method for collecting information about the current health status of a patient.

The test whose accuracy is evaluated is called **index test.** A study can evaluate the accuracy of one or more index tests. Evaluating the ability of a medical test to correctly classify patients is typically done by comparing the distribution of the index test results with those of the **reference standard**. The reference standard is the best available method for establishing the presence or absence of the target condition. An accuracy study can rely on one or more reference standards.

If test results are categorized as either positive or negative, the cross tabulation of the index test results against those of the reference standard can be used to estimate the **sensitivity** of the index test (the proportion of participants *with* the target condition who have a positive index test), and its **specificity** (the proportion *without* the target condition who have a negative index test). From this cross tabulation (sometimes referred to as the contingency or “2x2” table), several other accuracy statistics can be estimated, such as the positive and negative **predictive values** of the test. Confidence intervals around estimates of accuracy can then be calculated to quantify the statistical **precision** of the measurements.

If the index test results can take more than two values, categorization of test results as positive or negative requires a **test positivity cut-off**. When multiple such cut-offs can be defined, authors can report a receiver operating characteristic (ROC) curve which graphically represents the combination of sensitivity and specificity for each possible test positivity cut-off. The **area under the ROC curve** informs in a single numerical value about the overall diagnostic accuracy of the index test.

The **intended use** of a medical test can be diagnosis, screening, staging, monitoring, surveillance, prediction or prognosis. The **clinical role** of a test explains its position relative to existing tests in the clinical pathway. A replacement test, for example, replaces an existing test. A triage test is used before an existing test; an add-on test is used after an existing test.

Besides diagnostic accuracy, several other outcomes and statistics may be relevant in the evaluation of medical tests. Medical tests can also be used to classify patients for purposes other than diagnosis, such as staging or prognosis. The STARD list was not explicitly developed for these other outcomes, statistics, and study types, although most STARD items would still apply.

DEVELOPMENT

This STARD list was released in 2015. The 30 items were identified by an international expert group of methodologists, researchers, and editors. The guiding principle in the development of STARD was to select items that, when reported, would help readers to judge the potential for bias in the study, to appraise the applicability of the study findings and the validity of conclusions and recommendations. The list represents an update of the first version, which was published in 2003.

More information can be found on [http://www.equator-network.org/reporting-guidelines/stard.](http://www.equator-network.org/reporting-guidelines/stard)

For peer review only

BMJ Open

Determining a Reliably Visible and Inexpensive Surface Fiducial Marker for Use in Magnetic Resonance Imaging: a Research study in a busy public radiology department.

Journal:	*BMJ Open*
Manuscript ID	bmjopen-2018-027020.R2
Article Type:	Research
Date Submitted by the Author:	29-May-2019
Complete List of Authors:	Izatt, Maree; Queensland University of Technology, Biomechanics & Spine Research Group<br>Lees, Deborah; Queensland University of Technology, Biomechanics & Spine Research Group<br>Mills, Susan; Mater Misericordiae Brisbane Ltd, Mater Medical Imaging<br>Grant, Caroline; Queensland University of Technology, Biomechanics & Spine Research Group<br>Little, Judith; Queensland University of Technology, Biomechanics & Spine Research Group
**Primary Subject Heading**:	Qualitative research
Secondary Subject Heading:	Diagnostics, Paediatrics, Patient-centred medicine, Public health, Radiology and imaging
Keywords:	Magnetic resonance imaging < RADIOLOGY & IMAGING, MRI, fiducial marker, surface marker, MRI sequence, marker visibility

SCHOLARONE™
Manuscripts

Title:

Determining a Reliably Visible and Inexpensive Surface Fiducial Marker for Use in Magnetic Resonance Imaging: a Research study in a busy public radiology department.

Authors:

Maree T. Izatt[1], B.Phty m.izatt@qut.edu.au
Project Coordinator, (ORCID ID: 0000-0002-4926-3077)

Deborah Lees[1], M.Tech (Chiro), MBBS, FRCS (Trauma & Orth) deborah.lees@health.qld.gov.au
Spine Fellow, Orthopaedic Surgeon

Susan Mills[2], B.App.Sci (MI) susan.mills@mater.org.au
MRI Unit Manager

Caroline A. Grant[1], PhD, BEng ca.grant@qut.edu.au
Postdoctoral Research Fellow, (ORCID ID: 0000-0002-5128-9903)

J. Paige Little[1], PhD, BEng j2.little@qut.edu.au
Senior Research Fellow, (ORCID ID: 0000-0001-8377-5343)

Affiliations:

[1] Biomechanics and Spine Research Group, Institute of Health and Biomedical Innovation at Centre for Children's Health Research, Queensland University of Technology, Brisbane, Australia

[2] Mater Medical Imaging, Mater Misericordiae Ltd, Brisbane, Australia

Corresponding Author:

Dr J P Little Email: j2.little@qut.edu.au; Phone: +61 7 3069 7325

For peer review only

For peer review only - http://bmjopen.bmj.com/site/about/guidelines.xhtml

Structured Abstract

Objectives

Single use commercial surface fiducial markers are used in clinical imaging for a variety of applications. The current study sought to find a new, reliably visible, easily sourced and inexpensive fiducial marker alternative for use with magnetic resonance imaging (MRI).

Design

Five commonly requested MRI sequences were determined (3D T1-weighted, T1 coronal, 3D T2-weighted, T2 fat suppressed, Proton density), to examine the visibility of 18 items (including a commercial fiducial marker).

Setting

Clinical 3T MRI scanner in Tertiary Public Hospital and University Biomedical Engineering research group

Interventions

18 marker alternatives were scanned using five common MRI sequences. Images were reformatted to obtain both an image through the mid-height of each marker and a maximum intensity z-projection image over the volume of the marker. Variations in marker intensity were profiled across each visible marker and a visibility rating defined.

Main outcome measures

Outcome measures were based on quantitative assessment of a clear intensity contrast ratio between the marker and the adjacent tissue and a qualitative assessment of visibility via a 3-point scale.

Results

The fish oil capsule, vitamin D capsule, paint ball pellet, soy sauce sushi tube and commercial markers were typically visible to a high quality on all the imaging sequences and demonstrated a clear differential in intensity contrast against the adjacent tissue. Other common items, such as plasticine 'play doh' and a soft 'Jelly baby' sweet were surprise candidates, demonstrating high quality visibility and intensity contrast for the 3D T1-weighted sequence.

Conclusions

Depending on the basis for referral and MRI sequence chosen, four alternative fiducial markers were determined to be inexpensive, easily sourced and consistently visible. Of these, the Vitamin D capsule provided an excellent balance between availability, size, cost, usability and quality of the visualised marker for all the commonly used MRI sequences analysed.

For peer review only

Strengths and Limitations

- This manuscript is the first to test for easily sourced and economical items to find reliably visible surface fiducial marker options for common MRI sequences

- Scanning the fiducial markers options on the thigh of a healthy adult female provides a superior assessment of marker visibility than analyses that use a saline phantom object.

- The study presented both quantitative and qualitative analyses of marker visibility, thus providing a more practical assessment of the ability of the radiographer/investigator to visualise the marker.

- All imaging was carried out on a 3T MRI scanner, which may be of a higher strength than is typically used in lower socio-economic tertiary care institutions.

- Five commonly utilised MRI sequences were investigated to provide the most relevant alternative marker for the majority of clinical MR imaging.

Keywords
Magnetic resonance imaging, MRI, fiducial marker, surface marker, MRI sequence, marker visibility, marker intensity, low cost marker, inexpensive marker

For peer review only

Introduction:

Fiducial markers used in the context of clinical medicine may be implanted in the body or placed on the surface and are also utilised in research for a variety of applications. Placed in the field of view, they display as distinct regions of high intensity to assist in pinpointing specific anatomical landmarks or pathologies on the acquired clinical images. Surface markers placed on a patient's skin can also provide a frame of reference for registration of medical images acquired using multiple imaging modalities, such as photography, computed tomography (CT) and magnetic resonance imaging (MRI) performed for concomitant pathologies involving multiple specialities.(1)

In radiotherapy, fiducial markers are increasingly being used to identify the tumour site, permitting image registration and assisting with guidance for treatment planning.(2-4) Oil-based surface markers have been shown to compare favourably to solid markers in terms of their contrast to noise ratio, resulting in excellent visibility.(4) Prostate marker studies also revealed that implanted titanium seeds left star/streak artefacts on CT imaging and could not be accurately localised on MR due to negative contrast (black holes).(5-7) Fiducial markers also perform a valuable role in guiding anatomical identification in musculoskeletal studies, particularly kinematic gait analyses where the location of surface markers on both mathematical gait models and the accompanying 3D MRI images provides valuable subject-specific simulation parameters.(8)

Commercially available fiducial surface markers (i.e. non-implantable) are available in a range of sizes and shapes for use in the wide variety of clinical imaging applications. They are self-adhesive and have a low, flat profile, making them comfortable for the patient. While these markers provide excellent utility, they present a substantial expense to radiology departments, with a review of the current markets showing prices ranging from $420 to $600 USD for a box of 100 (MR Spots® and IZI multi-modality, excluding shipping and tax). Being a single use product they are a considerable expense to the running costs of imaging departments or research teams utilizing MRI and requiring numerous markers.

A review of the literature to find materials that are visible in MRI revealed a paucity of publications. The majority of papers examining substance visibility were focused primarily on locating a variety of penetrating or ingested foreign bodies and just a few papers explored implanted marker options for tumour boundary markers prior to excision or radiotherapy planning.(3, 5-7, 9) Foreign bodies that had been located, ranged in material from fish and chicken bones, batteries, plastics, coins, metal and wood splinters (10-13) and it should be noted that some materials were missed even with the high sensitivity of modern MRI and CT.(14, 15) Pattamapospong *et al* (16) published results regarding the visibility of a number of substances on X-ray, CT and MRI and demonstrated a 100% specificity but only 58% sensitivity for fresh wood, dry wood, glass, plastic and porcelain using MRI. Wax crayons have proven to be easily visible on both CT and MRI with high attenuation noted on CT and a signal void on both T1 and T2 MR images. Interestingly, it was noted that crayon colour influenced the degree of visibility. Different pigments used in crayons and in paints resulted in distinctive colours as well as MRI appearance.(17) Injectable facial fillers (hyaluronic acid, collagen and polyalkylimide-polyacrylamide hydrogels), silicone and calcium hydroxyapatite have also been well visualised on MRI but can be confused with malignant features, so are not considered good marker substances.(18) Metal is well visualised on many modalities (10) but due to its ferrous properties, is contra-indicated in the field of MRI. Metal, even if MR conditional, results in significant image attenuation and artefact. For these reasons, an iron tablet as a makeshift marker may be well

For peer review only - http://bmjopen.bmj.com/site/about/guidelines.xhtml

visualised but the resulting artefact renders it unacceptable. Studies looking at the visibility of plastic, stone, glass and graphite revealed that both glass and plastic foreign bodies are not consistently visible on MRI or plain radiographs; despite showing up well on CT.(16, 19)

A review of the physics of MRI suggested materials with high water or fat content were likely items that would be readily detectable. Fiducial markers should be easily identifiable and clearly visible on clinical images, allowing the target anatomy or volume to be clearly and completely visualised. Therefore a critical objective of this study was to assess the validity of various 'everyday' items, which could be easily and economically sourced, and provide a reliably visible fiducial marker as an alternative to the comparatively expensive, single use commercial fiducial markers. We hypothesised that an inexpensive, readily sourced, robust surface fiducial marker could be isolated, that consistently demonstrated at least the same level of visibility as a commercial fiducial marker, when viewed on the most commonly performed MRI sequences.

Methods:

Seventeen everyday items and a commercial fiducial marker were selected for analysis, either from the literature or anecdotal reports (Figure 1). The important considerations relating to the selection of a suitable surrogate for commercial fiducial markers were identified and are outlined in Table 1. In addition to the commercial fiducial marker, two of these items had been used in our local hospital medical imaging department; the paint ball for MR scans in a pilot research project, and the fish oil capsule which was in regular use to avoid using a commercial marker whenever possible. While these two non-commercial markers provided excellent results in terms of intensity contrast and visualisation on MR images, to date a definitive comparison of the image contrast provided by these items has not been undertaken and these alternatives did not meet all the desired requirements of the ideal alternative fiducial marker.

For peer review only

Table 1. Important considerations relating to the choice of a suitable fiducial marker for MR imaging

Factors for consideration	
**Accessibility**	Easily acquired, readily replaced, and preferably locally sourced without high shipment costs or transit time
**Use**	Sufficiently robust to tolerate body weight if placed between the participant and scanner bed<br>Suitably compliant or flat to ensure minimal discomfort to the patient if marker is in a load bearing location (e.g. Posterior superior iliac spine for a supine MRI)
**Biochemistry**	Chemically inert, hypoallergenic
**Economy**	Low acquisition cost per marker given markers are single use items
**Medical Physics**	Sufficient hydrogen content to permit high intensity contrast to surrounding tissue
**Fixation**	Reliably affixed to the patient's external anatomy

Imaging Details:

Table 2. MRI sequence details

	Imaging Protocol		Acquisition plane	Field of View (FOV) (mm)	Repetition time (TR) / Echo time (TE) (msec)	Resolution (Pixel width x height x slice spacing, mm)
**1**	Proton density (PD)		Coronal	300 x 300 x 44	2,435/35	0.1465 x 0.1465 x 2.2
**2**	T1 weighted, Spin echo		Coronal	300 x 300 x 44	661/10	0.1465 x 0.1465 x 2.2
**3**	T1 weighted, 3D Fast field echo (FFE)		Sagittal	300 x 300 x 175	25/1.4	0.469 x 0.469 x 0.5
**4**	T2 weighted, fat suppressed		Coronal	300 x 300 x 44	4,448/65	0.195 x 0.195 x 2.2
**5**	T2 weighted, 3D		Sagittal	300 x 300 x 175	2,473/72	0.469 x 0.469 x 0.5

For peer review only - http://bmjopen.bmj.com/site/about/guidelines.xhtml

The selection of items underwent MR scanning in a clinical medical imaging department using a 3-T Philips Achieva MRI scanner. Scanning parameters vary between different machine manufacturers and even between different scanner models from the same manufacturer. However, this particular scanner was selected as it services a busy hospital radiology department in a metropolitan city, and thus, provides a broad range of exploratory and treatment-based MRI scanning services. Further to this, the 3-T magnet strength is a typical specification for scanners in tertiary care facilities in Australia and internationally.

Following the advice from the senior MR radiographers and a survey of local surgeons, five different sequences were performed (Table 2) to cover the breadth of MR imaging studies typically performed with a requirement for inclusion of surface markers. All tested markers were attached to the anterior thigh of a healthy female participant, aged 27 years, who had provided informed consent. A radiofrequency coil (8-channel knee coil) was positioned beneath the thigh and the markers attached to the anterior thigh surface (Figure 1). Due to physical size constraints, the marker selection was attached to the volunteer's leg in two separate acquisitions. In all the MR sequences detailed in Table 2, the scanning parameters were constant for both acquisitions.

Images were saved in Digital Imaging and Communications in Medicine (DICOM) format and analysed using ImageJ (U. S. National Institute of Health Open-source software, Maryland, USA; https://imagej.nih.gov/ij/). A similar process was utilised to assess the results from each of the five different imaging sequences (Table 2) and involved first viewing the 3D stack as a segmented volume using the ImageJ Plugin, 'Volume Viewer 2.01' (K U Barthel, Internationale Medieninformatik, HTW Berlin, Germany) (Figure 3).

In the first instance, a qualitative evaluation of the marker visibility was carried out to subjectively assess the relative ease with which each marker could be visualised and the marker edges demarcated. Viewing the data as a 3D volume permitted an initial assessment of which markers exhibited an intensity that was visible on MR images (Figure 3). If the marker was visible, the visibility was rated on a 3-point Likert scale with 3 representing 'very clear', 2 representing 'edges visible but fuzzy' and 1 representing 'not easily visible'. If a visibility rating of one was observed, this low rating suggested that while the marker was discernibly visible on the MR images, the quality of visibility was poor and the marker would not be recommended for use in clinical scanning using that particular MR sequence. Using this scale, a mean visibility rating across the five MRI sequences was calculated for each fiducial marker. While a mean visibility rating >0 indicated that the marker could be seen on at least one of the MRI sequences, a mean visibility rating greater than 2 was preferable as it indicated a consistently good quality visibility on all of the MRI sequences performed.

In all cases, MR images were reformatted and processed as 16-bit images, with an intensity range from 0 to 65,536. A quantitative evaluation of the marker quality was carried out to assess the visibility, artefacts and distortion of marker boundaries created by each item on each MRI sequence. This evaluation was carried out using the steps outlined below and shown visually in Figure 4.

1. Reformatted Image Stack: The DICOM stacks were reformatted to create a sequential stack of transverse reslice images, with consideration of the relevant pixel and slice spacing for each dataset (Table 2).
2. Intensity Profile Plot: Using the 3D volume reconstructions for each MRI sequence as a reference (Figure 3, Figure 4 A), the reformatted transverse reslice through the mid-height of each marker was located (Figure 4 B). A line selection (Figure 4 C) was drawn through the marker, such that

For peer review only - http://bmjopen.bmj.com/site/about/guidelines.xhtml

the mid-point on the line was positioned at the interface between the marker and the skin and the endpoints were located in air and muscle tissue, respectively. The intensity profile along this line selection (Figure 4 D) was used to objectively compare the marker signal and visibility. The peak intensity in the marker, minimum intensity at the interface between the marker and tissue, mean intensity in the fat tissue and mean intensity in the muscle tissue were recorded from the profile.

3. <u>Maximum Intensity Z-projection</u>: A two-dimensional (2D) image with each pixel intensity equivalent to the maximum intensity along a Z trajectory over a given range of stack images was created (Figure 4 E) The image range was defined to encompass the upper and lower limits of the marker, as viewed on the 3D volume reconstruction (Figure 4 A, orange broken line).
4. <u>Intensity Histogram and Maximum Marker Intensity</u>: A segmented line tool (Figure 4 F) was used to demarcate the outer profile of the marker on the 2D Z-projection image (Figure 4 E). A histogram showing the distribution of maximum intensities within this profile for the marker was used to record the maximum and mean marker intensity (Figure 4 G).

In some instances (Figure 3: T1_3D – Chewing Gum; T1 Coronal – Eraser) the intensity range exhibited by the marker was very low in comparison to the background intensity, making the marker boundaries difficult to differentiate from the surrounding regions. However, these markers were still included in the analysis as it was possible to create a profile plot (Figure 4 D) on a transverse plane through the mid-height of the marker, with a measurable intensity gradient across the marker.

Assessment of Intensity Values:

In order to objectively assess whether the markers provided an acceptable visualisation on the images produced for each MR sequence analysed, three criteria were defined to assess the results. It was expected that the markers should; a) Provide a pixel intensity that was sufficiently above the background intensity of air to result in a clear visual contrast; b) Demonstrate a pixel intensity ratio at the interface between the marker and adjacent epidermis/fat tissue (i.e. peak marker intensity divided by interface intensity/mean fat tissue intensity) greater than 1; and c) Demonstrate a pixel intensity ratio between the marker and the muscle tissue (i.e. peak marker intensity divided by mean muscle intensity) greater than 1.

The ratios were intended to provide a numeric value that aligned with the perceptual and visual decision making process a person undertakes when determining whether a marker is visualised at an acceptable quality. To indicate the quality of marker visualisation, this ratio of unity was defined after viewing all markers over the five MRI sequences and in combination with the visual assessment of visibility rating.

Depending on the MRI sequence, the fat and muscle tissue adjacent to the marker displayed over different intensity ranges. The ratio between the fat or muscle tissue and marker intensity was considered critical to be able to differentiate the marker from its surroundings. For this reason the intensity ratio was considered of more importance than the absolute value of the pixel intensity.

Patient and Public Involvement:

This research was done without patient or public involvement as an aspect of a larger study yet to be performed. Participants in the larger study will be asked to be involved in the design of the subjective assessment methods.

For peer review only - http://bmjopen.bmj.com/site/about/guidelines.xhtml

Results

Of the 18 markers investigated, five were consistently visible for all imaging sequences, but this visibility was of differing quality (Table 3). The Fish Oil (FO), Vitamin D (D), Paintball (PB), Soy Sauce Sushi Tube (ST) and Commercial Marker (CM) were typically visible to a high quality (i.e. rating 2 or 3) for all sequences (Table 3). A visibility rating of 1 on only one of the MR sequences was observed for the Eraser (E), Chewing Gum (CG), Lifesaver (LS), and Coffee Bean (CB), indicating the quality of visibility was poor. Surprisingly, the Pl marker was visible to a high quality (rating 3) for the T1_3D sequence, but had little (rating = 1 for PD sequence) or no visibility for the other sequences. Similarly, the Jelly Baby was also remarkable in being well visualised on the T1_3D sequence with a rating of 2, but had no visibility scored for the other MR sequences.

A mean visibility score greater than 2 was observed for the PB, FO, D and CM markers (Table 3).

For peer review only

Table 3. Visibility rating for each marker on each MRI sequence. If not visible, the marker rating = 0. If visible, marker visibility rating was either 1 ('visible, but not easily'), 2 ('edges visible but fuzzy') or 3 ('very clear').

	Proton Density	T1 Coronal	T1_3D	T2 Fat_Sup	T2_3D	MEAN RATING
**Commercial Marker**	2	2	3	3	2	2.4
**Fish Oil**	2	2	3	2	3	2.4
**Vitamin D**	2	2	3	3	3	2.6
**Paint Ball**	2	2	3	3	3	2.6
**Soy Sauce Sushi Tube**	2	2	3	2	1	2.0
**Plasticine**	1	0	3	0	0	0.8
**Jelly Baby**	0	0	2	0	0	0.4
**Coffee Bean**	1	0	0	0	0	0.2
**Eraser**	0	1	0	0	0	0.2
**Chewing Gum**	0	0	1	0	0	0.2
**Lifesaver**	0	0	0	1	0	0.2
**Fruit Lolly**	0	0	0	0	0	0
**BluTak**	0	0	0	0	0	0
**Magnesium**	0	0	0	0	0	0
**TicTac**	0	0	0	0	0	0
**Mentos**	0	0	0	0	0	0
**Mintie**	0	0	0	0	0	0
**Nail Polish**	0	0	0	0	0	0

Figure 5 shows a summary of the visible markers on the five MRI sequences. In each case, the intensity range for the image was selected such that the intensity contrast within the image could best display the marker. These images do not include image filtering or contrast enhancement. Viewing the reslice images in Figure 5 in combination with the visibility ratings in Table 3 demonstrated that the best quality marker visibility for the T1_3D, T1 coronal, T2 Fat_Sup and Proton Density sequences was the PB, ST, FO and D; and for the T2_3D sequence was the PB, FO and D. The CM was best visualised on the T1_3D and T2 Fat_Sup sequences.

Intensity ratios between the maximum marker intensity and the mean intensity in either the fat tissue or muscle tissue are shown in Figure 6.

For peer review only - http://bmjopen.bmj.com/site/about/guidelines.xhtml

For the PD sequence, while the CM, FO, D, PB and ST markers were easily visible (Figure 5, Figure 6), the marker intensity was numerically similar to the fat tissue, resulting in marker-to-fat ratios near or below one. The PL and CB were less easily differentiated from the nearby fat tissue (Figure 6).

For the T2 fat suppressed sequence, there was a distinct differential between the maximum intensity in the marker and the mean intensity in the fat tissue, making all observable markers (CM, FO, D, PB, ST and LS) clearly visible (Figure 6).

The commercial marker (CM) was not clearly visualised for the 3D T2-weighted sequence (Table 3, Figure 5), and had a marker-to-fat ratio <1 (Figure 6). While the ST tended to result in good visibility (Table 3, Figure 5), for the 3D T2-weighted sequence the marker intensity was less than the muscle tissue and not easily differentiated from the background air intensity. Aside from this, the 3D T2-weighted sequence showed an intensity ratio >>1 and thus clear intensity contrast for the marker-to-muscle ratio (>1) and marker-to-fat ratio, for all markers.

While the eraser (E) was visible on the T1 coronal sequence, the intensity ratios suggested that the intensity contrast between the marker and adjacent muscle/fat was not of a good quality (Table 3, Figure 5). This was supported by the observed intensity ratio <1 for both the marker-to-muscle and marker-to-fat ratios (Figure 6). For the other visible markers, the intensity ratios were all ≥ 1.
With the exception of the chewing gum (CG) and jelly baby (JB), the intensity ratios for marker-to-fat were all >1, for the 3D_T1-weighted sequence. In the case of the PB and ST marker, since these were comprised of a non-oil based substance, the intensity contrast and marker-to-fat/marker-to-muscle intensity ratios were >>1 (Figure 6). These latter two markers also resulted in a high visibility rating (Table 3, Figure 5).

Discussion

Fiducial markers are used for calibration of the imaging equipment, providing a reference measurement for templating software, locating specific boundaries for radiotherapy planning, orienting an image and very importantly, to identify a specific area of interest in the case of diagnostic queries. It is thus essential to have a marker that is detectable and clearly discernible from bone and soft tissues when placed in the visual field for a number of common MR sequences. Single use, commercially available fiducial markers make up a significant component of the imaging department's outgoings and in an attempt to reduce costs, the current study was a practical exercise designed to image a number of commonly available everyday items to assess their suitability to function as an effective surface fiducial MRI marker substitute. The current study aimed to provide additional marker options useful for personalised musculoskeletal kinematic models as well as for clinical pathologies and research projects utilizing MRI.

A single use, commercially available surface fiducial marker was evaluated in this study alongside seventeen potential alternatives and its performance was surprisingly less impressive than some of the more common and affordable items tested. The commercial marker is considered prohibitively expensive (AUD$ 6 to 10 per marker) and is therefore used selectively for specific neurosurgical imaging needs only. Makeshift markers of various types have been anecdotally trialled in clinical radiology departments over the years. Discussions with our hospital imaging staff revealed that almonds, Vitamin E capsules and condiment packets had previously been trialled. Packaging rupture

For peer review only - http://bmjopen.bmj.com/site/about/guidelines.xhtml

was a risk and unfortunately these makeshift markers caused issues with patient comfort, and inconsistent visibility on imaging.

In the current study, the FO, PB and D markers were all clearly visible on each of the five sequences and typically demonstrated well-defined intensity contrast with the adjacent fat/tissue. On the basis of intensity contrast, these three markers would be recommended in place of a commercial marker (CM) for use as an external fiducial marker.

However, as noted in Table 1, in addition to MRI visibility of the marker, consideration of the cost, accessibility and useability of the surrogate marker are of equal importance. In light of this, the PB is less accessible, requiring specific online ordering or purchase at custom stores. Additionally concerns remain with regards to the risk of damage to equipment and property if the paint ball ruptured as the pellets are designed to burst at low force to prevent injury during game play. The FO, while clearly visualised and sourced, are large in size making them difficult to use accurately for small pathologies or on extremities. They also bring with them the risk of capsule rupture and the release of a pungent odour.

Oil-based capsules are commonly used as a commercial marker alternative (20). Vitamin D capsules are economically purchased (cost of AUD$36 for 400 capsules), small in size, readily available and bring no detrimental outcomes should they rupture. Vitamin D provided a bright MRI marker virtually identical to the visibility of the FO capsule, but was significantly smaller in size (~13mm long), making it more appealing when markers are required in imaging of the extremities, face or small pathologies. Anecdotally, the authors of this study have since used Vitamin D capsules routinely for supine musculoskeletal studies of the spine requiring multiple markers affixed throughout extended sessions in the MR scanner and have not had any degradation or rupture, with excellent visibility of these markers observed on the resulting MR images.

While the Vitamin D capsule was the final fiducial marker of choice, a discussion of other markers that were well visualised on some sequences is relevant given the aim of this study. Fluids are more readily identifiable on MRI and lipid-based markers have proven to be consistently reliable.(9, 21) The capped sushi soy sauce tubes tested in the current study were reliably visible on all the MRI sequences analysed and were considered by the MR radiographers as a useful alternative marker candidate. They suggested one or more of the fish-shaped tubes could act as a type of “pointer” to highlight a small lesion or point of interest without obscuring it. It was also considered sufficiently small to not compress or cover the relevant anatomy.

Performing successful MRI on children can be challenging and may require a creative approach, especially in the younger age groups. Children who have experienced a lot of medical interventions can be fearful of any and all medical procedures. Therefore we suggest it may be a great advantage to be able to produce a ‘jelly baby’ sweet to apply as a fiducial imaging marker when a T1_3D sequence was required, or alternatively a fish shaped soy sauce tube or Vitamin D capsule if this particular sequence was not appropriate.

Depending on the reason for imaging and the specific pathology, different MRI sequences are chosen to provide well visualised tissue contrast. For example, to demarcate the location of a tumour, a 3D T1-weighted/T2-weighted or T1 Fat Suppressed protocol would be considered more appropriate while for exploratory imaging relating to tissue infection, a T1 coronal or T2 Fat Suppressed protocol is preferable. Conversely, when MR imaging is requested for musculoskeletal conditions, a Proton Density sequence is the preferred protocol to provide high intensity contrast in

For peer review only - http://bmjopen.bmj.com/site/about/guidelines.xhtml

both fat and fluid in the bone. As such, this study provides evidence that there may not be one marker that is best suited to all imaging sequences and individual markers may provide good quality results for some but not all sequences (Table 3, Figure 5).

Regarding study limitations, while a single commercial fiducial marker was analysed in the current study, it was the brand used in our clinical imaging facility making it the most relevant marker to examine. A single human participant was used and only the thigh region was scanned, but we considered this to be superior to a saline phantom. Marker visibility was analysed by a single observer but typically in the clinical setting a single examiner views images to make judgements on visible anatomy. We acknowledge that some older clinical magnets may have a strength as low as 1.5T, however, we feel these findings relating to the FO, PB and D markers are still relevant for a lower strength magnet. Furthermore, there are a range of other additional MRI sequences that may be relevant for investigation of an alternative fiducial marker (e.g. Dixon imaging), however, the five sequences chosen in the current study were on the advice of the collaborating Radiology Department and following a survey of local surgeons of the most commonly requested MRI sequences used for diagnostics and anatomical investigations.

For peer review only

Figure Titles

Figure 1. 18 items investigated as surrogates for the commercially available fiducial markers – Eraser, Paint Ball, Sushi Tube, Fish Oil capsule, Fruit lolly, Chewing gum, Lifesaver, Vitamin D, Blu-Tak, Plasticine, Magnesium tabsule, Tic Tac, Mentos, Mintie, Commercial Fiducial marker (IZI Multi-Modality, LifeHealthCare, Australia, included as a benchmark), Nail Polish, Coffee bean, Jelly baby sweet.

Figure 2. Markers were rigidly attached to the anterior surface of the thigh and the upper leg positioned in an RF coil. These images show the first nine items investigated.

Figure 3. Segmented volume reconstructions of the anterior thigh surface, showing an example of markers that were visible for each MRI sequence for the first acquisition of 9 markers as shown in Figure 2.

Figure 4. Workflow to measure relevant intensity parameters from each MRI dataset for each visible marker. A. 3D volume reconstruction of the T2, 3D sequence showing the transverse reslice plane (white line) and the range of z projection reslice plane (orange rectangle); B. Transverse reslice plane through the fish oil marker; C. Line selection across the marker (yellow unbroken line); D. Profile plot of intensity values along the line selection; E. Maximum intensity, transverse plane Z-projection of the fish oil capsule; F. Segmented selection tool demarcating the marker boundary (yellow broken line); G. Histogram detailing distribution of maximum intensities within the marker.

Figure 5: Reformatted transverse reslice images through the mid-height of all visible markers on the MR sequences analysed. Of the 18 markers, only 11 were visible on images from at least one of the MRI sequences – results for these 11 visible markers are shown. A blank box indicates that the marker tested was not clearly visible for a particular MR sequence.

Figure 6: Intensity ratios for all visible markers. Marker_Muscle and Marker_Fat are the ratios between maximum marker intensity and mean intensity in the fat tissue and muscle tissue, respectively. Results are shown for the five MRI sequences: Proton Density (PD), T2 weighted fat-suppressed (T2_FatSup), 3D T2 weighted (T2_3D), T1 weighted coronal (T1_coronal) and 3D T1 weighted (T1_3D)

For peer review only

Competing Interests & Funding Statement:

All authors have completed the ICMJE uniform disclosure form (available on request from the corresponding author) and declare: no support from any organisation for the submitted work; no financial relationships with any organisations that might have an interest in the submitted work in the previous three years; no other relationships or activities that could appear to have influenced the submitted work. No funding was received for this work.

Contributors:

MTI, CAG, JPL were involved in the conception, design and conduct of the research project, interpretation of the data and preparation of the manuscript. JPL performed the data analysis and edited the manuscript. DL wrote the first draft of the introduction and discussion. SM assisted with the design of the project, performed the imaging and assisted with the interpretation of the data and results. All authors were involved in the drafting and revising of the manuscript and gave final approval of the final version. All authors meet the ICMJE criteria for authorship. MTI and JPL are guarantors for the work and conduct of the study.

Transparency Declaration:

MTI and JPL affirm that the manuscript is an honest, accurate, and transparent account of the study being reported; that no important aspects of the study have been omitted; and that any discrepancies from the study as originally planned (and, if relevant) have been explained.

Ethical Approval:

Ethical approval was obtained from Queensland University of Technology Human Research Ethics Committee (approval # 1700000335)

Copyright:

The Corresponding Author has the right to grant on behalf of all authors and does grant on behalf of all authors, an exclusive licence on a worldwide basis to the BMJ Publishing Group Ltd to permit this article (if accepted) to be published in BMJ editions and any other BMJPGL products and sub-licences such use and exploit all subsidiary rights, as set out in our licence.

Data Sharing:

Data may be made available via the author's Institutional Research Data Storage Service (RDSS) after the paper has been published. Through the RDSS, non-institutional researchers may be granted access to this data after making a request to author (Dr J P Little, j2.little@qut.edu.au) and owner of the RDSS entry.

For peer review only

References

1. Tanaka O, Komeda H, Hirose S, Taniguchi T, Ono K, Matsuo M. Visibility of an iron-containing fiducial marker in magnetic resonance imaging for high-precision external beam prostate radiotherapy. Asia Pac J Clin Oncol. 2018;14(5):e405-e11.
2. Chan MF, Cohen GN, Deasy JO. Qualitative evaluation of fiducial markers for radiotherapy imaging. Technol Cancer Res Treat. 2015;14(3):298-304.
3. Schindel J, Muruganandham M, Pigge FC, Anderson J, Kim Y. Magnetic resonance imaging (MRI) markers for MRI-guided high-dose-rate brachytherapy: novel marker-flange for cervical cancer and marker catheters for prostate cancer. Int J Radiat Oncol Biol Phys. 2013;86(2):387-93.
4. Scherman Rydhog J, Irming Jolck R, Andresen TL, Munck Af Rosenschold P. Quantification and comparison of visibility and image artifacts of a new liquid fiducial marker in a lung phantom for image-guided radiation therapy. Med Phys. 2015;42(6):2818-26.
5. Han BH, Wallner K, Merrick G, Badiozamani K, Butler W. The effect of interobserver differences in post-implant prostate CT image interpretation on dosimetric parameters. Med Phys. 2003;30(6):1096-102.
6. Roy JN, Wallner KE, Chiu-Tsao ST, Anderson LL, Ling CC. CT-based optimized planning for transperineal prostate implant with customized template. Int J Radiat Oncol Biol Phys. 1991;21(2):483-9.
7. Roy JN, Wallner KE, Harrington PJ, Ling CC, Anderson LL. A CT-based evaluation method for permanent implants: application to prostate. Int J Radiat Oncol Biol Phys. 1993;26(1):163-9.
8. Valente G, Pitto L, Testi D, Seth A, Delp SL, Stagni R, et al. Are subject-specific musculoskeletal models robust to the uncertainties in parameter identification? PLoS ONE. 2014;9(11):e112625.
9. Freilich JM, Spiess PE, Biagioli MC, Fernandez DC, Shi EJ, Hunt DC, et al. Lipiodol as a fiducial marker for image-guided radiation therapy for bladder cancer. Int Braz J Urol. 2014;40(2):190-7.
10. Aras MH, Miloglu O, Barutcugil C, Kantarci M, Ozcan E, Harorli A. Comparison of the sensitivity for detecting foreign bodies among conventional plain radiography, computed tomography and ultrasonography. Dentomaxillofac Radiol. 2010;39(2):72-8.
11. Kim SY, Park B, Kong IG, Choi HG. Analysis of ingested foreign bodies according to age, type and location: a retrospective observational study. Clin Otolaryngol. 2016;41(6):640-5.
12. Reddy N, Fata P, Balzer A, Diaz-Marchan P, Lincoln CM. Thorn in My Spine: A case of a retained intradural extramedullary foreign body. Clin Imaging. 2017;45:118-21.
13. Shelsta HN, Bilyk JR, Rubin PA, Penne RB, Carrasco JR. Wooden intraorbital foreign body injuries: clinical characteristics and outcomes of 23 patients. Ophthalmic Plast Reconstr Surg. 2010;26(4):238-44.
14. Li J, Zhou LP, Jin J, Yuan HF. Clinical diagnosis and treatment of intraorbital wooden foreign bodies. Chin J Traumatol. 2016;19(6):322-5.
15. Shokri A, Jamalpour M, Jafariyeh B, Poorolajal J, Sabet NK. Comparison of Ultrasonography, Magnetic Resonance Imaging and Cone Beam Computed Tomography for Detection of Foreign Bodies in Maxillofacial Region. J Clin Diagn Res. 2017;11(4):TC15-TC9.
16. Pattamapaspong N, Srisuwan T, Sivasomboon C, Nasuto M, Suwannahoy P, Settakorn J, et al. Accuracy of radiography, computed tomography and magnetic resonance imaging in diagnosing foreign bodies in the foot. Radiol Med. 2013;118(2):303-10.
17. McAllister AS, Lall NU, Tawfik KO, Jones BV. The imaging appearance of crayons. Pediatr Radiol. 2017;47(6):674-80.
18. Mundada P, Kohler R, Boudabbous S, Toutous Trellu L, Platon A, Becker M. Injectable facial fillers: imaging features, complications, and diagnostic pitfalls at MRI and PET CT. Insights Imaging. 2017;8(6):557-72.
19. Javadrashid R, Golamian M, Shahrzad M, Hajalioghli P, Shahmorady Z, Fouladi DF, et al. Visibility of Different Intraorbital Foreign Bodies Using Plain Radiography, Computed Tomography, Magnetic Resonance Imaging, and Cone-Beam Computed Tomography: An In Vitro Study. Can Assoc Radiol J. 2017;68(2):194-201.

For peer review only - http://bmjopen.bmj.com/site/about/guidelines.xhtml

20. Gilbert JW, Wheeler GR, Richardson GB, Herder SL, Mick GE, Gyarteng-Dakwa K, et al. Guidance of magnetic resonance imaging and placement of skin-marker localization devices. J Neurosurg Sci. 2011;55(2):85-8.
21. Havard AC, Collins DJ, Guy RL, Husband JE. Magnetic resonance behaviour of lipiodol. Clin Radiol. 1992;45(3):198-200.

For peer review only

For peer review only - http://bmjopen.bmj.com/site/about/guidelines.xhtml

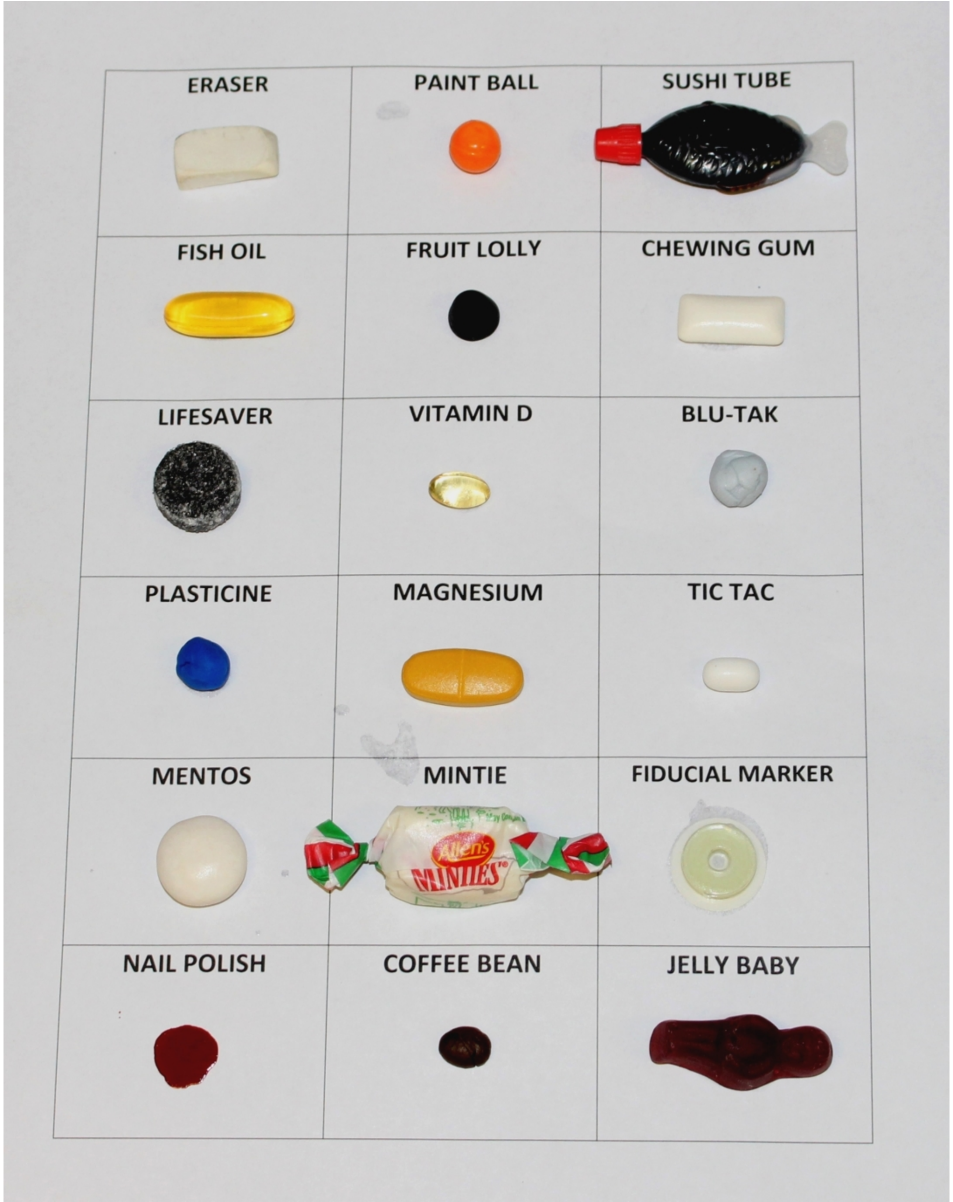

Figure 1. 18 items investigated as surrogates for the commercially available fiducial markers – Eraser, Paint Ball, Sushi Tube, Fish Oil capsule, Fruit lolly, Chewing gum, Lifesaver, Vitamin D, Blu-Tak, Plasticine, Magnesium tabsule, Tic Tac, Mentos, Mintie, Commercial Fiducial marker (IZI Multi-Modality, LifeHealthCare, Australia, included as a benchmark), Nail Polish, Coffee bean, Jelly baby sweet.

818x1036mm (72 x 72 DPI)

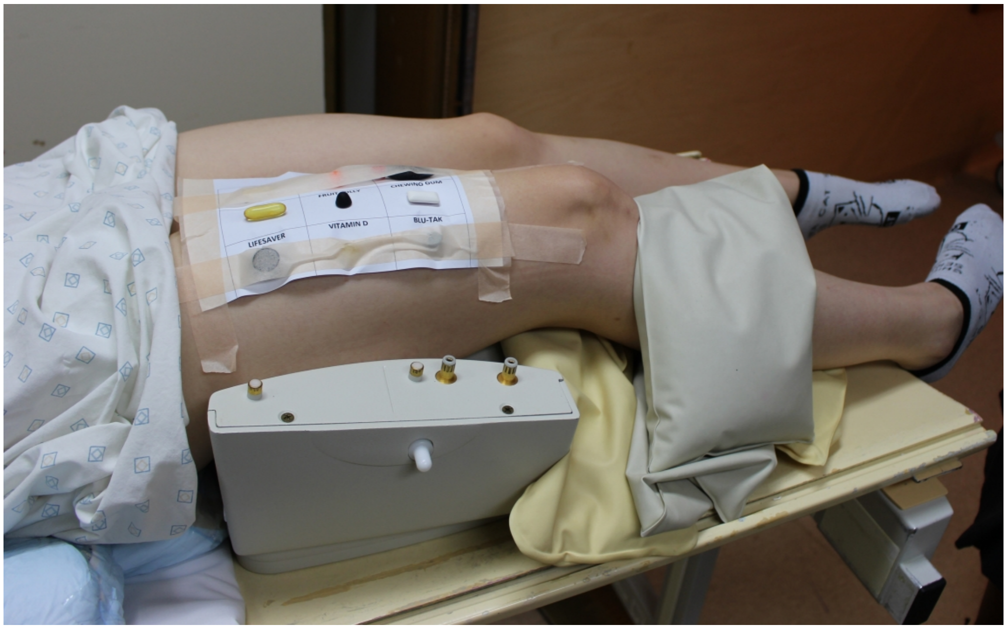

Figure 2. Markers were rigidly attached to the anterior surface of the thigh and the upper leg positioned in an RF coil. These images show the first nine items investigated.

1527x948mm (72 x 72 DPI)

**Sequence**	Proton Density	T1 Coronal	T1 3D	T2 Fat Sup	T2 3D
**Segmented Volume View of Anterior Thigh Surface**	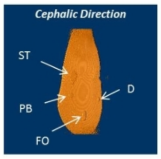	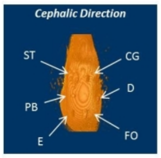	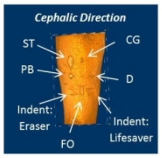	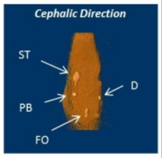	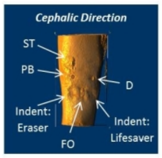
**Visible Markers**	• Fish Oil (FO)<br>• Vitamin D (D)<br>• Paint Ball (PB)<br>• Sushi Tube (ST)	• Fish Oil (FO)<br>• Vitamin D (D)<br>• Paint Ball (PB)<br>• Sushi Tube (ST)<br>• Chewing Gum (CG)<br>• Eraser (E)<br>*CG appeared to be visible on the 3D reconstruction but a 2D transverse slice at the level of CG did not demonstrate sufficient intensity contrast to differentiate the marker*	• Fish Oil (FO)<br>• Vitamin D (D)<br>• Paint Ball (PB)<br>• Sushi Tube (ST)<br>• Chewing Gum (CG)<br>*Indentations of the Lifesaver and Eraser were visible but the markers did not display*	• Fish Oil (FO)<br>• Vitamin D (D)<br>• Paint Ball (PB)<br>• Sushi Tube (ST)	• Fish Oil (FO)<br>• Vitamin D (D)<br>• Paint Ball (PB)<br>• Sushi Tube (ST)<br>*Indentation of the Lifesaver and Eraser are visible but marker does not display*

Figure 3. Segmented volume reconstructions of the anterior thigh surface, showing an example of markers that were visible for each MRI sequence for the first acquisition of 9 markers as shown in Figure 2.

319x140mm (150 x 150 DPI)

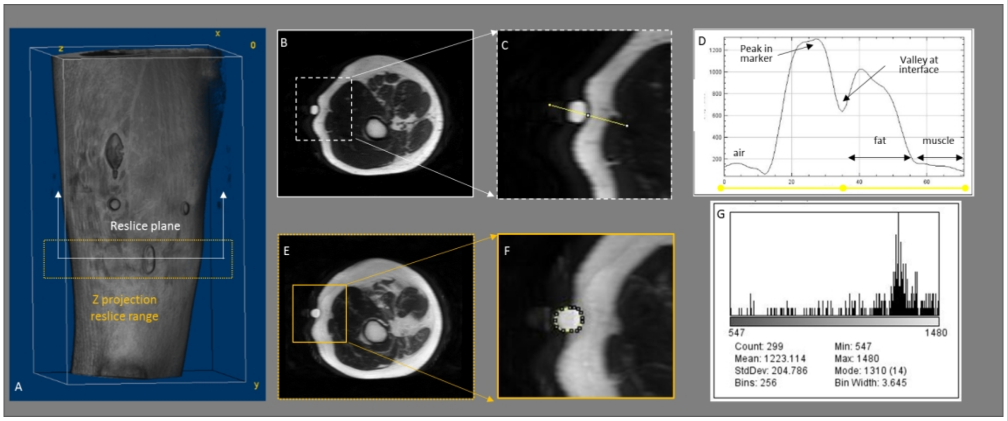

Figure 4. Workflow to measure relevant intensity parameters from each MRI dataset for each visible marker. A. 3D volume reconstruction of the T2, 3D sequence showing the transverse reslice plane (white line) and the range of z projection reslice plane (orange rectangle); B. Transverse reslice plane through the fish oil marker; C. Line selection across the marker (yellow unbroken line); D. Profile plot of intensity values along the line selection; E. Maximum intensity, transverse plane Z-projection of the fish oil capsule; F. Segmented selection tool demarcating the marker boundary (yellow broken line); G. Histogram detailing distribution of maximum intensities within the marker.

317x130mm (150 x 150 DPI)

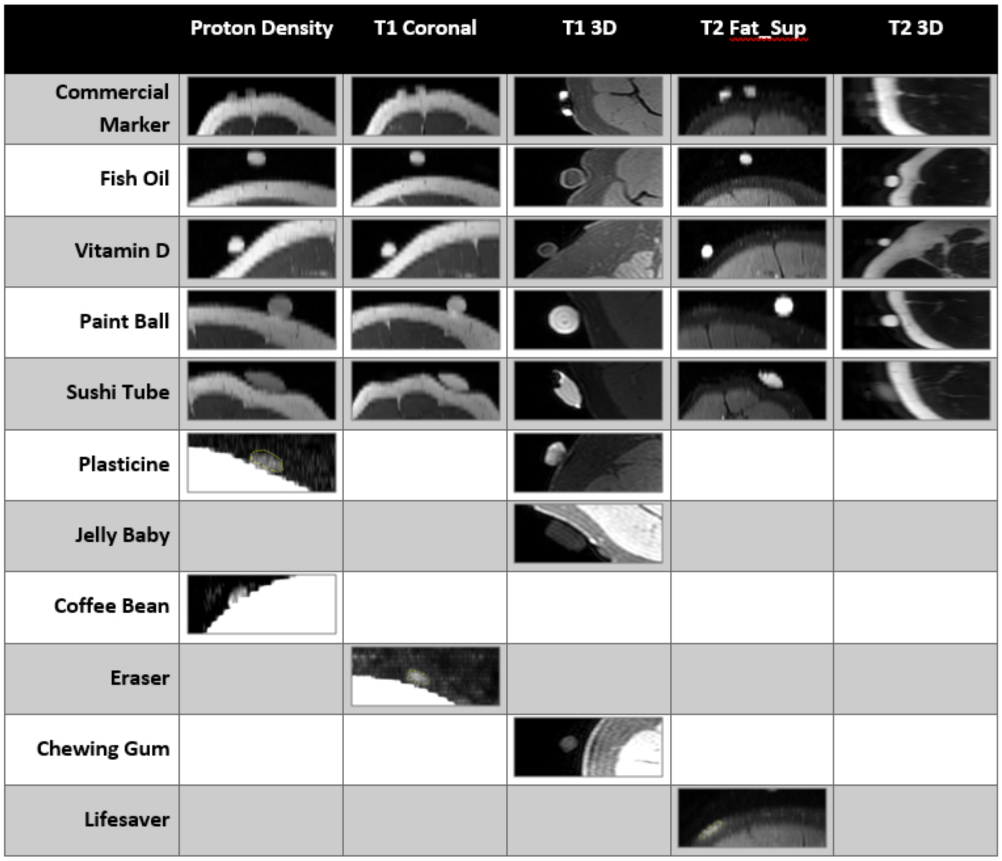

Figure 5: Reformatted transverse reslice images through the mid-height of all visible markers on the MR sequences analysed. Of the 18 markers, only 11 were visible on images from at least one of the MRI sequences – results for these 11 visible markers are shown. A blank box indicates that the marker tested was not clearly visible for a particular MR sequence.

201x173mm (96 x 96 DPI)

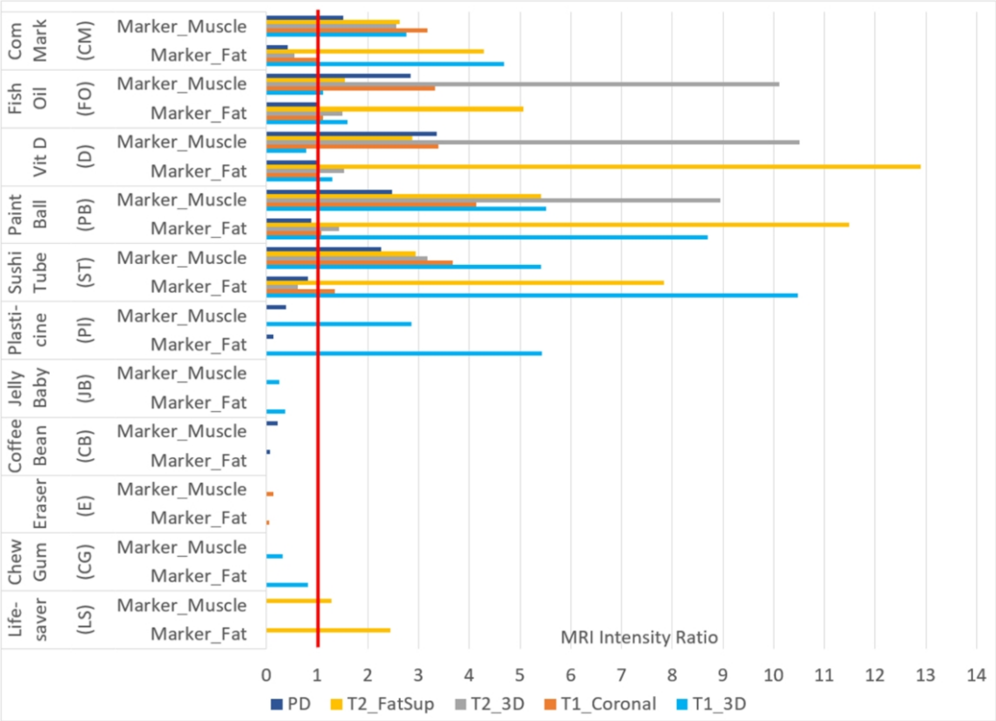

Figure 6: Intensity ratios for all visible markers. Marker_Muscle and Marker_Fat are the ratios between maximum marker intensity and mean intensity in the fat tissue and muscle tissue, respectively. Results are shown for the five MRI sequences: Proton Density (PD), T2 weighted fat-suppressed (T2_FatSup), 3D T2 weighted (T2_3D), T1 weighted coronal (T1_coronal) and 3D T1 weighted (T1_3D)

246x179mm (122 x 122 DPI)

Section & Topic	No	Item	Reported on page #
**TITLE OR ABSTRACT**		**Reliably Visible and Inexpensive surface Fiducial Marker options for Magnetic Resonance Imaging**	1
	1	Identification as a study of diagnostic accuracy using at least one measure of accuracy (such as sensitivity, specificity, predictive values, or AUC)	4
**ABSTRACT**			
	2	Structured summary of study design, methods, results, and conclusions (for specific guidance, see STARD for Abstracts)	2
**INTRODUCTION**			
	3	Scientific and clinical background, including the intended use and clinical role of the index test	3-4
	4	Study objectives and hypotheses	3-4
**METHODS**			
Study design	5	Whether data collection was planned before the index test and reference standard were performed (prospective study) or after (retrospective study)	Not applicable
Participants	6	Eligibility criteria	Not applicable
	7	On what basis potentially eligible participants were identified (such as symptoms, results from previous tests, inclusion in registry)	Not applicable
	8	Where and when potentially eligible participants were identified (setting, location and dates)	Not applicable
	9	Whether participants formed a consecutive, random or convenience series	Not applicable
Test methods	10a	Index test, in sufficient detail to allow replication	4-10
	10b	Reference standard, in sufficient detail to allow replication	4-10
	11	Rationale for choosing the reference standard (if alternatives exist)	4-10
	12a	Definition of and rationale for test positivity cut-offs or result categories of the index test, distinguishing pre-specified from exploratory	7-11
	12b	Definition of and rationale for test positivity cut-offs or result categories of the reference standard, distinguishing pre-specified from exploratory	7-11
	13a	Whether clinical information and reference standard results were available to the performers/readers of the index test	Not applicable
	13b	Whether clinical information and index test results were available to the assessors of the reference standard	Not applicable
Analysis	14	Methods for estimating or comparing measures of diagnostic accuracy	4-10
	15	How indeterminate index test or reference standard results were handled	Not applicable
	16	How missing data on the index test and reference standard were handled	Not applicable
	17	Any analyses of variability in diagnostic accuracy, distinguishing pre-specified from exploratory	Not applicable
	18	Intended sample size and how it was determined	Not applicable
**RESULTS**			
Participants	19	Flow of participants, using a diagram	Not applicable
	20	Baseline demographic and clinical characteristics of participants	Not applicable
	21a	Distribution of severity of disease in those with the target condition	Not applicable
	21b	Distribution of alternative diagnoses in those without the target condition	Not applicable
	22	Time interval and any clinical interventions between index test and reference standard	Not applicable
Test results	23	Cross tabulation of the index test results (or their distribution) by the results of the reference standard	11-16
	24	Estimates of diagnostic accuracy and their precision (such as 95% confidence intervals)	Not applicable
	25	Any adverse events from performing the index test or the reference standard	Not applicable
**DISCUSSION**			
	26	Study limitations, including sources of potential bias, statistical uncertainty, and generalisability	18
	27	Implications for practice, including the intended use and clinical role of the index test	15-17
**OTHER INFORMATION**			
	28	Registration number and name of registry	Not applicable
	29	Where the full study protocol can be accessed	4-10
	30	Sources of funding and other support; role of funders	17

For peer review only

cc BY SA

STARD 2015

AIM

STARD stands for “Standards for Reporting Diagnostic accuracy studies”. This list of items was developed to contribute to the completeness and transparency of reporting of diagnostic accuracy studies. Authors can use the list to write informative study reports. Editors and peer-reviewers can use it to evaluate whether the information has been included in manuscripts submitted for publication.

EXPLANATION

A **diagnostic accuracy study** evaluates the ability of one or more medical tests to correctly classify study participants as having a **target condition.** This can be a disease, a disease stage, response or benefit from therapy, or an event or condition in the future. A medical test can be an imaging procedure, a laboratory test, elements from history and physical examination, a combination of these, or any other method for collecting information about the current health status of a patient.

The test whose accuracy is evaluated is called **index test.** A study can evaluate the accuracy of one or more index tests. Evaluating the ability of a medical test to correctly classify patients is typically done by comparing the distribution of the index test results with those of the **reference standard**. The reference standard is the best available method for establishing the presence or absence of the target condition. An accuracy study can rely on one or more reference standards.

If test results are categorized as either positive or negative, the cross tabulation of the index test results against those of the reference standard can be used to estimate the **sensitivity** of the index test (the proportion of participants *with* the target condition who have a positive index test), and its **specificity** (the proportion *without* the target condition who have a negative index test). From this cross tabulation (sometimes referred to as the contingency or “2x2” table), several other accuracy statistics can be estimated, such as the positive and negative **predictive values** of the test. Confidence intervals around estimates of accuracy can then be calculated to quantify the statistical **precision** of the measurements.

If the index test results can take more than two values, categorization of test results as positive or negative requires a **test positivity cut-off**. When multiple such cut-offs can be defined, authors can report a receiver operating characteristic (ROC) curve which graphically represents the combination of sensitivity and specificity for each possible test positivity cut-off. The **area under the ROC curve** informs in a single numerical value about the overall diagnostic accuracy of the index test.

The **intended use** of a medical test can be diagnosis, screening, staging, monitoring, surveillance, prediction or prognosis. The **clinical role** of a test explains its position relative to existing tests in the clinical pathway. A replacement test, for example, replaces an existing test. A triage test is used before an existing test; an add-on test is used after an existing test.

Besides diagnostic accuracy, several other outcomes and statistics may be relevant in the evaluation of medical tests. Medical tests can also be used to classify patients for purposes other than diagnosis, such as staging or prognosis. The STARD list was not explicitly developed for these other outcomes, statistics, and study types, although most STARD items would still apply.

DEVELOPMENT

This STARD list was released in 2015. The 30 items were identified by an international expert group of methodologists, researchers, and editors. The guiding principle in the development of STARD was to select items that, when reported, would help readers to judge the potential for bias in the study, to appraise the applicability of the study findings and the validity of conclusions and recommendations. The list represents an update of the first version, which was published in 2003.

More information can be found on http://www.equator-network.org/reporting-guidelines/stard.

For peer review only

cc BY SA

BMJ Open

Determining a Reliably Visible and Inexpensive Surface Fiducial Marker for Use in Magnetic Resonance Imaging: a research study in a busy Australian Radiology Department.

Journal:	*BMJ Open*
Manuscript ID	bmjopen-2018-027020.R3
Article Type:	Research
Date Submitted by the Author:	11-Jun-2019
Complete List of Authors:	Izatt, Maree; Queensland University of Technology, Biomechanics & Spine Research Group<br>Lees, Deborah; Queensland University of Technology, Biomechanics & Spine Research Group<br>Mills, Susan; Mater Misericordiae Brisbane Ltd, Mater Medical Imaging<br>Grant, Caroline; Queensland University of Technology, Biomechanics & Spine Research Group<br>Little, Judith; Queensland University of Technology, Biomechanics & Spine Research Group
**Primary Subject Heading**:	Qualitative research
Secondary Subject Heading:	Diagnostics, Paediatrics, Patient-centred medicine, Public health, Radiology and imaging
Keywords:	Magnetic resonance imaging < RADIOLOGY & IMAGING, MRI, fiducial marker, surface marker, MRI sequence, marker visibility

SCHOLARONE™
Manuscripts

Title:

Determining a Reliably Visible and Inexpensive Surface Fiducial Marker for Use in Magnetic Resonance Imaging: a research study in a busy Australian Radiology Department.

Authors:

Maree T. Izatt[1], B.Phty m.izatt@qut.edu.au
Project Coordinator, (ORCID ID: 0000-0002-4926-3077)

Deborah Lees[1], M.Tech (Chiro), MBBS, FRCS (Trauma & Orth) deborah.lees@health.qld.gov.au
Spine Fellow, Orthopaedic Surgeon

Susan Mills[2], B.App.Sci (MI) susan.mills@mater.org.au
MRI Unit Manager

Caroline A. Grant[1], PhD, BEng ca.grant@qut.edu.au
Postdoctoral Research Fellow, (ORCID ID: 0000-0002-5128-9903)

J. Paige Little[1], PhD, BEng j2.little@qut.edu.au
Senior Research Fellow, (ORCID ID: 0000-0001-8377-5343)

Affiliations:

[1] Biomechanics and Spine Research Group, Institute of Health and Biomedical Innovation at Centre for Children's Health Research, Queensland University of Technology, Brisbane, Australia

[2] Mater Medical Imaging, Mater Misericordiae Ltd, Brisbane, Australia

Corresponding Author:

Dr J P Little Email: j2.little@qut.edu.au; Phone: +61 7 3069 7325

For peer review only

For peer review only - http://bmjopen.bmj.com/site/about/guidelines.xhtml

Structured Abstract

Objectives

Single use commercial surface fiducial markers are used in clinical imaging for a variety of applications. The current study sought to find a new, reliably visible, easily sourced and inexpensive fiducial marker alternative for use with magnetic resonance imaging (MRI).

Design

Five commonly requested MRI sequences were determined (3D T1-weighted, T1 coronal, 3D T2-weighted, T2 fat suppressed, Proton density), to examine the visibility of 18 items (including a commercial fiducial marker).

Setting

Clinical 3T MRI scanner in an Australian Tertiary Hospital and an Australian University Biomedical Engineering research group

Interventions

18 marker alternatives were scanned using five common MRI sequences. Images were reformatted to obtain both an image through the mid-height of each marker and a maximum intensity z-projection image over the volume of the marker. Variations in marker intensity were profiled across each visible marker and a visibility rating defined.

Main outcome measures

Outcome measures were based on quantitative assessment of a clear intensity contrast ratio between the marker and the adjacent tissue and a qualitative assessment of visibility via a 3-point scale.

Results

The fish oil capsule, vitamin D capsule, paint ball pellet, soy sauce sushi tube and commercial markers were typically visible to a high quality on all the imaging sequences and demonstrated a clear differential in intensity contrast against the adjacent tissue. Other common items, such as plasticine 'play doh' and a soft 'Jelly baby' sweet were surprise candidates, demonstrating high quality visibility and intensity contrast for the 3D T1-weighted sequence.

Conclusions

Depending on the basis for referral and MRI sequence chosen, four alternative fiducial markers were determined to be inexpensive, easily sourced and consistently visible. Of these, the Vitamin D capsule provided an excellent balance between availability, size, cost, usability and quality of the visualised marker for all the commonly used MRI sequences analysed.

For peer review only

Strengths and Limitations

- This manuscript is the first to test for easily sourced and economical items to find reliably visible surface fiducial marker options for common MRI sequences

- Scanning the fiducial markers options on the thigh of a healthy adult female provides a superior assessment of marker visibility than analyses that use a saline phantom object.

- The study presented both quantitative and qualitative analyses of marker visibility, thus providing a more practical assessment of the ability of the radiographer/investigator to visualise the marker.

- Five commonly utilised MRI sequences were investigated to provide the most relevant alternative marker for the majority of clinical MR imaging but there may be other MRI sequences of interest that were not included in the current study.

- The markers tested in the current study were a limited sample of convenience and included only one commercial fiducial marker.

Keywords
Magnetic resonance imaging, MRI, fiducial marker, surface marker, MRI sequence, marker visibility, marker intensity, low cost marker, inexpensive marker

For peer review only

Introduction:

Fiducial markers used in the context of clinical medicine may be implanted in the body or placed on the surface and are also utilised in research for a variety of applications. Placed in the field of view, they display as distinct regions of high intensity to assist in pinpointing specific anatomical landmarks or pathologies on the acquired clinical images. Surface markers placed on a patient's skin can also provide a frame of reference for registration of medical images acquired using multiple imaging modalities, such as photography, computed tomography (CT) and magnetic resonance imaging (MRI) performed for concomitant pathologies involving multiple specialities.(1)

In radiotherapy, fiducial markers are increasingly being used to identify the tumour site, permitting image registration and assisting with guidance for treatment planning.(2-4) Oil-based surface markers have been shown to compare favourably to solid markers in terms of their contrast to noise ratio, resulting in excellent visibility.(4) Prostate marker studies also revealed that implanted titanium seeds left star/streak artefacts on CT imaging and could not be accurately localised on MR due to negative contrast (black holes).(5-7) Fiducial markers also perform a valuable role in guiding anatomical identification in musculoskeletal studies, particularly kinematic gait analyses where the location of surface markers on both mathematical gait models and the accompanying 3D MRI images provides valuable subject-specific simulation parameters.(8)

Commercially available fiducial surface markers (i.e. non-implantable) are available in a range of sizes and shapes for use in the wide variety of clinical imaging applications. They are self-adhesive and have a low, flat profile, making them comfortable for the patient. While these markers provide excellent utility, they present a substantial expense to radiology departments, with a review of the current markets showing prices ranging from \$420 to \$600 USD for a box of 100 (MR Spots® and IZI multi-modality, excluding shipping and tax). Being a single use product they are a considerable expense to the running costs of imaging departments or research teams utilizing MRI and requiring numerous markers.

A review of the literature to find materials that are visible in MRI revealed a paucity of publications. The majority of papers examining substance visibility were focused primarily on locating a variety of penetrating or ingested foreign bodies and just a few papers explored implanted marker options for tumour boundary markers prior to excision or radiotherapy planning.(3, 5-7, 9) Foreign bodies that had been located, ranged in material from fish and chicken bones, batteries, plastics, coins, metal and wood splinters (10-13) and it should be noted that some materials were missed even with the high sensitivity of modern MRI and CT.(14, 15) Pattamapospong *et al* (16) published results regarding the visibility of a number of substances on X-ray, CT and MRI and demonstrated a 100% specificity but only 58% sensitivity for fresh wood, dry wood, glass, plastic and porcelain using MRI. Wax crayons have proven to be easily visible on both CT and MRI with high attenuation noted on CT and a signal void on both T1 and T2 MR images. Interestingly, it was noted that crayon colour influenced the degree of visibility. Different pigments used in crayons and in paints resulted in distinctive colours as well as MRI appearance.(17) Injectable facial fillers (hyaluronic acid, collagen and polyalkylimide-polyacrylamide hydrogels), silicone and calcium hydroxyapatite have also been well visualised on MRI but can be confused with malignant features, so are not considered good marker substances.(18) Metal is well visualised on many modalities (10) but due to its ferrous properties, is contra-indicated in the field of MRI. Metal, even if MR conditional, results in significant image attenuation and artefact. For these reasons, an iron tablet as a makeshift marker may be well

For peer review only - http://bmjopen.bmj.com/site/about/guidelines.xhtml

visualised but the resulting artefact renders it unacceptable. Studies looking at the visibility of plastic, stone, glass and graphite revealed that both glass and plastic foreign bodies are not consistently visible on MRI or plain radiographs; despite showing up well on CT.(16, 19)

A review of the physics of MRI suggested materials with high water or fat content were likely items that would be readily detectable. Fiducial markers should be easily identifiable and clearly visible on clinical images, allowing the target anatomy or volume to be clearly and completely visualised. Therefore a critical objective of this study was to assess the validity of various 'everyday' items, which could be easily and economically sourced, and provide a reliably visible fiducial marker as an alternative to the comparatively expensive, single use commercial fiducial markers. We hypothesised that an inexpensive, readily sourced, robust surface fiducial marker could be isolated, that consistently demonstrated at least the same level of visibility as a commercial fiducial marker, when viewed on the most commonly performed MRI sequences.

Methods:

Seventeen everyday items and a commercial fiducial marker were selected for analysis, either from the literature or anecdotal reports (Figure 1). The important considerations relating to the selection of a suitable surrogate for commercial fiducial markers were identified and are outlined in Table 1. In addition to the commercial fiducial marker, two of these items had been used in our local hospital medical imaging department; the paint ball for MR scans in a pilot research project, and the fish oil capsule which was in regular use to avoid using a commercial marker whenever possible. While these two non-commercial markers provided excellent results in terms of intensity contrast and visualisation on MR images, to date a definitive comparison of the image contrast provided by these items has not been undertaken and these alternatives did not meet all the desired requirements of the ideal alternative fiducial marker.

For peer review only

Table 1. Important considerations relating to the choice of a suitable fiducial marker for MR imaging

Factors for consideration	
**Accessibility**	Easily acquired, readily replaced, and preferably locally sourced without high shipment costs or transit time
**Use**	Sufficiently robust to tolerate body weight if placed between the participant and scanner bed<br>Suitably compliant or flat to ensure minimal discomfort to the patient if marker is in a load bearing location (e.g. Posterior superior iliac spine for a supine MRI)
**Biochemistry**	Chemically inert, hypoallergenic
**Economy**	Low acquisition cost per marker given markers are single use items
**Medical Physics**	Sufficient hydrogen content to permit high intensity contrast to surrounding tissue
**Fixation**	Reliably affixed to the patient's external anatomy

r revi

Imaging Details:

Table 2. MRI sequence details

	Imaging Protocol		Acquisition plane	Field of View (FOV) (mm)	Repetition time (TR) / Echo time (TE) (msec)	Resolution (Pixel width x height x slice spacing, mm)
**1**	Proton density (PD)		Coronal	300 x 300 x 44	2,435/35	0.1465 x 0.1465 x 2.2
**2**	T1 weighted, Spin echo		Coronal	300 x 300 x 44	661/10	0.1465 x 0.1465 x 2.2
**3**	T1 weighted, 3D Fast field echo (FFE)		Sagittal	300 x 300 x 175	25/1.4	0.469 x 0.469 x 0.5
**4**	T2 weighted, fat suppressed		Coronal	300 x 300 x 44	4,448/65	0.195 x 0.195 x 2.2
**5**	T2 weighted, 3D		Sagittal	300 x 300 x 175	2,473/72	0.469 x 0.469 x 0.5

The selection of items underwent MR scanning in a clinical medical imaging department using a 3-T Philips Achieva MRI scanner. Scanning parameters vary between different machine manufacturers and even between different scanner models from the same manufacturer. However, this particular scanner was selected as it services a busy hospital radiology department in a metropolitan city, and thus, provides a broad range of exploratory and treatment-based MRI scanning services. Further to this, the 3-T magnet strength is a typical specification for scanners in tertiary care facilities in Australia and internationally.

Following the advice from the senior MR radiographers and a survey of local surgeons, five different sequences were performed (Table 2) to cover the breadth of MR imaging studies typically performed with a requirement for inclusion of surface markers. All tested markers were attached to the anterior thigh of a healthy female participant, aged 27 years, who had provided informed consent. A radiofrequency coil (8-channel knee coil) was positioned beneath the thigh and the markers attached to the anterior thigh surface (Figure 1). Due to physical size constraints, the marker selection was attached to the volunteer's leg in two separate acquisitions. In all the MR sequences detailed in Table 2, the scanning parameters were constant for both acquisitions.

Images were saved in Digital Imaging and Communications in Medicine (DICOM) format and analysed using ImageJ (U. S. National Institute of Health Open-source software, Maryland, USA; https://imagej.nih.gov/ij/). A similar process was utilised to assess the results from each of the five different imaging sequences (Table 2) and involved first viewing the 3D stack as a segmented volume using the ImageJ Plugin, 'Volume Viewer 2.01' (K U Barthel, Internationale Medieninformatik, HTW Berlin, Germany) (Figure 3).

In the first instance, a qualitative evaluation of the marker visibility was carried out to subjectively assess the relative ease with which each marker could be visualised and the marker edges demarcated. Viewing the data as a 3D volume permitted an initial assessment of which markers exhibited an intensity that was visible on MR images (Figure 3). If the marker was visible, the visibility was rated on a 3-point Likert scale with 3 representing 'very clear', 2 representing 'edges visible but fuzzy' and 1 representing 'not easily visible'. If a visibility rating of one was observed, this low rating suggested that while the marker was discernibly visible on the MR images, the quality of visibility was poor and the marker would not be recommended for use in clinical scanning using that particular MR sequence. Using this scale, a mean visibility rating across the five MRI sequences was calculated for each fiducial marker. While a mean visibility rating >0 indicated that the marker could be seen on at least one of the MRI sequences, a mean visibility rating greater than 2 was preferable as it indicated a consistently good quality visibility on all of the MRI sequences performed.

In all cases, MR images were reformatted and processed as 16-bit images, with an intensity range from 0 to 65,536. A quantitative evaluation of the marker quality was carried out to assess the visibility, artefacts and distortion of marker boundaries created by each item on each MRI sequence. This evaluation was carried out using the steps outlined below and shown visually in Figure 4.

1. Reformatted Image Stack: The DICOM stacks were reformatted to create a sequential stack of transverse reslice images, with consideration of the relevant pixel and slice spacing for each dataset (Table 2).
2. Intensity Profile Plot: Using the 3D volume reconstructions for each MRI sequence as a reference (Figure 3, Figure 4 A), the reformatted transverse reslice through the mid-height of each marker was located (Figure 4 B). A line selection (Figure 4 C) was drawn through the marker, such that

For peer review only - http://bmjopen.bmj.com/site/about/guidelines.xhtml

the mid-point on the line was positioned at the interface between the marker and the skin and the endpoints were located in air and muscle tissue, respectively. The intensity profile along this line selection (Figure 4 D) was used to objectively compare the marker signal and visibility. The peak intensity in the marker, minimum intensity at the interface between the marker and tissue, mean intensity in the fat tissue and mean intensity in the muscle tissue were recorded from the profile.

3. Maximum Intensity Z-projection: A two-dimensional (2D) image with each pixel intensity equivalent to the maximum intensity along a Z trajectory over a given range of stack images was created (Figure 4 E) The image range was defined to encompass the upper and lower limits of the marker, as viewed on the 3D volume reconstruction (Figure 4 A, orange broken line).
4. Intensity Histogram and Maximum Marker Intensity: A segmented line tool (Figure 4 F) was used to demarcate the outer profile of the marker on the 2D Z-projection image (Figure 4 E). A histogram showing the distribution of maximum intensities within this profile for the marker was used to record the maximum and mean marker intensity (Figure 4 G).

In some instances (Figure 3: T1_3D – Chewing Gum; T1 Coronal – Eraser) the intensity range exhibited by the marker was very low in comparison to the background intensity, making the marker boundaries difficult to differentiate from the surrounding regions. However, these markers were still included in the analysis as it was possible to create a profile plot (Figure 4 D) on a transverse plane through the mid-height of the marker, with a measurable intensity gradient across the marker.

Assessment of Intensity Values:

In order to objectively assess whether the markers provided an acceptable visualisation on the images produced for each MR sequence analysed, three criteria were defined to assess the results. It was expected that the markers should; a) Provide a pixel intensity that was sufficiently above the background intensity of air to result in a clear visual contrast; b) Demonstrate a pixel intensity ratio at the interface between the marker and adjacent epidermis/fat tissue (i.e. peak marker intensity divided by interface intensity/mean fat tissue intensity) greater than 1; and c) Demonstrate a pixel intensity ratio between the marker and the muscle tissue (i.e. peak marker intensity divided by mean muscle intensity) greater than 1.

The ratios were intended to provide a numeric value that aligned with the perceptual and visual decision making process a person undertakes when determining whether a marker is visualised at an acceptable quality. To indicate the quality of marker visualisation, this ratio of unity was defined after viewing all markers over the five MRI sequences and in combination with the visual assessment of visibility rating.

Depending on the MRI sequence, the fat and muscle tissue adjacent to the marker displayed over different intensity ranges. The ratio between the fat or muscle tissue and marker intensity was considered critical to be able to differentiate the marker from its surroundings. For this reason the intensity ratio was considered of more importance than the absolute value of the pixel intensity.

Patient and Public Involvement:

This research was done without patient or public involvement as an aspect of a larger study yet to be performed. Participants in the larger study will be asked to be involved in the design of the subjective assessment methods.

For peer review only - http://bmjopen.bmj.com/site/about/guidelines.xhtml

Results

Of the 18 markers investigated, five were consistently visible for all imaging sequences, but this visibility was of differing quality (Table 3). The Fish Oil (FO), Vitamin D (D), Paintball (PB), Soy Sauce Sushi Tube (ST) and Commercial Marker (CM) were typically visible to a high quality (i.e. rating 2 or 3) for all sequences (Table 3). A visibility rating of 1 on only one of the MR sequences was observed for the Eraser (E), Chewing Gum (CG), Lifesaver (LS), and Coffee Bean (CB), indicating the quality of visibility was poor. Surprisingly, the Pl marker was visible to a high quality (rating 3) for the T1_3D sequence, but had little (rating = 1 for PD sequence) or no visibility for the other sequences. Similarly, the Jelly Baby was also remarkable in being well visualised on the T1_3D sequence with a rating of 2, but had no visibility scored for the other MR sequences.

A mean visibility score greater than 2 was observed for the PB, FO, D and CM markers (Table 3).

For peer review only

Table 3. Visibility rating for each marker on each MRI sequence. If not visible, the marker rating = 0. If visible, marker visibility rating was either 1 ('visible, but not easily'), 2 ('edges visible but fuzzy') or 3 ('very clear').

	Proton Density	T1 Coronal	T1_3D	T2 Fat_Sup	T2_3D	MEAN RATING
**Commercial Marker**	2	2	3	3	2	2.4
**Fish Oil**	2	2	3	2	3	2.4
**Vitamin D**	2	2	3	3	3	2.6
**Paint Ball**	2	2	3	3	3	2.6
**Soy Sauce Sushi Tube**	2	2	3	2	1	2.0
**Plasticine**	1	0	3	0	0	0.8
**Jelly Baby**	0	0	2	0	0	0.4
**Coffee Bean**	1	0	0	0	0	0.2
**Eraser**	0	1	0	0	0	0.2
**Chewing Gum**	0	0	1	0	0	0.2
**Lifesaver**	0	0	0	1	0	0.2
**Fruit Lolly**	0	0	0	0	0	0
**BluTak**	0	0	0	0	0	0
**Magnesium**	0	0	0	0	0	0
**TicTac**	0	0	0	0	0	0
**Mentos**	0	0	0	0	0	0
**Mintie**	0	0	0	0	0	0
**Nail Polish**	0	0	0	0	0	0

Figure 5 shows a summary of the visible markers on the five MRI sequences. In each case, the intensity range for the image was selected such that the intensity contrast within the image could best display the marker. These images do not include image filtering or contrast enhancement. Viewing the reslice images in Figure 5 in combination with the visibility ratings in Table 3 demonstrated that the best quality marker visibility for the T1_3D, T1 coronal, T2 Fat_Sup and Proton Density sequences was the PB, ST, FO and D; and for the T2_3D sequence was the PB, FO and D. The CM was best visualised on the T1_3D and T2 Fat_Sup sequences.

For peer review only - http://bmjopen.bmj.com/site/about/guidelines.xhtml

Intensity ratios between the maximum marker intensity and the mean intensity in either the fat tissue or muscle tissue are shown in Figure 6.

For the PD sequence, while the CM, FO, D, PB and ST markers were easily visible (Figure 5, Figure 6), the marker intensity was numerically similar to the fat tissue, resulting in marker-to-fat ratios near or below one. The PL and CB were less easily differentiated from the nearby fat tissue (Figure 6).

For the T2 fat suppressed sequence, there was a distinct differential between the maximum intensity in the marker and the mean intensity in the fat tissue, making all observable markers (CM, FO, D, PB, ST and LS) clearly visible (Figure 6).

The commercial marker (CM) was not clearly visualised for the 3D T2-weighted sequence (Table 3, Figure 5), and had a marker-to-fat ratio <1 (Figure 6). While the ST tended to result in good visibility (Table 3, Figure 5), for the 3D T2-weighted sequence the marker intensity was less than the muscle tissue and not easily differentiated from the background air intensity. Aside from this, the 3D T2-weighted sequence showed an intensity ratio >>1 and thus clear intensity contrast for the marker-to-muscle ratio (>1) and marker-to-fat ratio, for all markers.

While the eraser (E) was visible on the T1 coronal sequence, the intensity ratios suggested that the intensity contrast between the marker and adjacent muscle/fat was not of a good quality (Table 3, Figure 5). This was supported by the observed intensity ratio <1 for both the marker-to-muscle and marker-to-fat ratios (Figure 6). For the other visible markers, the intensity ratios were all ≥ 1.
With the exception of the chewing gum (CG) and jelly baby (JB), the intensity ratios for marker-to-fat were all >1, for the 3D_T1-weighted sequence. In the case of the PB and ST marker, since these were comprised of a non-oil based substance, the intensity contrast and marker-to-fat/marker-to-muscle intensity ratios were >>1 (Figure 6). These latter two markers also resulted in a high visibility rating (Table 3, Figure 5).

Discussion

Fiducial markers are used for calibration of the imaging equipment, providing a reference measurement for templating software, locating specific boundaries for radiotherapy planning, orienting an image and very importantly, to identify a specific area of interest in the case of diagnostic queries. It is thus essential to have a marker that is detectable and clearly discernible from bone and soft tissues when placed in the visual field for a number of common MR sequences. Single use, commercially available fiducial markers make up a significant component of the imaging department's outgoings and in an attempt to reduce costs, the current study was a practical exercise designed to image a number of commonly available everyday items to assess their suitability to function as an effective surface fiducial MRI marker substitute. The current study aimed to provide additional marker options useful for personalised musculoskeletal kinematic models as well as for clinical pathologies and research projects utilizing MRI.

A single use, commercially available surface fiducial marker was evaluated in this study alongside seventeen potential alternatives and its performance was surprisingly less impressive than some of the more common and affordable items tested. The commercial marker is considered prohibitively expensive (AUD$ 6 to 10 per marker) and is therefore used selectively for specific neurosurgical

For peer review only - http://bmjopen.bmj.com/site/about/guidelines.xhtml

imaging needs only. Makeshift markers of various types have been anecdotally trialled in clinical radiology departments over the years. Discussions with our hospital imaging staff revealed that almonds, Vitamin E capsules and condiment packets had previously been trialled. Packaging rupture was a risk and unfortunately these makeshift markers caused issues with patient comfort, and inconsistent visibility on imaging.

In the current study, the FO, PB and D markers were all clearly visible on each of the five sequences and typically demonstrated well-defined intensity contrast with the adjacent fat/tissue. On the basis of intensity contrast, these three markers would be recommended in place of a commercial marker (CM) for use as an external fiducial marker.

However, as noted in Table 1, in addition to MRI visibility of the marker, consideration of the cost, accessibility and useability of the surrogate marker are of equal importance. In light of this, the PB is less accessible, requiring specific online ordering or purchase at custom stores. Additionally concerns remain with regards to the risk of damage to equipment and property if the paint ball ruptured as the pellets are designed to burst at low force to prevent injury during game play. The FO, while clearly visualised and sourced, are large in size making them difficult to use accurately for small pathologies or on extremities. They also bring with them the risk of capsule rupture and the release of a pungent odour.

Oil-based capsules are commonly used as a commercial marker alternative (20). Vitamin D capsules are economically purchased (cost of AUD$36 for 400 capsules), small in size, readily available and bring no detrimental outcomes should they rupture. Vitamin D provided a bright MRI marker virtually identical to the visibility of the FO capsule, but was significantly smaller in size (~13mm long), making it more appealing when markers are required in imaging of the extremities, face or small pathologies. Anecdotally, the authors of this study have since used Vitamin D capsules routinely for supine musculoskeletal studies of the spine requiring multiple markers affixed throughout extended sessions in the MR scanner and have not had any degradation or rupture, with excellent visibility of these markers observed on the resulting MR images.

While the Vitamin D capsule was the final fiducial marker of choice, a discussion of other markers that were well visualised on some sequences is relevant given the aim of this study. Fluids are more readily identifiable on MRI and lipid-based markers have proven to be consistently reliable.(9, 21) The capped sushi soy sauce tubes tested in the current study were reliably visible on all the MRI sequences analysed and were considered by the MR radiographers as a useful alternative marker candidate. They suggested one or more of the fish-shaped tubes could act as a type of "pointer" to highlight a small lesion or point of interest without obscuring it. It was also considered sufficiently small to not compress or cover the relevant anatomy.

Performing successful MRI on children can be challenging and may require a creative approach, especially in the younger age groups. Children who have experienced a lot of medical interventions can be fearful of any and all medical procedures. Therefore we suggest it may be a great advantage to be able to produce a 'jelly baby' sweet to apply as a fiducial imaging marker when a T1_3D sequence was required, or alternatively a fish shaped soy sauce tube or Vitamin D capsule if this particular sequence was not appropriate.

Depending on the reason for imaging and the specific pathology, different MRI sequences are chosen to provide well visualised tissue contrast. For example, to demarcate the location of a tumour, a 3D T1-weighted/T2-weighted or T1 Fat Suppressed protocol would be considered more

For peer review only - http://bmjopen.bmj.com/site/about/guidelines.xhtml

appropriate while for exploratory imaging relating to tissue infection, a T1 coronal or T2 Fat Suppressed protocol is preferable. Conversely, when MR imaging is requested for musculoskeletal conditions, a Proton Density sequence is the preferred protocol to provide high intensity contrast in both fat and fluid in the bone. As such, this study provides evidence that there may not be one marker that is best suited to all imaging sequences and individual markers may provide good quality results for some but not all sequences (Table 3, Figure 5).

Regarding study limitations, while a single commercial fiducial marker was analysed in the current study, it was the brand used in our clinical imaging facility making it the most relevant marker to examine. A single human participant was used and only the thigh region was scanned, but we considered this to be superior to a saline phantom. Marker visibility was analysed by a single observer but typically in the clinical setting a single examiner views images to make judgements on visible anatomy. We acknowledge that some older clinical magnets may have a strength as low as 1.5T, however, we feel these findings relating to the FO, PB and D markers are still relevant for a lower strength magnet. Furthermore, there are a range of other additional MRI sequences that may be relevant for investigation of an alternative fiducial marker (e.g. Dixon imaging), however, the five sequences chosen in the current study were on the advice of the collaborating Radiology Department and following a survey of local surgeons of the most commonly requested MRI sequences used for diagnostics and anatomical investigations.

For peer review only - http://bmjopen.bmj.com/site/about/guidelines.xhtml

Figure Titles

Figure 1. 18 items investigated as surrogates for the commercially available fiducial markers – Eraser, Paint Ball, Sushi Tube, Fish Oil capsule, Fruit lolly, Chewing gum, Lifesaver, Vitamin D, Blu-Tak, Plasticine, Magnesium tabsule, Tic Tac, Mentos, Mintie, Commercial Fiducial marker (IZI Multi-Modality, LifeHealthCare, Australia, included as a benchmark), Nail Polish, Coffee bean, Jelly baby sweet.

Figure 2. Markers were rigidly attached to the anterior surface of the thigh and the upper leg positioned in an RF coil. These images show the first nine items investigated.

Figure 3. Segmented volume reconstructions of the anterior thigh surface, showing an example of markers that were visible for each MRI sequence for the first acquisition of 9 markers as shown in Figure 2.

Figure 4. Workflow to measure relevant intensity parameters from each MRI dataset for each visible marker. A. 3D volume reconstruction of the T2, 3D sequence showing the transverse reslice plane (white line) and the range of z projection reslice plane (orange rectangle); B. Transverse reslice plane through the fish oil marker; C. Line selection across the marker (yellow unbroken line); D. Profile plot of intensity values along the line selection; E. Maximum intensity, transverse plane Z-projection of the fish oil capsule; F. Segmented selection tool demarcating the marker boundary (yellow broken line); G. Histogram detailing distribution of maximum intensities within the marker.

Figure 5: Reformatted transverse reslice images through the mid-height of all visible markers on the MR sequences analysed. Of the 18 markers, only 11 were visible on images from at least one of the MRI sequences – results for these 11 visible markers are shown. A blank box indicates that the marker tested was not clearly visible for a particular MR sequence.

Figure 6: Intensity ratios for all visible markers. Marker_Muscle and Marker_Fat are the ratios between maximum marker intensity and mean intensity in the fat tissue and muscle tissue, respectively. Results are shown for the five MRI sequences: Proton Density (PD), T2 weighted fat-suppressed (T2_FatSup), 3D T2 weighted (T2_3D), T1 weighted coronal (T1_coronal) and 3D T1 weighted (T1_3D)

For peer review only

For peer review only - http://bmjopen.bmj.com/site/about/guidelines.xhtml

Competing Interests:

All authors have completed the ICMJE uniform disclosure form (available on request from the corresponding author) and declare: no support from any organisation for the submitted work; no financial relationships with any organisations that might have an interest in the submitted work in the previous three years; no other relationships or activities that could appear to have influenced the submitted work.

Funding Statement:

The Queensland University of Technology, Brisbane, Australia provided the funding for the Article Processing Charges levied for publication of this manuscript

Contributors:

MTI, CAG, JPL were involved in the conception, design and conduct of the research project, interpretation of the data and preparation of the manuscript. JPL performed the data analysis and edited the manuscript. DL wrote the first draft of the introduction and discussion. SM assisted with the design of the project, performed the imaging and assisted with the interpretation of the data and results. All authors were involved in the drafting and revising of the manuscript and gave final approval of the final version. All authors meet the ICMJE criteria for authorship. MTI and JPL are guarantors for the work and conduct of the study.

Transparency Declaration:

MTI and JPL affirm that the manuscript is an honest, accurate, and transparent account of the study being reported; that no important aspects of the study have been omitted; and that any discrepancies from the study as originally planned (and, if relevant) have been explained.

Ethical Approval:

Ethical approval was obtained from Queensland University of Technology Human Research Ethics Committee (approval # 1700000335)

Copyright:

The Corresponding Author has the right to grant on behalf of all authors and does grant on behalf of all authors, an exclusive licence on a worldwide basis to the BMJ Publishing Group Ltd to permit this article (if accepted) to be published in BMJ editions and any other BMJPGL products and sub-licences such use and exploit all subsidiary rights, as set out in our licence.

Data Sharing:

Data may be made available via the author's Institutional Research Data Storage Service (RDSS) after the paper has been published. Through the RDSS, non-institutional researchers may be granted access to this data after making a request to author (Dr J P Little, j2.little@qut.edu.au) and owner of the RDSS entry.

For peer review only - http://bmjopen.bmj.com/site/about/guidelines.xhtml

For peer review only

For peer review only - http://bmjopen.bmj.com/site/about/guidelines.xhtml

References

1. Tanaka O, Komeda H, Hirose S, Taniguchi T, Ono K, Matsuo M. Visibility of an iron-containing fiducial marker in magnetic resonance imaging for high-precision external beam prostate radiotherapy. Asia Pac J Clin Oncol. 2018;14(5):e405-e11.
2. Chan MF, Cohen GN, Deasy JO. Qualitative evaluation of fiducial markers for radiotherapy imaging. Technol Cancer Res Treat. 2015;14(3):298-304.
3. Schindel J, Muruganandham M, Pigge FC, Anderson J, Kim Y. Magnetic resonance imaging (MRI) markers for MRI-guided high-dose-rate brachytherapy: novel marker-flange for cervical cancer and marker catheters for prostate cancer. Int J Radiat Oncol Biol Phys. 2013;86(2):387-93.
4. Scherman Rydhog J, Irming Jolck R, Andresen TL, Munck Af Rosenschold P. Quantification and comparison of visibility and image artifacts of a new liquid fiducial marker in a lung phantom for image-guided radiation therapy. Med Phys. 2015;42(6):2818-26.
5. Han BH, Wallner K, Merrick G, Badiozamani K, Butler W. The effect of interobserver differences in post-implant prostate CT image interpretation on dosimetric parameters. Med Phys. 2003;30(6):1096-102.
6. Roy JN, Wallner KE, Chiu-Tsao ST, Anderson LL, Ling CC. CT-based optimized planning for transperineal prostate implant with customized template. Int J Radiat Oncol Biol Phys. 1991;21(2):483-9.
7. Roy JN, Wallner KE, Harrington PJ, Ling CC, Anderson LL. A CT-based evaluation method for permanent implants: application to prostate. Int J Radiat Oncol Biol Phys. 1993;26(1):163-9.
8. Valente G, Pitto L, Testi D, Seth A, Delp SL, Stagni R, et al. Are subject-specific musculoskeletal models robust to the uncertainties in parameter identification? PLoS ONE. 2014;9(11):e112625.
9. Freilich JM, Spiess PE, Biagioli MC, Fernandez DC, Shi EJ, Hunt DC, et al. Lipiodol as a fiducial marker for image-guided radiation therapy for bladder cancer. Int Braz J Urol. 2014;40(2):190-7.
10. Aras MH, Miloglu O, Barutcugil C, Kantarci M, Ozcan E, Harorli A. Comparison of the sensitivity for detecting foreign bodies among conventional plain radiography, computed tomography and ultrasonography. Dentomaxillofac Radiol. 2010;39(2):72-8.
11. Kim SY, Park B, Kong IG, Choi HG. Analysis of ingested foreign bodies according to age, type and location: a retrospective observational study. Clin Otolaryngol. 2016;41(6):640-5.
12. Reddy N, Fata P, Balzer A, Diaz-Marchan P, Lincoln CM. Thorn in My Spine: A case of a retained intradural extramedullary foreign body. Clin Imaging. 2017;45:118-21.
13. Shelsta HN, Bilyk JR, Rubin PA, Penne RB, Carrasco JR. Wooden intraorbital foreign body injuries: clinical characteristics and outcomes of 23 patients. Ophthalmic Plast Reconstr Surg. 2010;26(4):238-44.
14. Li J, Zhou LP, Jin J, Yuan HF. Clinical diagnosis and treatment of intraorbital wooden foreign bodies. Chin J Traumatol. 2016;19(6):322-5.
15. Shokri A, Jamalpour M, Jafariyeh B, Poorolajal J, Sabet NK. Comparison of Ultrasonography, Magnetic Resonance Imaging and Cone Beam Computed Tomography for Detection of Foreign Bodies in Maxillofacial Region. J Clin Diagn Res. 2017;11(4):TC15-TC9.
16. Pattamapaspong N, Srisuwan T, Sivasomboon C, Nasuto M, Suwannahoy P, Settakorn J, et al. Accuracy of radiography, computed tomography and magnetic resonance imaging in diagnosing foreign bodies in the foot. Radiol Med. 2013;118(2):303-10.
17. McAllister AS, Lall NU, Tawfik KO, Jones BV. The imaging appearance of crayons. Pediatr Radiol. 2017;47(6):674-80.
18. Mundada P, Kohler R, Boudabbous S, Toutous Trellu L, Platon A, Becker M. Injectable facial fillers: imaging features, complications, and diagnostic pitfalls at MRI and PET CT. Insights Imaging. 2017;8(6):557-72.
19. Javadrashid R, Golamian M, Shahrzad M, Hajalioghli P, Shahmorady Z, Fouladi DF, et al. Visibility of Different Intraorbital Foreign Bodies Using Plain Radiography, Computed Tomography, Magnetic Resonance Imaging, and Cone-Beam Computed Tomography: An In Vitro Study. Can Assoc Radiol J. 2017;68(2):194-201.

For peer review only - http://bmjopen.bmj.com/site/about/guidelines.xhtml

20. Gilbert JW, Wheeler GR, Richardson GB, Herder SL, Mick GE, Gyarteng-Dakwa K, et al. Guidance of magnetic resonance imaging and placement of skin-marker localization devices. J Neurosurg Sci. 2011;55(2):85-8.

21. Havard AC, Collins DJ, Guy RL, Husband JE. Magnetic resonance behaviour of lipiodol. Clin Radiol. 1992;45(3):198-200.

For peer review only

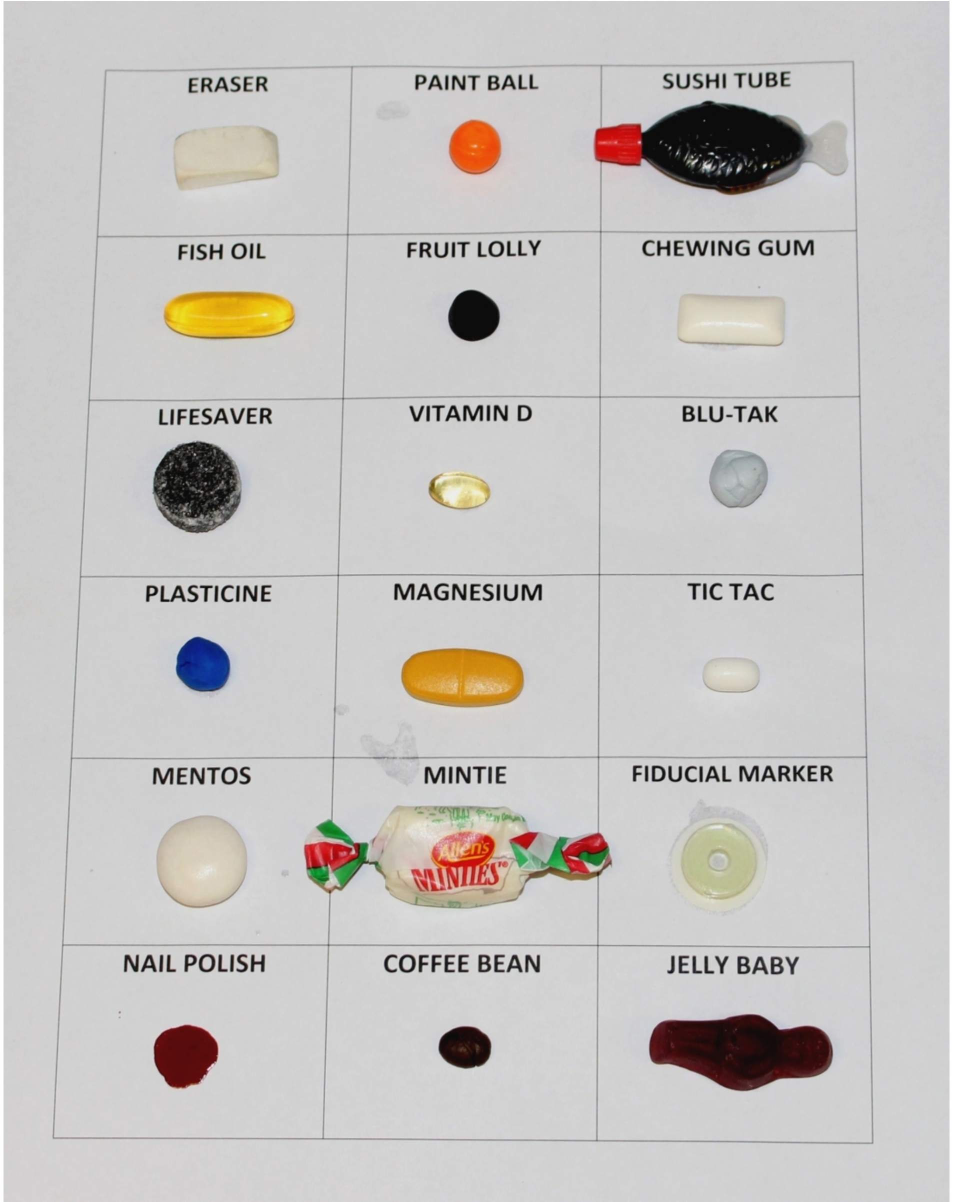

Figure 1. 18 items investigated as surrogates for the commercially available fiducial markers – Eraser, Paint Ball, Sushi Tube, Fish Oil capsule, Fruit lolly, Chewing gum, Lifesaver, Vitamin D, Blu-Tak, Plasticine, Magnesium tabsule, Tic Tac, Mentos, Mintie, Commercial Fiducial marker (IZI Multi-Modality, LifeHealthCare, Australia, included as a benchmark), Nail Polish, Coffee bean, Jelly baby sweet.

818x1036mm (72 x 72 DPI)

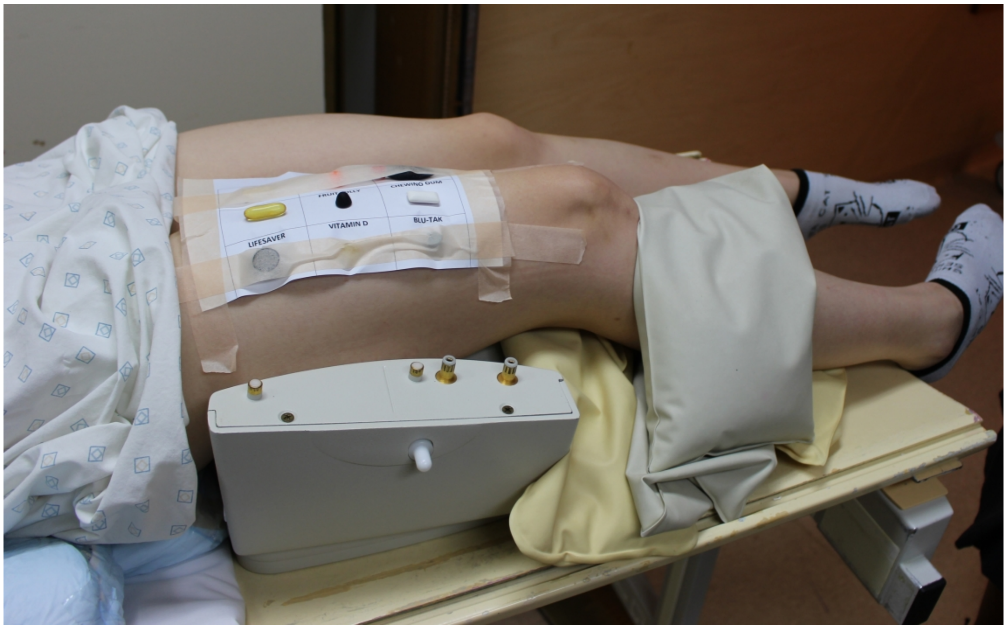

Figure 2. Markers were rigidly attached to the anterior surface of the thigh and the upper leg positioned in an RF coil. These images show the first nine items investigated.

1527x948mm (72 x 72 DPI)

Sequence	Proton Density	T1 Coronal	T1 3D	T2 Fat Sup	T2 3D
**Segmented Volume View of Anterior Thigh Surface**	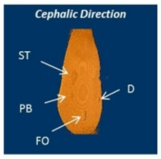	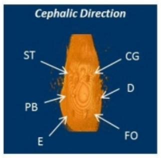	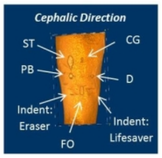	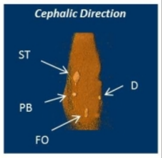	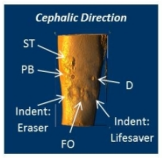
**Visible Markers**	• Fish Oil (FO)<br>• Vitamin D (D)<br>• Paint Ball (PB)<br>• Sushi Tube (ST)	• Fish Oil (FO)<br>• Vitamin D (D)<br>• Paint Ball (PB)<br>• Sushi Tube (ST)<br>• Chewing Gum (CG)<br>• Eraser (E)<br>*CG appeared to be visible on the 3D reconstruction but a 2D transverse slice at the level of CG did not demonstrate sufficient intensity contrast to differentiate the marker*	• Fish Oil (FO)<br>• Vitamin D (D)<br>• Paint Ball (PB)<br>• Sushi Tube (ST)<br>• Chewing Gum (CG)<br>*Indentations of the Lifesaver and Eraser were visible but the markers did not display*	• Fish Oil (FO)<br>• Vitamin D (D)<br>• Paint Ball (PB)<br>• Sushi Tube (ST)	• Fish Oil (FO)<br>• Vitamin D (D)<br>• Paint Ball (PB)<br>• Sushi Tube (ST)<br>*Indentation of the Lifesaver and Eraser are visible but marker does not display*

Figure 3. Segmented volume reconstructions of the anterior thigh surface, showing an example of markers that were visible for each MRI sequence for the first acquisition of 9 markers as shown in Figure 2.

319x140mm (150 x 150 DPI)

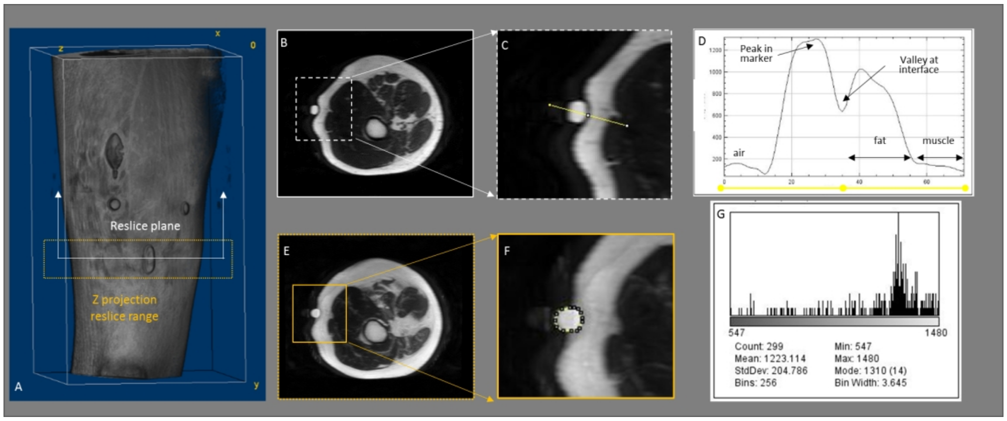

Figure 4. Workflow to measure relevant intensity parameters from each MRI dataset for each visible marker. A. 3D volume reconstruction of the T2, 3D sequence showing the transverse reslice plane (white line) and the range of z projection reslice plane (orange rectangle); B. Transverse reslice plane through the fish oil marker; C. Line selection across the marker (yellow unbroken line); D. Profile plot of intensity values along the line selection; E. Maximum intensity, transverse plane Z-projection of the fish oil capsule; F. Segmented selection tool demarcating the marker boundary (yellow broken line); G. Histogram detailing distribution of maximum intensities within the marker.

317x130mm (150 x 150 DPI)

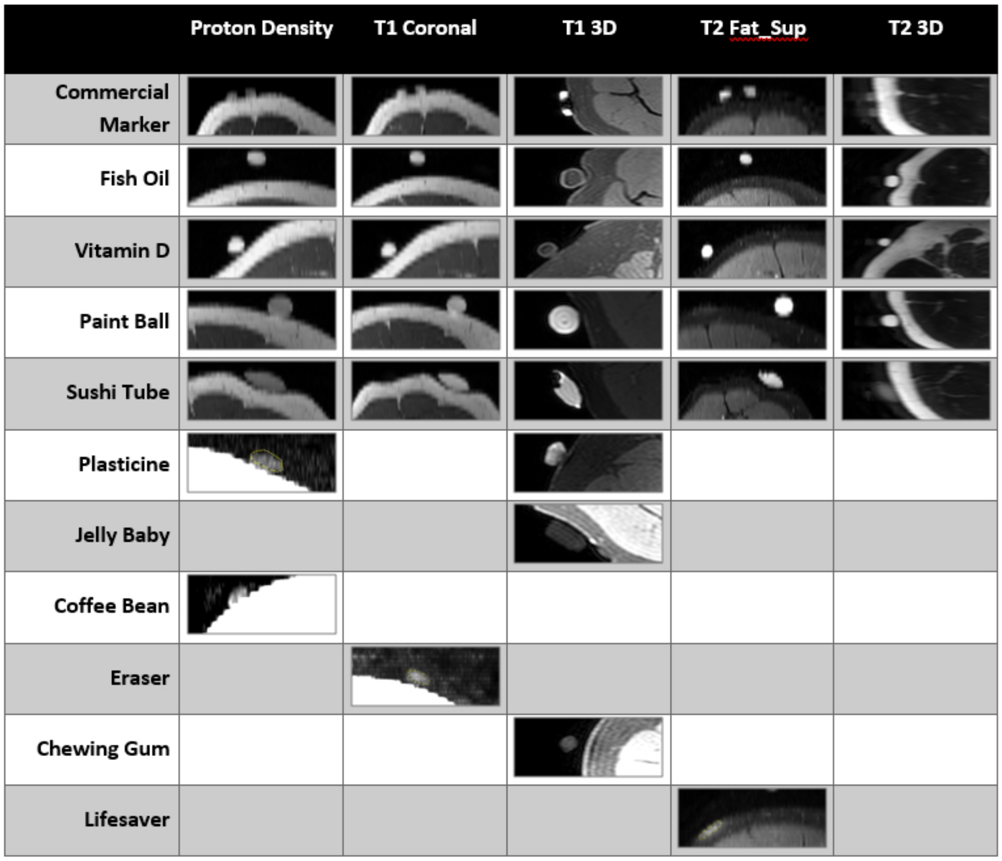

Figure 5: Reformatted transverse reslice images through the mid-height of all visible markers on the MR sequences analysed. Of the 18 markers, only 11 were visible on images from at least one of the MRI sequences – results for these 11 visible markers are shown. A blank box indicates that the marker tested was not clearly visible for a particular MR sequence.

201x173mm (96 x 96 DPI)

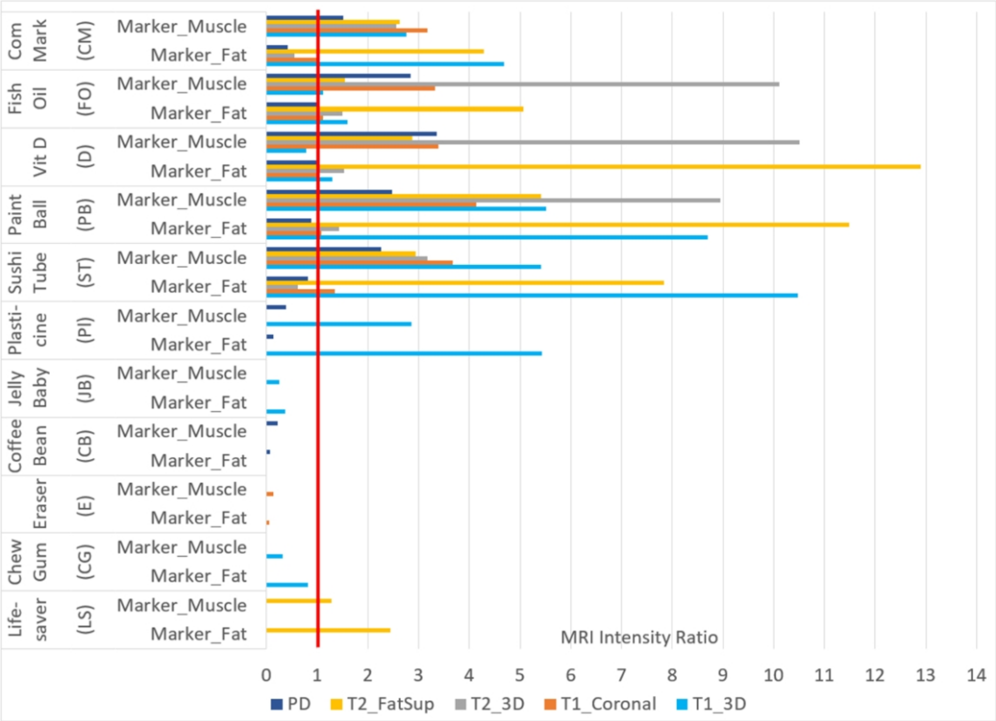

Figure 6: Intensity ratios for all visible markers. Marker_Muscle and Marker_Fat are the ratios between maximum marker intensity and mean intensity in the fat tissue and muscle tissue, respectively. Results are shown for the five MRI sequences: Proton Density (PD), T2 weighted fat-suppressed (T2_FatSup), 3D T2 weighted (T2_3D), T1 weighted coronal (T1_coronal) and 3D T1 weighted (T1_3D)

246x179mm (122 x 122 DPI)

Section & Topic	No	Item	Reported on page #
TITLE OR ABSTRACT		**Reliably Visible and Inexpensive surface Fiducial Marker options for Magnetic Resonance Imaging**	1
	1	Identification as a study of diagnostic accuracy using at least one measure of accuracy (such as sensitivity, specificity, predictive values, or AUC)	4
ABSTRACT			
	2	Structured summary of study design, methods, results, and conclusions (for specific guidance, see STARD for Abstracts)	2
INTRODUCTION			
	3	Scientific and clinical background, including the intended use and clinical role of the index test	3-4
	4	Study objectives and hypotheses	3-4
METHODS			
Study design	5	Whether data collection was planned before the index test and reference standard were performed (prospective study) or after (retrospective study)	Not applicable
Participants	6	Eligibility criteria	Not applicable
	7	On what basis potentially eligible participants were identified (such as symptoms, results from previous tests, inclusion in registry)	Not applicable
	8	Where and when potentially eligible participants were identified (setting, location and dates)	Not applicable
	9	Whether participants formed a consecutive, random or convenience series	Not applicable
Test methods	10a	Index test, in sufficient detail to allow replication	4-10
	10b	Reference standard, in sufficient detail to allow replication	4-10
	11	Rationale for choosing the reference standard (if alternatives exist)	4-10
	12a	Definition of and rationale for test positivity cut-offs or result categories of the index test, distinguishing pre-specified from exploratory	7-11
	12b	Definition of and rationale for test positivity cut-offs or result categories of the reference standard, distinguishing pre-specified from exploratory	7-11
	13a	Whether clinical information and reference standard results were available to the performers/readers of the index test	Not applicable
	13b	Whether clinical information and index test results were available to the assessors of the reference standard	Not applicable
Analysis	14	Methods for estimating or comparing measures of diagnostic accuracy	4-10
	15	How indeterminate index test or reference standard results were handled	Not applicable
	16	How missing data on the index test and reference standard were handled	Not applicable
	17	Any analyses of variability in diagnostic accuracy, distinguishing pre-specified from exploratory	Not applicable
	18	Intended sample size and how it was determined	Not applicable
RESULTS			
Participants	19	Flow of participants, using a diagram	Not applicable
	20	Baseline demographic and clinical characteristics of participants	Not applicable
	21a	Distribution of severity of disease in those with the target condition	Not applicable
	21b	Distribution of alternative diagnoses in those without the target condition	Not applicable
	22	Time interval and any clinical interventions between index test and reference standard	Not applicable
Test results	23	Cross tabulation of the index test results (or their distribution) by the results of the reference standard	11-16
	24	Estimates of diagnostic accuracy and their precision (such as 95% confidence intervals)	Not applicable
	25	Any adverse events from performing the index test or the reference standard	Not applicable
DISCUSSION			
	26	Study limitations, including sources of potential bias, statistical uncertainty, and generalisability	18
	27	Implications for practice, including the intended use and clinical role of the index test	15-17
OTHER INFORMATION			
	28	Registration number and name of registry	Not applicable
	29	Where the full study protocol can be accessed	4-10
	30	Sources of funding and other support; role of funders	17

For peer review only

STARD 2015

AIM

STARD stands for "Standards for Reporting Diagnostic accuracy studies". This list of items was developed to contribute to the completeness and transparency of reporting of diagnostic accuracy studies. Authors can use the list to write informative study reports. Editors and peer-reviewers can use it to evaluate whether the information has been included in manuscripts submitted for publication.

EXPLANATION

A **diagnostic accuracy study** evaluates the ability of one or more medical tests to correctly classify study participants as having a **target condition.** This can be a disease, a disease stage, response or benefit from therapy, or an event or condition in the future. A medical test can be an imaging procedure, a laboratory test, elements from history and physical examination, a combination of these, or any other method for collecting information about the current health status of a patient.

The test whose accuracy is evaluated is called **index test.** A study can evaluate the accuracy of one or more index tests. Evaluating the ability of a medical test to correctly classify patients is typically done by comparing the distribution of the index test results with those of the **reference standard**. The reference standard is the best available method for establishing the presence or absence of the target condition. An accuracy study can rely on one or more reference standards.

If test results are categorized as either positive or negative, the cross tabulation of the index test results against those of the reference standard can be used to estimate the **sensitivity** of the index test (the proportion of participants *with* the target condition who have a positive index test), and its **specificity** (the proportion *without* the target condition who have a negative index test). From this cross tabulation (sometimes referred to as the contingency or "2x2" table), several other accuracy statistics can be estimated, such as the positive and negative **predictive values** of the test. Confidence intervals around estimates of accuracy can then be calculated to quantify the statistical **precision** of the measurements.

If the index test results can take more than two values, categorization of test results as positive or negative requires a **test positivity cut-off**. When multiple such cut-offs can be defined, authors can report a receiver operating characteristic (ROC) curve which graphically represents the combination of sensitivity and specificity for each possible test positivity cut-off. The **area under the ROC curve** informs in a single numerical value about the overall diagnostic accuracy of the index test.

The **intended use** of a medical test can be diagnosis, screening, staging, monitoring, surveillance, prediction or prognosis. The **clinical role** of a test explains its position relative to existing tests in the clinical pathway. A replacement test, for example, replaces an existing test. A triage test is used before an existing test; an add-on test is used after an existing test.

Besides diagnostic accuracy, several other outcomes and statistics may be relevant in the evaluation of medical tests. Medical tests can also be used to classify patients for purposes other than diagnosis, such as staging or prognosis. The STARD list was not explicitly developed for these other outcomes, statistics, and study types, although most STARD items would still apply.

DEVELOPMENT

This STARD list was released in 2015. The 30 items were identified by an international expert group of methodologists, researchers, and editors. The guiding principle in the development of STARD was to select items that, when reported, would help readers to judge the potential for bias in the study, to appraise the applicability of the study findings and the validity of conclusions and recommendations. The list represents an update of the first version, which was published in 2003.

More information can be found on http://www.equator-network.org/reporting-guidelines/stard.

For peer review only - http://bmjopen.bmj.com/site/about/guidelines.xhtml
